# Effects of Cardiac Rehabilitation in Low- and Middle-Income Countries: A systematic Review and Meta-Analysis of Randomised Controlled Trials

**DOI:** 10.1016/j.pcad.2021.07.004

**Published:** 2022

**Authors:** Taslima Mamataz, Jamal Uddin, Sayed Ibn Alam, Rod S. Taylor, Maureen Pakosh, Sherry L. Grace, on behalf of the ACROSS collaboration

**Affiliations:** a Faculty of Health, York University, Bethune 222B, 4700 Keele Street, Toronto, ON M3J 1P3, Canada; b Department of Cardiac Surgery, Ibrahim Cardiac Hospital & Research Institute (ICHRI), Shahbag, Dhaka, Bangladesh; c Videncenter for Rehabilitering og Palliation REHPA, University of South Denmark, Nyborg, Denmark; d MRC/CSO Social and Public Health Sciences Unit & Robertson Centre for Biostatistics, Institute of Health and Well Being, School of Medicine, Dentistry & Nursing, University of Glasgow, UK; e Library & Information Services, Toronto Rehabilitation Institute, University Health Network, University of Toronto, Toronto, ON, Canada; f Faculty of Health, York University, Bethune 368, 4700 Keele Street, Toronto, ON M3J 1P3, Canada; g KITE & Peter Munk Cardiac Centre, University Health Network, University of Toronto, Toronto, ON, Canada

**Keywords:** Cardiac rehabilitation, Randomised controlled trial, Systematic review, Mortality, Morbidity, Quality of life, 6MWD, 6-min walk distance, AC, Active comparison, ACS, Acute coronary syndrome, AMSTAR, A Measurement Tool to Assess Systematic Reviews, BMI, Body mass index, BP, Blood pressure, CI, Confidence interval, CINAHL, Cumulative Index to Nursing & Allied Health Literature, CR, Cardiac rehabilitation, CV, Cardiovascular, CVDs, Cardiovascular diseases, DBP, Diastolic blood pressure, ECG, Electrocardiograms, GRADE, Grading of Recommendations, Assessment, Development and Evaluation, HDL- Cholesterol, High density lipoprotein cholesterol, HF, Heart failure, ICCPR, International Council of Cardiovascular Prevention and Rehabilitation, ICMJE, International Committee of Medical Journal Editors, LDL-Cholesterol, Low density lipoprotein cholesterol, LMICs, Low- and middle-income countries, MCS, Mental component summary, MD, Mean difference, METs, Metabolic equivalents of task, NYHA, New York Heart Association, PCS, Physical component summary, PICO, Population, intervention, comparison and outcomes, PRISMA, Preferred reporting items for systematic reviews and meta-analyses, QoL, Quality of life, RCTs, Randomised controlled trials, SF-12, Short form questionnaire-12, SF-36, Short form questionnaire-36, SBP, systolic blood pressure, SWiM, Synthesis Without Meta-analysis guideline, UC, Usual care, VO_2_peak, Peak oxygen consumption, WHO, World Health Organization

## Abstract

**Objectives:**

To assess the effectiveness of cardiac rehabilitation (CR) in low- and middle-income countries (LMICs), given previous reviews have included scant trials from these settings and the great need there.

**Methods:**

Six electronic databases (PubMed, Medline, Embase, CINAHL, Cochrane Library, and APA PsycINFO) were searched from inception-May 2020. Randomised controlled CR (i.e., at least initial assessment and structured exercise; any setting; some Phase II) trials with any clinical outcomes (e.g., mortality and morbidity, functional capacity, risk factor control and psychosocial well-being) or cost, with usual care (UC) control or active comparison (AC), in acute coronary syndrome with or without revascularization or heart failure patients in LMICs were included. With regard to data extraction and data synthesis, two reviewers independently vetted identified citations and extracted data from included trials; Risk of bias was assessed using Cochrane’s tool. Certainty of evidence was ascertained based on the Grading of Recommendations Assessment, Development and Evaluation (GRADE) framework. A random-effects model was used to calculate weighted mean differences and 95% confidence intervals (CI).

**Results:**

Twenty-six trials (6380 participants; 16.9% female; median follow-up = 3 months) were included. CR meaningfully improved functional capacity (VO_2peak_ vs UC: 5 trials; mean difference [MD] = 3.13 ml/kg/min, 95% CI = 2.61 to 3.65; I^2^ = 9.0%); moderate-quality evidence), systolic blood pressure (vs UC: MD = -5.29 mmHg, 95% CI = -8.12 to -2.46; I^2^ = 45%; low-quality evidence), low-density lipoprotein cholesterol (vs UC: MD = -16.55 mg/dl, 95% CI = -29.97 to -3.14; I^2^ = 74%; very low-quality evidence), body mass index (vs AC: MD = -0.84 kg/m^2^, 95% CI = -1.61 to −0.07; moderate-quality evidence; I^2^ = 0%), and quality of life (QoL; vs UC; SF-12/36 physical: MD = 6.05, 95% CI = 1.77 to 10.34; I^2^ = 93%, low-quality evidence; mental: MD = 5.38, 95% CI = 1.13 to 9.63; I^2^ = 84%; low-quality evidence), among others. There were no evidence of effects on mortality or morbidity. Qualitative analyses revealed CR was associated with lower percutaneous coronary intervention, myocardial infarction, better cardiovascular function, and biomarkers, as well as return to life roles; there were other non-significant effects. Two studies reported low cost of home-based CR.

**Conclusions:**

Low to moderate-certainty evidence establishes CR as delivered in LMICs improves functional capacity, risk factor control and QoL. While more high-quality research is needed, we must augment access to CR in these settings.

**Systematic review registration:**

PROSPERO (CRD42020185296).

## Introduction

The prevalence of cardiovascular (CV) diseases (CVD) in middle-income countries is growing alarmingly, from 4624 to 7769 per 100,000 people over the last thirty years^1^^,^^2^ CVDs are also among the leading causes of disability in low and middle-income countries (LMICs; 135/~200 countries worldwide), accounting for 21% of all disability-adjusted life years lost in 2019.^1^^,^^3^ Accordingly, CV care represents a major cost to health systems in these countries.^4^ Moreover, premature CVD mortality is higher in LMICs,^2^ representing a huge economic burden for families and national economies.

This burden can be substantially mitigated with proven secondary prevention approaches. Cardiac rehabilitation (CR) is a standardized outpatient model of care delivering risk factor management, structured exercise training, patient education, as well as heart-health behavior and psychosocial counselling.^5^ Cochrane meta-analyses of trials have established that participation in CR results in ~20% reductions in CVD mortality and morbidity, such as costly revascularizations and re-hospitalizations,^6^ as well as clinically-meaningful gains in quality of life (QoL),^6^^,^^7^ all while being cost-effective.^8^ Accordingly, guidelines for acute coronary syndrome (ACS)^9^ and heart failure (HF)^10^ patients strongly recommend referral to CR.

However, the majority of the evidence base for CR has been generated from high-income countries. Of the 63 trials included in the Cochrane review for ACS for example,^6^ only 5 were in LMICs, and of the 44 trials included in the review for HF,^11^ 4 were in LMICs. While it is expected –given the physiological mechanisms by which CR likely exerts its benefits – that CR in LMICs would be equally effective, this should be tested because: (1) patients in LMICs have less access to preventive and acute care, as well as medicines; and (2) CR may be implemented differently due to resource constraints and healthcare system characteristics.^12^^,^^13^ Indeed, despite the great need for CR in LMICs demonstrated above, availability is low^14^ and few patients have the opportunity to access it.^15^ In response, adaptations to CR have been implemented to reduce delivery cost and increase patient access, through exploiting technology to enable remote delivery or offering more appealing forms of exercise such as yoga for example.^16^^,^^17^ However, the effectiveness of CR in LMICs has never been established through meta-analysis of randomised controlled trials (RCT) to our knowledge.^16^^,^^17^ Therefore, the objectives of this systematic review were to assess the clinical effectiveness and cost-effectiveness of CR in LMICs for ACS and HF patients.

## Methods

This prospectively-registered review was undertaken in accordance with the Cochrane Handbook for Systematic Reviews of Interventions,^18^ and was reported in accordance with the Preferred Reporting Items for Systematic Reviews and Meta-Analyses (PRISMA) statement,^19^ and addresses the items outlined in the “A Measurement Tool to Assess Systematic Reviews” (AMSTAR) checklist.^20^

### Inclusion/Exclusion Criteria (PICOs) for Study Selection

We included reports published in peer-reviewed journals, and studies available as abstracts were excluded. The only included study design was RCTs, including cluster or cross-over designs; these were coded in terms of whether they had UC (usual care) or AC (active comparison) arms, or both. The trial could have any outcome, given this was the first meta-analysis in the area, but we were particularly interested in mortality and morbidity, functional capacity, risk factor control, as well as QoL.

Studies that included adult (age ≥ 18 years) patients with ACS (+/− revascularization [coronary artery bypass graft surgery or percutaneous coronary intervention]) and/or HF, living in a LMIC (as per World Bank)^21^ were included. Patients with other cardiovascular conditions could be included in the sample, as long as ≥50% of the sample had ACS or HF.

The intervention had to be exercise-based CR, defined as a supervised or unsupervised; outpatient, community- and/or home-based intervention; which included initial assessment and some form of structured exercise training (including yoga); either alone or in addition to psychosocial and/or educational interventions (note that the latter is a deviation from the posted protocol). The CR program had to include some phase II delivery (i.e., post-hospitalization).

### Search Strategy

Some of the authors collaborated on the previous scoping review of rehabilitation for non-communicable diseases in low-resource settings,^16^ which had similar but wider inclusion criteria (and did not report on outcomes). An experienced information specialist (MP) developed and performed the search for that and this review. That search went through October 2018. Search strategies were modeled on the PICO(S) framework, and utilized subject headings as appropriate for each database, as well as free-text terms relevant to the topical concepts. Trials included in that review were considered for inclusion in this one if they included ACS or HF patients (not stroke) as per above, were RCTs, and were conducted in a LMIC (not a low-resource setting in a high-income country).

For the update, the following 7 bibliographic databases were searched for studies published between October 2018 through to May 12th, 2020 in any language: Medline (Ovid), Pubmed (non-Medline), Embase, Global Index Medicus, PsycINFO, CINAHL (Cumulative Index to Nursing & Allied Health Literature), and EMCARE; search strategies were slightly modified from the previous review^16^ given the more restrictive criteria. A sample search strategy for Medline is shown in online Supplemental Appendix 1. The reference lists of any relevant reviews identified were hand-searched for potential articles.

### Trial Selection

Duplicate citations from across the databases were deleted in Mendeley software, with the unique citations then imported into Covidence. Two researchers independently considered the abstracts of potentially-eligible articles. The full-texts of potential citations were then considered to ascertain whether they met eligibility criteria; in some cases corresponding authors were contacted for information to make the inclusion decision. Any disagreements were resolved by the senior researchers for both stages. Once the trials were identified, we searched for any related protocol manuscripts or trial registry postings (World Health Organization [WHO], clinicaltrials.gov), theses/dissertations, or publications on the baseline cohort to inform data extraction and quality assessment.

### Data Extraction

The Cochrane data extraction template for RCTs was adapted. Two authors independently extracted relevant data characterizing study design (including type of comparator), participants, intervention features, risk of bias, and results into the word file, and outcomes were extracted to an excel spreadsheet. Included trials were also rated using Cochrane's Risk of Bias 1 tool. Any disagreements were resolved by discussion, or consultation with the senior author where consensus could not be reached.

Outcome data at all available follow-up points were extracted (the latest was used for analysis), and results based on intention-to-treat were pulled where available. Corresponding authors were contacted where needed to collect missing information. When post-treatment scores were not available, we extracted data according to the hierarchy of between-group differences and corresponding 95% confidence intervals (CI) at follow-up and then pre-treatment to post-treatment within-group change scores. When a study did not report standard deviations, we used estimation methods recommended by the Cochrane handbook.

### Data Synthesis

Where possible, meta-analysis was used to pool outcome results across studies. The authors created an excel file with all outcomes (including units of measurement/assessment tools) by comparison type (UC or AC), to determine whether there were ≥ 3 trials for any given outcome measured consistently with the same comparison type to perform meta-analysis. Note that lipid values were converted to mg/dl for consistency, and functional capacity measured with peak oxygen consumption (VO_2_peak) and 6-min walk distance (6MWD) were converted to metabolic equivalents of task (METs) to allow for meta-analysis where possible. Where QoL was measured using the SF-36, physical and mental component summary (PCS and MCS) scores were calculated where unreported; PCS and MCS scores for both SF-36 and SF-12 were included in the meta-analyses. We used a Synthesis Without Meta-analysis (SWiM) approach to assess the quantitative impact of CR on outcomes for which meta-analysis of effect estimates was not possible.^22^

Where possible, meta-analyses were performed using RevMan 5.4.1 version. Given the likely clinical heterogeneity of studies (e.g., differences in settings, population and CR intervention), we pre-specified that outcome data would be pooled using a random-effects model. Mean differences and 95% CIs for continuous outcomes, and relative risk and 95% CI for binary outcomes between intervention and control/comparison arms were computed. For each outcome, statistical heterogeneity was assessed using χ^2^ and I^2^ statistics.

Where there were outcome data across ≥10 trials, we performed univariate meta-regression to explore the following trial-level variables: CR duration (<12 vs ≥12 weeks), CR intervention dose (number of weeks x average number of sessions/week), delivery format (centre- vs. home-based/other), trial setting (single vs. multicentre), and overall risk of bias. Data analyses were undertaken using STATA v16.1.

To assess for reporting bias, we planned to look for funnel plot asymmetry where sufficient trials were identified. Egger's test was computed using Stata v16.1 where there were at least 10 trials as well. Finally, using the above information, Grading of Recommendations, Assessment, Development, and Evaluation (GRADE) was then used to determine level of evidence for each outcome.^23^

## Results

**Fig. 1:**
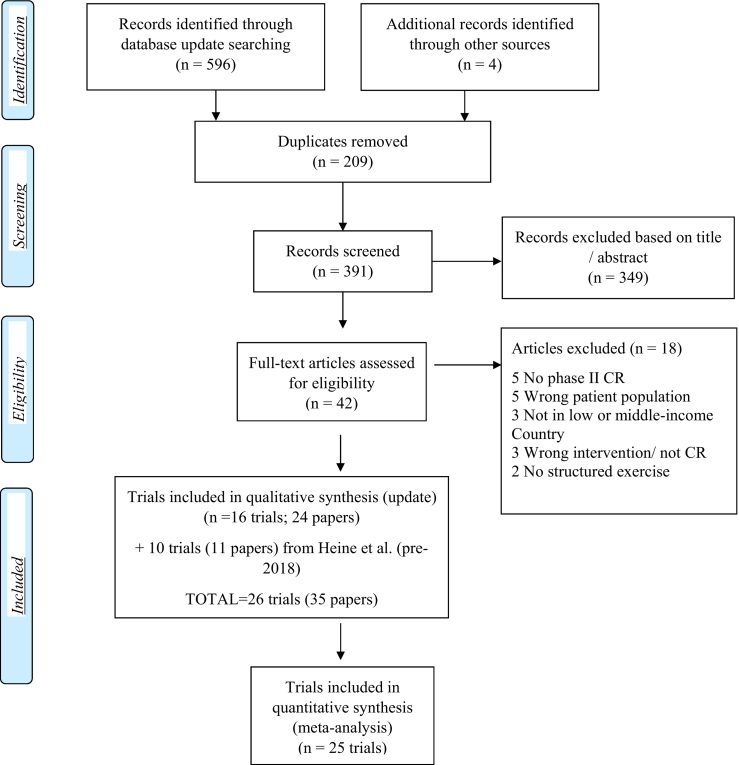
PRISMA flow diagram. CR, cardiac rehabilitation.

From the Heine et al. review,^16^ 15 trials in CVD patients were identified. Three were excluded as they were in stroke patients, 1 because it was a balance intervention,^24^ and 1 because there was no random allocation.^25^ It was also identified that the Erabelli et al.^26^^,^^27^ and Raghuram et al.^28^ citations were from the same cohort, so they were counted as 1 trial with multiple papers. Thus, there were 10 trials included pre-2018 (Fig. 1).

For the search update, the total number of citations identified, and the process which culminated in identifying 16 additional included trials is shown in Fig. 1. No non-English trials were identified. Some pre-specified outcomes from Dorje's trial,^29^ including mortality and morbidity, are currently in preparation (personal communication with corresponding author), and thus we only report on available outcomes. There were additional citations identified for the Raghuram trial identified in the earlier search as well.^26^ Thus overall, 26 trials were included (35 publications). A summary of included trials is shown in Tables 1 (design) and 2 (interventions).

**Table 1:** Summary of included trial design and results.

Study Author/Trial, Year, Country, Regionǂ	Participants/Sample (Size; mean age; proportion female; proportion HF and/or non-ACS participants)	Design: # arms, # sites	UC and/or AC comparison (dose, content)	Outcomes/Results (months of follow-up)
Abolahrari-Shirazi 2018,^38^ Iran, EMR;	*N* = 75; mean age 57.2 years; 25.3% female; 0.0% HF and/or non-ACS patients included	PROBE, 3 parallel arms; 1 site.	UC-Y (This group received only a pamphlet for daily exercising at home. Standard of care for Iranian adults with CVD does not include access to CR for all patients. All participants have follow-up appointments with their physician as deemed medically appropriate.), AC-Y (21 sessions [=3×/wk. for 7wks] moderate intensity; endurance exercise; 45 min; other components: not specified)	ITT: y Adverse events (7 weeks) CR 2 angina attacks (8.0%); AC 2 (1 severe hypotension and 1 non-cardiac hospitalization) (8.0%); UC 0 (0.0%). PVCs 6 (8.0%) (not specified what arm). No significant difference between groups. Functional Capacity (METs) (7 weeks) pre-CR 8.46 ± 1.89; post-CR 10.81 ± 1.76; pre-AC 7.38 ± 1.99; post-AC 10.07 ± 2.19 pre-UC 8.51 ± 2.11; post-UC 9.03 ± 2.10. Significant difference between and within groups (*P* < 0.001) following the intervention. Exercise test duration (min) (7 weeks) pre-CR 7.43 ± 1.89; post-CR 9.66 ± 1.71; pre-AC 6.28 ± 2.16; post-AC 8.93 ± 2.15; pre-UC 7.43 ± 2.19; post-UC 7.88 ± 2.13. Significant difference between and within groups following intervention (*p* < 0.001) except within UC groups. Peak HR (bpm) (7 weeks) pre-CR 138.0 ± 17.93; post-CR 144.11 ± 18.20; pre-AC 136.40 ± 22.21; post-AC 144.44 ± 27.07; pre-UC 136.29 ± 17.38; post-UC 129.49 ± 26.48. Significant difference within CR group after intervention (*p* = 0.005) and between groups (*p* = 0.01). Rate pressure product at Stage 2 of exercise test (S2RPP) (mm Hg bpm x1000) (7 weeks) pre-CR 16.66 ± 2.08; post-CR 15.18 ± 2.62; pre-AC 17.06 ± 2.80; post-AC 15.75 ± 2.68; pre-UC 15.51 ± 3.06; post-UC 15.61 ± 3.69. No significant difference between and within groups except within AC group following intervention (*p* = 0.04). Cardiovascular Biomarkers NT-proBNP (pg/ml) (7 weeks) pre-CR 204.42 ± 176.40; post-CR 136.74 ± 151.93; pre-AC 264.62 ± 182.59; post-AC 149.55 ± 117.41; pre-UC 221.17 ± 165.72; post-UC 189.80 ± 152.91. Significant difference within CR (*P* = 0.004), AC groups (*P* = 0.002) following intervention. No significant difference between groups. Hs-CRP (mg/L) (7 weeks) pre-CR 1.53 ± 1.98; post-CR 1.09 ± 1.49; pre-AC 1.52 ± 2.55; post-AC 1.05 ± 1.66; pre-UC 1.28 ± 1.34; post-UC 1.44 ± 1.96. No significant difference between and within groups.
Abdel-Halim 2018,^43^ Egypt, EMR	*N* = 40; mean age 53.3 years; 15.0% female; 0.0% HF and/or non-ACS patients included	PROBE. 2 parallel arms; 1 site.	UC-No; AC-Y (24 sessions [2×/wk. for 12 wks]; moderate intensity; aerobic exercise; 45 min; other components: pt. education)	ITT: y Functional Capacity (METs) (3 months) pre—CR 7.60 ± 2.14; post-CR 11.55 ± 1.47; pre-AC 8.35 ± 2.06; post-AC 10.90 ± 2.65. Significant difference within groups (*p* < 0.001) and no significant difference between groups following the intervention. Total cholesterol (mg/dL) (3 months) pre-CR 185.7 ± 23.46; post-CR 163.90 ± 20.57; pre-AC 119.35 ± 47.98; post-AC 151.20 ± 45.75. Significant difference within groups (*p* < 0.001) and no significant difference between groups following the intervention. HDL-C (mg/dL) (3 months) pre-CR 35.05 ± 4.49; post-CR 41.00 ± 3.83; pre-AC 40.28 ± 13.91; post-AC 37.80 ± 6.61. Significant difference within groups (*p* < 0.001) and no significant difference between groups following the intervention. LDL-C (mg/dL) (3 months) pre-CR 94.45 ± 15.41; post-CR 84.45 ± 11.56; pre-AC 105.95 ± 22.78; post-AC 92.41 ± 26.72. Significant difference within groups (*p* < 0.001) and no significant difference between groups following the intervention. Triglyceride (mg/dL) (3 months) pre-CR 138.60 ± 33.47; post-CR 118.95 ± 27.02; pre-AC 168.25 ± 40.92; post-AC 103.40 ± 35.08. Significant difference within groups (*p* < 0.001) and no significant difference between groups following the intervention. Serum Creatinine (mg/dl) (3 months) pre-CR 1.27 ± 0.32; post-CR 1.09 ± 0.26; pre-AC 1.00 ± 0.20; post-AC 0.99 ± 0.17. No significant difference between and within groups. Hemoglobin level (gm/dl) (3 months) pre-CR 12.82 ± 1.35; post-CR 13.23 ± 1.59; pre-AC 12.18 ± 1.46; post-AC 12.14 ± 1.18. No significant difference between and within groups. Platelet count (3 months) pre-CR 244.45 ± 58.17; post-CR 232.25 ± 50.35; pre-AC 250.40 ± 82.73; post-AC 251.80 ± 76.62. No significant difference between and within groups. WBCs (x 10^9^/L) (3 months) pre-CR 8.72 ± 2.69; post-CR 6.45 ± 1.43; pre-AC 7.31 ± 2.20; post-AC 6.82 ± 1.44. No significant difference between and within groups. Quality of life (SF-36) (3 months)* i) General Health pre-CR 253.75 ± 24.70; post-CR 345.00 ± 33.05; pre-AC 256.25 ± 29.10; post-AC 356.25 ± 31.28. Significant difference within groups (*p* < 0.001) and no significant difference between groups following the intervention. ii)Physical function pre-CR 625.00 ± 34.41; post-CR 747.50 ± 41.28; pre-AC 637.50 ± 42.53; post-AC 757.50 ± 40.64. Significant difference within groups (*p* < 0.001) and no significant difference between groups following the intervention. iii)Emotional well-being pre-CR 283.00 ± 20.80; post-CR 398.00 ± 15.76; pre-AC 273.00 ± 31.97; post-AC 377.00 ± 31.30. Significant difference between (*p* = 0.01) and within groups (p < 0.001) following intervention. iv)Total score pre-CR 1832.50 ± 109.85; post-CR 3026.50 ± 79.08; pre-AC 1781.25 ± 121.37; post-AC 2967.25 ± 84.03. No significant difference between (*p* = 0.02) and within groups (p < 0.001) following intervention. EF (%) (3 months) pre-CR 43.30 ± 5.32; post-CR 48.30 ± 5.72; pre-AC 43.85 ± 5.30; post-AC 48.25 ± 5.44. Significant difference within groups (p < 0.001) and no significant difference between groups following the intervention.
Ajiboye 2015,^55^ Nigeria, Africa	*N* = 69; mean age 54.0 years; 55.0% female; 100.0% HF and no non-ACS patients included	PROBE, 2 parallel arms; 1 site.	UC-Y (standard pharmacological treatment at the HF clinic, and encouraged to continue their usual activity levels but not to initiate any new exercise training during the 12-week study period.), AC comparison-No	ITT: no Adverse events (3 months) Signs of decompensation CR 2 (5.7%) UC 1 (2.9%); Functional Capacity (6MWD in meters) (3 months) pre-CR 414.10 ± 46.57; post-CR 448.8 ± 34.92; pre-UC 404.00 ± 33.09; post-UC 399.00 ± 58.03; Significant difference between (*p* < 0.001) and within groups (p < 0.001) following the intervention. Functional Capacity (PVO_2_-DASI) (3 months) pre-CR 12.80 ± 1.06; post-CR 15.00 ± 1.06; pre-UC 12.70 ± 0.96; post-UC 12.20 ± 0.96; Significant difference between (p < 0.001) and within groups (p < 0.001) following the intervention. Functional Capacity (PVO_2_-VSAQ) (3 months) pre-CR 13.80 ± 2.12; post-CR 21.90 ± 2.65; pre-UC 14.90 ± 2.40; post-UC 14.10 ± 2.40; Significant difference between (p < 0.001) and within groups (p < 0.001) following the intervention. Resting SBP (mm of Hg) (3 months) pre-CR 126.50 ± 8.99; post-CR 120.90 ± 9.52; pre-UC 119.20 ± 15.84; post-UC 122.30 ± 12.48; No significant difference between and within groups following the intervention. Resting DBP (mm of Hg) (3 months) pre-CR 81.80 ± 10.58; post-CR 79.50 ± 7.94; pre-UC 77.90 ± 13.44; post-UC 80.10 ± 12.96; No significant difference between and within groups following the intervention. Resting HR (bpm) (3 months) pre-CR 80.60 ± 9.52; post-CR 74.00 ± 22.22; pre-UC 73.80 ± 10.08; post-UC 74.10 ± 10.56; Significant difference between groups (*p* = 0.03) following the intervention. Resting RR (breaths/min) (3 months) pre-CR 24.40 ± 4.23; post-CR 22.10 ± 3.70; pre-UC 23.50 ± 5.28; post-UC 24.30 ± 5.28; Significant difference between groups (*p* = 0.004) following the intervention. Rating of perceived exertion (modified Borg scale) (3 months) pre-CR 4.30 ± 1.06; post-CR 2.10 ± 1.06; pre-UC 4.10 ± 0.48; post-UC 4.20 ± 0.96; Significant difference between groups (p < 0.001) following the intervention. Resting blood oxygen saturation (%) (3 months) pre-CR 97.00 ± 1.06; post-CR 98.30 ± 0.53; pre-UC 96.50 ± 1.92; post-UC 96.80 ± 1.44; Significant difference between groups (p < 0.001) following the intervention.
Aslanabadi 2008,^56^ Iran, EMR	*N* = 100; mean age 54.0 years; 16.0% female; 0.0% HF and/or non-ACS patients included	PROBE with 2 parallel arms; 1 site.	UC- Y (the standard of care for cardiovascular patients in Iran includes physician's consultation as required); AC —No	ITT: NR SBP (mmHg) (24 months) pre-CR 138.00 ± 24.00; post-CR 130.00 ± 21.00; pre-UC 139.00 ± 23.00; post-UC 139.00 ± 21.00; There is no significant difference within and between groups following intervention. DBP (mmHg) (24 months) pre-CR 84.00 ± 10.00; post-CR 78.00 ± 8.00; pre-UC 91.00 ± 12.00; post-UC 88.00 ± 10.00; There is no significant difference within and between groups following intervention. Total cholesterol (mg/dL) (24 months) pre-CR 212.90 ± 50.00; post-CR 188.80 ± 43.00; pre-UC 195.00 ± 45.00; post-UC 194.50 ± 47.00; Significant difference within CR group (*p* < 0.05) and between groups (p < 0.05) following intervention. HDL-C (mg/dL) (24 months) pre-CR 37.40 ± 13.00; post-CR 38.90 ± 13.00; pre-UC 41.10 ± 15.00; post-UC 41.20 ± 14.00; Significant difference within CR group (*p* < 0.05) and no significant difference between groups following intervention. LDL-C (mg/dL) (24 months) pre-CR 146.00 ± 45.00; post-CR 134.80 ± 38.00; pre-UC 174.00 ± 53.00; post-UC 141.80 ± 40.00; Significant difference within CR (p < 0.05) and UC (p < 0.05) groups and no significant difference between groups following intervention. Triglycerides (mg/dL) (24 months) pre-CR 255.00 ± 70.00; post-CR 177.60 ± 121.00; pre-UC 227.00 ± 129.00; post-UC 180.70 ± 118.00; Significant difference within CR (*p* < 0.05) and UC (p < 0.05) groups and between groups (p < 0.05) following intervention. Body mass index (kg/m2) (24 months) pre-CR 28.10 ± 4.00; post-CR 25.80 ± 2.00; pre-UC 27.20 ± 4.00; post-UC 26.90 ± 3.00; Significant difference within CR (p < 0.05) and UC (p < 0.05) groups and between groups (p < 0.05) following intervention. Waist-to-hip ratio (24 months) pre-CR 1.01 ± 0.30; post-CR 0.98 ± 0.10; pre-UC 0.99 ± 0.20; post-UC 0.99 ± 0.10; Significant difference within CR (p < 0.05) and UC (p < 0.05) groups and between groups (p < 0.05) following intervention. HR (bpm) (24 months) pre-CR 78.65 ± 6.00; post-CR 75.85 ± 4.00; pre-UC 73.40 ± 3.00; post-UC 73.00 ± 4.00; Significant difference within CR group (*p* < 0.05) following intervention. FBS (mg/dL) (24 months) pre-CR 128.10 ± 21.00; post-CR 115.80 ± 18.00; pre-UC 135.00 ± 23.00; post-UC 134.70 ± 23.00; Significant difference within CR (*p* < 0.05) group and between groups (p < 0.05) following intervention. Tobacco Use (24 months) pre-CR 15 (30.00%); post-CR 5 (10.00%); pre-UC 5 (10.00%); post-UC 15 (30.00%). Significant difference within CR group (p < 0.05) following intervention. Lifestyle behaviors (24 months) Diet type- Veg. (%) pre-CR 13 (26.00%); post-CR 30 (60.00%); pre-UC 24 (48.00%); post-UC 15 (30.00%); Significant difference within CR group (p < 0.05) following intervention. Diet type- Non veg. (%) pre-CR 25 (50.00%); post-CR 6 (12.00%); pre-UC 9 (18.00%); post-UC 25 (50.00%); No significant difference between and within groups. Diet type- occasional non veg. (%) pre-CR 12 (24.00%); post-CR 14 (28.00%); pre-UC 17 (34.00%); post-UC 10 (20.00%); No significant difference between and within groups. Eat low fat (%Yes) pre-CR 17 (34.00%); post-CR 36 (72.00%); pre-UC 19 (38.00%); post-UC 26 (52.00%); No significant difference between and within groups. Type of oil consumed- Saturated pre-CR 13 (26.00%); post-CR 0 (0.00%); pre-UC 12 (24.00%); post-UC 9 (18.00%); Significant difference within CR group (p < 0.05) and no significant difference between groups following intervention. Type of oil consumed- Unsaturated pre-CR 5 (10.00%); post-CR 24 (48.00%); pre-UC 4 (8.00%); post-UC 5 (10.00%); No significant difference between and within groups. Type of oil consumed- Both pre-CR 32 (64.00%); post-CR 26 (52.00%); pre-UC 34 (68.00%); post-UC 36 (72.00%); No significant difference between and within groups. Physical activity (exercise vigorously 20 min 3 times per week) (% Yes) pre-CR 10 (20.00%); post-CR 44 (88.00%); pre-UC 11 (22.00%); post-UC 10 (20.00%); Significant difference within and between (p < 0.05) CR group following intervention. Determine activity by monitoring HR (% Yes) pre-CR 14 (28.00%); post-CR 41 (82.00%); pre-UC 13 (26.00%); post-UC 18 (36.00%); Significant difference within CR group (p < 0.05) following intervention.
Babu 2011,^44^ India, SEA	*N* = 30; mean age 57.7 years; 26.7% female; 100.0% HF and no non-ACS patients included	PROBE with 2 parallel arms; 1 site.	UC-Y (The standard of care for CVD in India include patients are under regular follow-up of physicians and cardiologists as deemed medically appropriate.), AC —No	ITT: no CVD mortality (%) (9 weeks) CR 0 (0.0%); UC 1 (6.6%); No significant difference between groups. Hospitalization (%) (9 weeks) CR 0 (0.0%); UC 1 (6.6%); No significant difference between groups. Functional Capacity (6MWD in meters) (9 weeks) Pre-CR 429.33 ± 125.15; post-CR 514.53 ± 135.12; pre-UC 310.23 ± 121.11; post-UC 357.15 ± 147.95; Significant difference between CR and UC groups (P < 0.01) following the intervention. Quality of life (SF-36) (9 weeks) i) PCS pre-CR 35.30 ± 1.83; post-CR 49.53 ± 1.76; pre-UC 35.59 ± 2.12; post-UC 41.01 ± 2.14; Significant difference within groups (*p* < 0.001 for CR and *p* = 0.004 for UC group) and between groups (*p* = 0.002) following the intervention. ii) MCS pre-CR 33.79 ± 5.80; post-CR 47.49 ± 6.01; pre-UC 30.41 ± 9.27; post-UC 35.45 ± 5.70; Significant difference observed only within CR group (p < 0.001) and between groups (*p* = 0.003) following the intervention.
Chanrdrasekaran/Prabhakaran/Christa 2019,^33^,^47^,^57^ India, SEA	*N* = 3959; mean age 53.4 years; 14.0% female; 0.0% HF and /or non-ACS patients included	PROBE with 2 parallel arms; 24 sites.	UC- Y (The standard of care for HF in India include patients are under regular follow-up of physicians and cardiologists as deemed medically appropriate); AC comparison-No	ITT: y All-cause mortality (%) (3 months) CR 77 (3.94%); UC 77 (3.91%); No significant difference between groups. Non-fatal MI (%) (3 months) CR 13 (0.98%); UC 15 (2.10%); No significant difference between groups. CVD Hospitalization (%) (3 months) CR 48 (0.98%); UC 59 (2.10%); No significant difference between groups. Non-cardiac Hospitalization (%) (3 months) CR 24 (0.98%); UC 26 (2.10%); No significant difference between groups. Non-fatal Stroke (%) (3 months) CR 4 (0.98%); UC 3 (2.10%); No significant difference between groups. Adverse events (%) (3 months) CR 131 (0.98%); UC 146 (2.10%); No significant difference between groups. Self-rated Health (Visual Analogue scale of EQ-5D-5L) (3 months) pre-CR 66.30 ± 17.30; post-CR 77.00 ± 16.80; pre-UC 66.70 ± 17.00; post-UC 75.70 ± 17.80; Significant difference between groups (p = 0.002) following intervention. Return to pre-infarct activities (Reintegration to Normal Life Index questionnaire) (3 months) pre-CR -NR; post-CR 88.30 ± 18.90; pre-UC -NR; post-UC 87.00 ± 20.10; Significant difference between groups (*p* = 0.03) following intervention. Medication Adherence (“high” on Morisky scale) (3 months)‡ pre-CR-NR; post-CR 1199 (64.60%); pre-UC-NR; post-UC 1210 (64.30%); Significant difference within CR (*p* = 0.007) and UC (*p* = 0.003) groups and no difference between groups following intervention. Tobacco Use (%) (3 months) pre-CR 610 (31.01%); post-CR 449 (22.99%); pre-UC 592 (29.80%); post-UC 445 (22.61%); No significant difference between and within groups. Health State (EQ-5D-5L) (3 months) pre-CR-NR; post-CR 6.30 ± 2.90; pre-UC-NR; post-UC 6.50 ± 3.10; No significant difference within and between groups following intervention. Heart Rate Variability (3 months) 1)Time domain Indices i)SDNN (ms) pre-CR 151.24 ± 30.35; post-CR 163.04 ± 38.01; pre-UC 154.21 ± 29.92; post-UC 167.43 ± 38.9; Significant difference within CR (*p* = 0.007) and UC (*p* = 0.003) groups and no difference between groups following intervention ii)SDSD (ms) pre-CR 151.24 ± 30.35; post-CR 163.04 ± 38.01; pre-UC 154.21 ± 29.92; post-UC 167.43 ± 38.9; Significant difference within CR (p = 0.007) and UC (p = 0.003) groups and no difference between groups following intervention iii)RMSSD (ms) pre-CR 151.24 ± 30.35; post-CR 163.04 ± 38.01; pre-UC 154.21 ± 29.92; post-UC 167.43 ± 38.9; Significant difference within CR (p = 0.007) and UC (p = 0.003) groups and no difference between groups following intervention iv)pNN50 (%) pre-CR 151.24 ± 30.35; post-CR 163.04 ± 38.01; pre-UC 154.21 ± 29.92; post-UC 167.43 ± 38.9; Significant difference within CR (p = 0.007) and UC (p = 0.003) groups and no difference between groups following intervention 2) Frequency domain Indices Low Frequency (LF) Power pre-CR 151.24 ± 30.35; post-CR 163.04 ± 38.01; pre-UC 154.21 ± 29.92; post-UC 167.43 ± 38.9; Significant difference within CR (p = 0.007) and UC (p = 0.003) groups and no difference between groups following intervention High Frequency (HF) Power pre-CR 151.24 ± 30.35; post-CR 163.04 ± 38.01; pre-UC 154.21 ± 29.92; post-UC 167.43 ± 38.9; Significant difference within CR (p = 0.007) and UC (p = 0.003) groups and no difference between groups following intervention Total Power pre-CR 151.24 ± 30.35; post-CR 163.04 ± 38.01; pre-UC 154.21 ± 29.92; post-UC 167.43 ± 38.9; Significant difference within CR (p = 0.007) and UC (p = 0.003) groups and no difference between groups following intervention LF/HF Ratio pre-CR 151.24 ± 30.35; post-CR 163.04 ± 38.01; pre-UC 154.21 ± 29.92; post-UC 167.43 ± 38.9; Significant difference within CR (p = 0.007) and UC (p = 0.003) groups and no difference between groups following intervention.
Chaves/Britto/Ghisi, 2019,^37^,^35^,^36^,^58^ Brazil, AMR	*N* = 115; mean age 59.5 years; 28.7% female; 0.0% HF and/or non-ACS patients included	PROBE with 3 parallel arms; 1 site.	UC-Y (The standard of care for Brazilian adults with CVD does not include access to CR for all patients. All participants have follow-up appointments with their physician as deemed medically appropriate.), AC comparison-Y (36 exercise-only[=decreasing frequency from 3×/wk. to 1×/wk. for 24 wks]; moderate intensity; aerobic and resistance both; 60 min; other components: no)	ITT: y All-cause mortality (%) (6 months) CR 0 (0.0%); AC 0 (0.0%); UC 0 (0.0%); No significant difference between groups. Non-fatal MI (%) (6 months) UC 4 (10.0%); CR 1 (2.7%); AC 0 (0.0%); Significant difference between AC and UC (*P* < 0.01). Angina (%) (6 months) CR 9 (24.3%); UC 4 (10.0%); AC 2(5.1%); No significant difference between groups. CABG (%) (6 months) CR 1 (2.7%); AC 1 (2.5%); UC 0 (0.0%); No significant difference between groups. PCI (%) (6 months) UC 3 (7.6%); CR 0 (0.0%); AC 0 (0.0%); Significant difference between CR vs UC (*P* < 0.05) and AC vs UC groups (P < 0.05). Adverse events (%) (6 months) UC 9 (23.0%); AC 8 (20.5%); CR 3 (8.1%); No significant difference between groups. Hospitalizations (%) (6 months) UC 8 (20.5%); CR 3 (8.1%); AC 1 (2.5%); Significant difference between AC and UC groups (P < 0.01). Functional Capacity (ISWD in meters) (6 months) pre-CR 381.1 ± 120.9; post-CR 465.9 ± 115.4; pre-AC 361.0 ± 119.5; post-AC 432.3 ± 119.5 pre-UC 376.4 ± 145.6; post-UC 390.3 ± 160.5. Significant difference between CR and UC groups (P < 0.01) following the intervention. SBP (mmHg) (6 months) pre-CR 123.8 ± 15.1; post-CR 117.6 ± 19.8; pre-AC 117.3 ± 24.7; post-AC 117.4 ± 17.0; pre-UC 117.9 ± 17.6; post-UC 117.7 ± 19.1. No significant difference between and within groups. DBP (mmHg) (6 months) pre-CR 77.0 ± 11.0; post-CR 75.3 ± 12.6; pre-AC 77.7 ± 13.0; post-AC 77.8 ± 12.6; pre-UC 74.6 ± 16.0; post-UC 75.9 ± 15.3. No significant difference between and within groups. Total cholesterol (mg/dL) (6 months) pre-CR 165.0 ± 61.9; post-CR 165.8 ± 62.0; pre-AC 148.7 ± 39.4; post-AC 153.2 ± 43.8; pre-UC 152.8 ± 34.6; post-UC 154.7 ± 36.2. No significant difference between and within groups. HDL-C (mg/dL) (6 months) pre-CR 39.5 ± 7.9; post-CR 39.7 ± 7.6; pre-AC 40.4 ± 14.3; post-AC 38.1 ± 9.0; pre-UC 42.0 ± 7.1; post-UC 42.2 ± 7.1. No significant difference between and within groups. LDL-C (mg/dL) (6 months) pre-CR 86.4 ± 29.7; post-CR 87.3 ± 30.6; pre-AC 80.4 ± 23.7; post-AC 83.9 ± 29.7; pre-UC 82.5 ± 30.2; post-UC 83.3 ± 30.8. No significant difference between and within groups. Triglyceride (mg/dL) (6 months) pre-CR 166.0 ± 117.0; post-CR 165.3 ± 114.2; pre-AC 137.7 ± 75.2; post-AC 150.2 ± 89.9; pre-UC 141.3 ± 51.3; post-UC 145.4 ± 51.8. No significant difference between and within groups. Blood glucose (mg/dL) (6 months) pre-CR 104.6 ± 20.2; post-CR 100.7 ± 17.2; pre-AC 107.2 ± 35.3; post-AC 111.1 ± 32.5; pre-UC 109.9 ± 38.3; post-UC 104.9 ± 25.7. Significant difference between pre and post-CR (*P* = 0.02). Waist circumference (cm) (6 months) pre-CR 96.0 ± 11.5; post-CR 95.6 ± 11.9; pre-AC 96.7 ± 10.6; post-AC 95.6 ± 10.9; pre-UC 94.9 ± 9.8; post-UC 94.8 ± 9.9. No significant difference between and within groups. Body mass index (kg/m^2^) (6 months) pre-CR 28.1 ± 4.2; post-CR 28.1 ± 4.5; pre-AC 28.7 ± 6.0; post-AC 28.9 ± 6.9; pre-UC 27.8 ± 4.0; post-UC 27.8 ± 3.8. No significant difference between and within groups. Diet (FFQ) (6 months) pre-CR 4.7 ± 7.7; post-CR 7.8 ± 7.1; pre-AC 5.9 ± 7.4; post-AC 6.5 ± 6.9; pre-UC 7.9 ± 6.9; post-UC 6.9 ± 5.9. Significant difference between CR vs UC (*P* < 0.01) and CR vs AC groups (P < 0.01). No significant difference within groups. Physical Activity/Exercise (7-day Pedometer use, daily mean) (6 months) pre-CR 4487.9 ± 3416.9; post-CR 5422.0 ± 4284.7; pre-AC 4736.2 ± 3948.1; post-AC 4996.8 ± 4504.4; pre-UC 4426.5 ± 2399.0; post-UC 3922.3 ± 2571.1. No significant difference between and within groups. Tobacco Use (%) (6 months) pre-CR 2 (5.4%); post-CR 2 (5.4%); pre-AC 4 (10.4%); post-AC 4 (10.4%); pre-UC 1 (2.6%); post-UC 2 (5.2%). No significant difference between and within groups. Depressive symptoms (PHQ-9) (6 months) pre-CR 5.0 ± 4.5; post-CR 4.5 ± 5.0; pre-AC 5.4 ± 5.7; post-AC 5.2 ± 5.0; pre-UC 4.4 ± 5.1; post-UC 4.3 ± 4.7. No significant difference between and within groups. Cardiac Knowledge (CADE-Q II) (6 months) pre-CR 51.24 ± 11.9; post-CR 60.8 ± 13.2; pre-AC 48.24 ± 13.3; post-AC 50.1 ± 14.0; pre-UC 45.4 ± 14.8; post-UC 47.6 ± 14.5. Significant difference between CR vs UC (P < 0.01) and CR vs AC groups (*P* < 0.05). No significant difference within groups.
Dehdari 2009,^59^ Iran EMR	*N* = 110; mean age 59.0 years; 28.2% female; 0.0% HF and/or non-ACS patients included	Open, randomised controlled design with 2 parallel arms; 1 site.	UC-No, AC comparison-Y (24 exercise [=3×/wk. for 8 wks]; 40 mins; type of exercise: not specified; other components: 3 education sessions)	ITT: no Anxiety (STAI) (3 months) i) State Anxiety pre-CR 50.70 ± 8.60; post-CR 34.90 ± 1.40; pre-AC 48.60 ± 10.50; post-AC 44.90 ± 4.10 Significant difference between CR and AC groups (P < 0.01) following the intervention. ii) Trait Anxiety pre-CR 49.60 ± 9.10; post-CR 38.00 ± 1.20; pre-AC 48.20 ± 9.20; post-AC 45.30 ± 10.60. Significant difference between CR and AC groups (P < 0.01) following the intervention. Quality of life (SF-36) (3 months) i) Physical functioning pre-CR 59.40 ± 21.20; post-CR 85.60 ± 13.00; pre-AC 54.70 ± 17.80; post-AC 68.70 ± 17.00. Significant difference within groups (*p* < 0.001) and between groups (p < 0.001) following the intervention. ii) Role physical pre-CR 21.30 ± 24.20; post-CR 47.20 ± 30.30; pre-AC 19.0 ± 25.40; post-AC 34.50 ± 30.60. Significant difference within CR group (*p* < 0.05), AC group (p < 0.001) and between groups (p < 0.05) following the intervention. iii) Body pain pre-CR 40.50 ± 22.90; post-CR 71.80 ± 17.70; pre-AC 43.40 ± 24.20; post-AC 55.70 ± 24.20. Significant difference within CR group (p < 0.05), AC group (p < 0.001) and between groups (p < 0.001) following the intervention. iv) General health pre-CR 61.10 ± 18.00; post-CR 78.10 ± 15.20; pre-AC 59.20 ± 17.80; post-AC 62.30 ± 20.20. Significant difference observed only within AC group (p < 0.001) and between groups (p < 0.001) following the intervention. v) Vitality pre-CR 42.50 ± 20.90; post-CR 66.60 ± 18.80; pre-AC 47.10 ± 17.80; post-AC 51.20 ± 20.30. Significant difference observed only within AC group (p < 0.001) and between groups (p < 0.001) following the intervention vi) Social functioning pre-CR 52.50 ± 27.40; post-CR 81.30 ± 22.80; pre-AC 54.50 ± 25.20; post-AC 64.70 ± 28.20. Significant difference within CR group (p < 0.05), AC group (p < 0.001) and between groups (*p* < 0.01) following the intervention. vii) Role emotional pre-CR 27.80 ± 28.50; post-CR 74.50 ± 31.40; pre-AC 29.70 ± 29.10; post-AC 47.20 ± 6.60. Significant difference within CR group (p < 0.05), AC group (p < 0.001) and between groups (p < 0.001) following the intervention. viii) Mental Health pre-CR 51.70 ± 21.40; post-CR 74.50 ± 31.40; pre-AC 56.00 ± 19.70; post-AC 57.80 ± 20.80. Significant difference observed only within AC group (p < 0.001) and between groups (p < 0.001) following the intervention.
Dorje 2019,^29^,^60^ China, WP	*N* = 312; mean age 60.5 years; 18.5% female; 0.0% HF and/or non-ACS patients included	PROBE with 2 parallel arms; 1 site.	UC-Y (The standard of care for Chinese adults after PCI typically involves a brief inpatient health education and ad-hoc follow-up visits with their cardiologists based on their self-assessment of physical health.); AC comparison-No	ITT: y Functional Capacity (6MWD in meter) (6 months) pre-CR 489.2 ± 99.40; post-CR 543.4 ± 67.50; pre-UC 485.00 ± 93.5; post-UC 523.50 ± 60.20. Significant difference between CR and UC groups (P < 0.01) following the intervention. SBP (mmHg) (6 months) pre-CR 124.70 ± 12.80; post-CR 122.50 ± 13.20; pre-UC 123.70 ± 8.80; post-UC 132.00 ± 19.00. No significant difference between and within groups. Total cholesterol (mg/dL) (12 months) pre-CR 150.80 ± 46.40; post-CR 135.30 ± 27.10; pre-UC 146.90 ± 38.70; post-UC 146.90 ± 30.90. No significant difference between and within groups. HDL-C (mg/dL) (12 months) pre-CR 42.50 ± 11.60; post-CR 46.40 ± 11.60; pre-UC 42.50 ± 11.60; post-UC 46.40 ± 11.60; No significant difference between and within groups. LDL-C (mg/dL) (12 months) pre-CR 77.3 ± 38.60; post-CR 69.60 ± 23.20; pre-UC 73.50 ± 30.90; post-UC 77.30 ± 27.10. No significant difference between and within groups. Triglyceride (mg/dL) (12 months) pre-CR 77.30 ± 50.30; post-CR 54.1 ± 30.90; pre-UC 69.60 ± 50.20; post-UC 58.00 ± 30.90. No significant difference between and within groups. Waist-to-hip ratio (%) (6 months) pre-CR 0.90 ± 0.10; post-CR 95.6 ± 11.9; pre-UC 94.9 ± 9.8; post-UC 94.8 ± 9.9. No significant difference between and within groups. Body mass index (kg/m^2^) (6 months) pre-CR 25.50 ± 3.20; post-CR 28.1 ± 4.5; pre-UC 27.8 ± 4.0; post-UC 27.8 ± 3.8. No significant difference between and within groups. Resting HR (bpm) (6 months) pre-CR 68.90 ± 8.20; post-CR 68.90 ± 9.20; pre-UC 74.40 ± 10.30; post-UC 74.40 ± 10.30; Significant difference between groups (*p* = 0.03) following the intervention. Quality of life (SF-36) (6 months) i) PCS pre-CR 43.30 ± 7.40; post-CR 49.53 ± 1.76; pre-UC 35.59 ± 2.12; post-UC 41.01 ± 2.14. Significant difference within groups (*p* < 0.001 for CR and *p* = 0.004 for UC group) and between groups (*p* = 0.002) following the intervention. ii) MCS pre-CR 49.90 ± 9.90; post-CR 47.49 ± 6.01; pre-UC 30.41 ± 9.27; post-UC 35.45 ± 5.70. Significant difference observed only within CR group (*p* < 0.001) and between groups (*p* = 0.003) following the intervention. Tobacco Use (6 months) pre-CR 88(56.41%); post-CR 17(11.41%); pre-UC 89(57.05%); post-UC 9(6.12%). No significant difference between and within groups. Depressive symptoms (PHQ-9) (6 months) pre-CR 4.00 ± 4.30; post-CR 4.5 ± 5.0; pre-UC 4.4 ± 5.1; post-UC 4.3 ± 4.7. No significant difference between and within groups. Anxiety (GAD-7) (6 months) pre-CR 3.50 ± 4.20; post-CR 4.5 ± 5.0; pre-UC 4.4 ± 5.1; post-UC 4.3 ± 4.7. No significant difference between and within groups. CHD Knowledge (Chinese scale scores) (6 months) pre-CR 13.50 ± 5.40; post-CR 60.8 ± 13.2; pre-UC 45.4 ± 14.8; post-UC 47.6 ± 14.5. Significant difference between CR vs UC (*P* < 0.01) and CR vs AC groups (*P* < 0.05). No significant difference within groups. CR/SP needs total (CR Needs Assmt Tool) (6 months) pre-CR 13.50 ± 5.40; post-CR 7.8 ± 7.1; pre-UC 7.9 ± 6.9; post-UC 6.9 ± 5.9. Significant difference between CR vs UC (P < 0.01) and CR vs AC groups (P < 0.01). No significant difference within groups. Adherence to all four cardio-protective medications (12 months) pre-CR 89 (57.05%); post-CR 57 (36.53%); pre-UC 85 (54.48%); post-UC 35 (22.43%). No significant difference between and within groups.
Eraballi 2018; Raghuram 2014,^26^, ^27^, ^28^ India, SEAǁ	*N* = 300; mean age 52.9 years; 0.0% female; 0.0% HF and/or non-ACS patients included	PROBE with 2 parallel arms; 1 site.	UC- No; AC comparison-Y (Three physiotherapy exercise modules developed [pre-operative to 6 weeks, from 6 weeks to 6 months and from 6 months to 12 months] for several body parts in a variety of postures; intensity: NR; type of exercise: breathing exercise; time: NR; other components: dietary sheets)	ITT: y CVD mortality (%) (12 months) CR 1 (0.98%); AC 2 (2.10%); No significant difference between groups. EF (%) (12 months) pre—CR 52.22 ± 6.69; post-CR 55.91 ± 5.21; pre-AC 53.39 ± 7.14; post-AC 54.12 ± 6.84. Significant difference within groups (*p* < 0.001) and no significant difference between groups following the intervention. Total cholesterol (mg/dL) (12 months) pre—CR 151.24 ± 30.35; post-CR 163.04 ± 38.01; pre-AC 154.21 ± 29.92; post-AC 167.43 ± 38.9; Significant difference within CR (*p* = 0.007) and AC (*p* = 0.003) groups and no difference between groups following intervention. HDL-C (mg/dL) (6 months) pre-CR 38.67 ± 6.29; post-CR 40.23 ± 9.3; pre-AC 37.23 ± 7.39; post-AC 37.17 ± 9.68; Significant difference between groups (*p* = 0.003) and no significant difference within groups following intervention. LDL-C (mg/dL) (6 months) pre-CR 75.97 ± 27.65; post-CR 96.61 ± 29.51; pre-AC 78.17 ± 24.15; post-AC 98.77 ± 33.53; Significant difference within CR (*p* = 0.001) and AC (p = 0.001) groups and no difference between groups following intervention. VLDL-C (mg/dL) (6 months) pre-CR 34.92 ± 16.28; post-CR 28.51 ± 12.59; pre-AC 36.21 ± 15.28; post-AC 31.58 ± 13.22; Significant difference within CR (p = 0.001) and AC (*p* = 0.03) groups and between groups (p = 0.03) following intervention. Triglyceride (mg/dL) (6 months) pre-CR 180.19 ± 83.54; post-CR 142.57 ± 62.9; pre-AC 187.13 ± 78.74; post-AC 155.28 ± 57.98; Significant difference within CR (*p* = 0.001) and AC groups (p = 0.001) and between groups following intervention (p = 0.03). Fasting blood glucose (mg/dL) (12 months) pre-CR 122.30 ± 44.13; post-CR 119.50 ± 45.64; pre-AC 121.00 ± 49.61; post-AC 124.02 ± 46.49; Significant difference between pre- and post-CR (*P* = 0.04). Body mass index (kg/m^2^) (12 months) pre-CR 26.76 ± 3.24; post-CR 23.93 ± 2.56; pre-AC 25.22 ± 3.15; post-AC 24.93 ± 3.46; Significant difference within CR group (*p* < 0.001) only and significant difference between CR and AC groups (p < 0.001) following intervention. Depressive symptoms (HADs) 12 months F/U: pre-CR 6.59 ± 3.44; post-CR 4.56 ± 3.51; pre-AC 6.85 ± 3.56; post-AC 5.61 ± 3.3; Significant difference within CR group (p < 0.001) and AC group (*p* = 0.05) following intervention. No significant difference between groups. 5 years F/U: pre-CR 15.65 ± 2.5; post-CR 15.35 ± 2.4; pre-AC 15.39 ± 3.5; post-AC 15.56 ± 2.50; No significant difference between and within groups. Anxiety (HADs) 12 months F/U: pre-CR 7.42 ± 3.40; post-CR 5.75 ± 3.46; pre-AC 7.84 ± 3.05; post-AC 6.15 ± 2.98; Significant difference within CR group (*p* < 0.001) and AC group (p = 0.003) following intervention. No significant difference between groups. 5 years F/U: pre-CR 18.08 ± 2.5; post-CR 18.16 ± 2.90; pre-AC 18.28 ± 2.90; post-AC 18.17 ± 2.50; No significant difference between and within groups. Quality of life (WHO-Bref) Physical Health 12 months F/U: pre-CR 22.30 ± 3.90; post-CR 25.32 ± 3.80; pre-AC 22.84 ± 5.50; post-AC 22.72 ± 4.20. Significant difference within CR group (*p* < 0.001) only and significant difference between groups (p < 0.001) following the intervention. 5 years F/U: pre-CR 23.08 ± 3.90; post-CR 24.89 ± 3.70; pre-AC 23.50 ± 3.00; post-AC 23.56 ± 3.20. Significant difference within CR group (*p* = 0.04) only and no significant difference between groups following the intervention. Mental Health 12 months F/U: pre-CR 19.86 ± 3.70; post-CR 21.23 ± 3.20; pre-AC 19.38 ± 2.80; post-AC 18.61 ± 4.00. Significant difference within CR group (*p* = 0.001) only and significant difference between groups (*p* < 0.001) following the intervention. 5 years F/U: pre-CR 20.22 ± 3.40; post-CR 20.89 ± 3.4; pre-AC 19.61 ± 2.90; post-AC 19.22 ± 2.90. Significant difference between groups (p = 0.05) following the intervention. Social interaction 12 months F/U: pre-CR 10.27 ± 2.50; post-CR 1.88 ± 2.10; pre-AC 10.58 ± 1.80; post-AC 9.43 ± 3.10. Significant difference within CR (*p* = 0.03) and AC group (*p* = 0.002) and significant difference between groups (*p* = 0.01) following the intervention. 5 years F/U: pre-CR 10.57 ± 2.10; post-CR 10.35 ± 1.90; pre-AC 10.44 ± 1.70; post-AC 9.72 ± 2.10. No significant difference within and between groups following the intervention. Environmental health 12 months F/U: pre-CR 25.68 ± 5.10; post-CR 28.31 ± 4.00; pre-AC 25.32 ± 3.60; post-AC 24.48 ± 5.90. Significant difference within CR group (p < 0.001) only and significant difference between groups (p < 0.001) following the intervention. 5 years F/U: pre-CR 25.81 ± 4.30; post-CR 27.19 ± 3.30; pre-AC 25.50 ± 4.40; post-AC 25.69 ± 4.20. Significant improvement within CR groups (p = 0.04) and no difference between groups following the intervention. Total Score 12 months F/U: pre-CR 78.12 ± 13.20; post-CR 85.75 ± 11.20; pre-AC 78.12 ± 10.60; post-AC 75.24 ± 14.90. Significant difference within CR group (p < 0.001) only and significant difference between groups (p < 0.001) following the intervention. 5 years F/U: pre-CR 79.68 ± 12.10; post-CR 83.32 ± 10.80; pre-AC 79.06 ± 9.60; post-AC 78.19 ± 10.60. No significant difference between and within groups 5 years after the intervention. Stress (PSS scale) 12 months F/U: pre-CR 18.76 ± 4.73; post-CR 15.54 ± 4.50; pre-AC 16.28 ± 4.46; post-AC 16.75 ± 4.30. Significant difference within CR group (p < 0.001) only and no significant difference between groups following the intervention. 5 years F/U: pre-CR 19.57 ± 5.00; post-CR 16.59 ± 3.90; pre-AC 16.72 ± 5.30; post-AC 19.03 ± 4.40. Significant difference within CR group(p = 0.001)only and significant difference between groups (p = 0.01) 5 years after the intervention. Positive Affect (PANAS-PA scores) (12 months) pre-CR 39.18 ± 8.16; post-CR 40.54 ± 7.97; pre-AC 34.67 ± 8.72; post-AC 35.83 ± 8.72. Significant difference between groups (*p* = 0.02) and no significant difference within groups following the intervention. Negative Affect (PANAS-NA scores) (12 months) pre-CR 28.57 ± 8.71; post-CR 26.82 ± 8.08; pre-AC 27.00 ± 9.46; post-AC 26.30 ± 7.62. Significant difference within CR groups (*p* = 0.03) only and no significant difference between groups following the intervention.
Farheen/Khalid 2019,^61^^,^^62^ Pakistan, EMR	N = 30; mean age 56.5 years; 38.5% female; 0.0% HF and/or non-ACS patients included	PROBE with 2 parallel arms; 1 site.	UC-No; AC comparison-Y (18 exercise only [=3×/wk. for 6 wks]; aerobic exercise; moderate intensity; 40 mins; other components: no)	ITT: no Functional Capacity (VO_2 Peak_) (6 weeks)¶ pre-CR 11.94; post-CR 15.42; pre-AC 11.27; post-AC 13.23; Significant difference between CR and UC groups (*P* < 0.01) following the intervention. Total cholesterol (mg/dL) (6 weeks) § [median {IQ}] pre-CR [153 {47}]; post-CR [127 {57}]; pre-AC [190 {41}]; post-AC [160 {31}]; No significant difference within groups and significant difference between groups pre-(*p* = 0.007) and post-intervention (p = 0.02). Triglyceride (mg/dL) (6 weeks) pre-CR [169 {151}]; post-CR [116 {122}]; pre-AC [202 {123}]; post-AC [174 {119.5}]; No significant difference between and within groups. EF (%) (6 weeks) pre-CR 45 ± 15; post-CR 55 ± 10; pre-AC 45 ± 10; post-AC 50 ± 5; Significant difference within CR group (p = 0.02) following intervention. QoL (SF-36) (6 weeks) i) Physical functioning pre-CR 41.92 ± 23.32; post-CR 81.92 ± 11.99; pre-AC 49.23 ± 15.11; post-AC 77.69 ± 10.33. No significant difference within and between groups following the intervention. ii) Role physical pre-CR 11.53 ± 29.95; post-CR 98.07 ± 6.93; pre-AC 0.00 ± 0.00; post-AC 84.61 ± 28.20. No significant difference within and between groups following the intervention. iii) Body pain pre-CR 49.61 ± 23.42; post-CR 82.5 ± 13.91; pre-AC 53.26 ± 12.22; post-AC 80.96 ± 16.09. No significant difference within and between groups following the intervention. iv) General health pre-CR 44.61 ± 16.38; post-CR 75 ± 16.58; pre-AC 52.30 ± 23.21; post-AC 73.92 ± 21.54. No significant difference within and between groups following the intervention. v) Energy/Fatigue pre-CR 36.15 ± 11.39; post-CR 71.15 ± 14.01; pre-AC 33.46 ± 15.32; post-AC 54.61 ± 15.33. Significant difference observed only between groups post-intervention (*p* = 0.01) and no significant difference within groups following the intervention vi) Social functioning pre-CR 63.46 ± 21.32; post-CR 92.69 ± 6.07; pre-AC 59.61 ± 16.26; post-AC 80.76 ± 6.49. Significant difference between groups (*p* < 0.001) following the intervention. vii) Role emotional pre-CR 28.20 ± 44.81; post-CR 100.0 ± 0.0; pre-AC 7.69 ± 27.73; post-AC 98.07 ± 6.94. No significant difference within and between groups following the intervention. viii) Mental Health pre-CR 52.61 ± 20.05; post-CR 81.23 ± 11.47; pre-AC 46.15 ± 20.63; post-AC 58.84 ± 20.30. Significant difference observed between groups post-intervention (p < 0.001).
Hasanpour 2020,^42^ Iran, EMR	*N* = 52; mean age 57.7 years; 40.3% female; 100.0% HF and no non-ACS patients included	PROBE with 2 parallel arms;1 site.	UC-Y (No exercise protocol was administered to this group except educational support; All participants received their medications as prescribed by cardiologist), AC comparison-No	ITT: no QoL (SF-36) (6 months) NYHA Class II i) Physical functioning pre-CR 52.56 ± 4.33; post-CR 56.76 ± 4.89; pre-UC 53.35 ± 3.40; post-UC 52.56 ± 5.72. Significant difference within CR (*p* = 0.03) and UC (p = 0.02) groups following the intervention. ii) Role physical pre-CR 51.67 ± 6.83; post-CR 55.66 ± 5.12; pre-UC 52.52 ± 7.34; post-UC 49.32 ± 4.65. Significant difference within CR (*p* = 0.03) and UC group (*p* = 0.04) following the intervention. iii) Body pain pre-CR 67.56 ± 3.46; post-CR 63.44 ± 5.47; pre-UC 66.78 ± 3.24; post-UC 67.35 ± 6.12. Significant difference within CR (p = 0.03) and UC group (*p* = 0.04) following the intervention. iv) General health pre-CR 62.48 ± 11.23; post-CR 66.36 ± 7.89; pre-UC 61.55 ± 9.41; post-UC 57.34 ± 3.74. Significant difference observed within CR (p = 0.001) and UC (*p* = 0.03) groups following the intervention. v) Vitality pre-CR 57.52 ± 5.91; post-CR 63.71 ± 7.67; pre-UC 56.65 ± 4.48; post-UC 51.35 ± 3.66. Significant difference observed within CR (*p* = 0.01) and UC (*p* = 0.03) groups following the intervention vi) Social functioning pre-CR 70.45 ± 7.31; post-CR 74.39 ± 4.54; pre-UC 69.34 ± 4.95; post-UC 66.34 ± 6.45. Significant difference within CR (*p* = 0.03) and UC (*p* = 0.04) groups following the intervention. vii) Role emotional pre-CR 53.41 ± 6.42; post-CR 58.43 ± 8.45; pre-UC 54.76 ± 8.41; post-UC 52.34 ± 3.44. Significant difference within CR (*p* = 0.02) and UC groups (p = 0.04) following the intervention. viii) Mental Health pre-CR 61.22 ± 6.75; post-CR 76.33 ± 5.66; pre-UC 59.98 ± 8.43; post-UC 55.55 ± 5.58. Significant difference observed within CR (*p* = 0.03) and UC (*p* = 0.01) groups following the intervention. ix) Total QoL pre-CR 54.2 ± 8.43; post-CR 57.96 ± 5.65; pre-UC 53.56 ± 6.87; post-UC 50.45 ± 5.34. Significant difference within CR (p = 0.03) and UC (p = 0.03) groups following intervention. NYHA Class III i) Physical functioning pre-CR 48.37 ± 5.20; post-CR 52.34 ± 3.43; pre-UC 47.37 ± 5.42; post-UC 43.42 ± 4.66. Significant difference within CR (p = 0.03) and UC (p = 0.04) groups following the intervention. ii) Role physical pre-CR 49.78 ± 9.61; post-CR 52.32 ± 7.45; pre-UC 50.67 ± 4.76; post-UC 47.34 ± 4.98. Significant difference within CR (p = 0.04) and UC group (p = 0.04) following the intervention. iii) Body pain pre-CR 62.98 ± 8.92; post-CR 58.87 ± 6.99; pre-UC 63.69 ± 6.42; post-UC 67.34 ± 4.29. Significant difference within CR (p = 0.03) and UC group (p = 0.03) following the intervention. iv) General health pre-CR 57.69 ± 8.21; post-CR 61.44 ± 4.35; pre-UC 58.65 ± 10.67; post-UC 53.47 ± 7.34. Significant difference observed within CR (*p* = 0.02) and UC (p = 0.02) groups following the intervention. v) Energy pre-CR 53.89 ± 7.53; post-CR 56.34 ± 8.84; pre-UC 52.78 ± 5.72; post-UC 45.89 ± 4.66. Significant difference observed within CR (p = 0.03) and UC (*p* = 0.01) groups following the intervention vi) Social functioning pre-CR 65.66 ± 9.99; post-CR 68.11 ± 6.76; pre-UC 66.59 ± 8.12; post-UC 60.56 ± 7.34. Significant difference within CR (p = 0.03) and UC groups (p = 0.02) following the intervention. vii) Role emotional pre-CR 52.87 ± 9.48; post-CR 54.98 ± 7.61; pre-UC 52.35 ± 7.34; post-UC 49.44 ± 4.51. Significant difference within CR (p = 0.04) and UC (p = 0.03) groups following the intervention. viii) Mental Health pre-CR 60.79 ± 9.27; post-CR 66.78 ± 7.56; pre-UC 61.57 ± 7.72; post-UC 55.89 ± 5.66. Significant difference observed within CR (p = 0.02) and UC (p = 0.01) groups following the intervention. ix) Total QoL pre-CR 50.98 ± 7.51; post-CR 54.65 ± 6.00; pre-UC 51.76 ± 8.92; post-UC 48.68 ± 6.41. Significant difference within CR (p = 0.02) and UC (p = 0.03) groups following intervention. Fatigue (Visual analog scale) (6 months) NYHA Class II: pre-CR 3.10 ± 1.25; post-CR 2.1 ± 1.21; pre-UC 3.00 ± 1.11; post-UC 6.45 ± 1.40. Significant difference within CR (*p* = 0.001) and UC (p = 0.01) groups following intervention. NYHA Class III: pre-CR 3.90 ± 1.40; post-CR 2.70 ± 1.30; pre-UC 4.10 ± 1.33; post-UC 7.87 ± 2.12.). Significant difference within CR (p = 0.02) and UC (p = 0.01) groups following intervention.
Hassan 2016,^63^ Egypt, EMR	*N* = 60; mean age 53.2 years; 31.7% female; 0.0% HF and/or non-ACS patients included	PROBE with 2 parallel arms; 1 site.	UC- Y (Patients received instruction on risk factors only); AC comparison-No;	ITT: no Functional Capacity (6MWD in meter) (12 months) pre-CR 414.80 ± 57.40; post-CR 489.00 ± 54.80; pre-UC 419.00 ± 50.20; post-UC 430.50 ± 47.30. Significant difference between CR and UC groups (*P* < 0.001) following the intervention. SBP (mmHg) (12 months) pre-CR 129.20 ± 18.70; post-CR 123.80 ± 13.50; pre-UC 128.5 ± 16.6; post-UC 131.2 ± 14.60. Significant difference between groups (*p* < 0.05) following intervention. DBP (mmHg) (12 months) pre-CR 81.3 ± 8.80; post-CR 79.2 ± 7.80; pre-UC 82.80 ± 9.20; post-UC 84.60 ± 8.20. Significant difference between groups (*p* < 0.05) following intervention. Total cholesterol (mg/dL) (12 months) pre-CR 199.1 ± 48.90; post-CR 176.30 ± 42.10; pre-UC 198.80 ± 41.70; post-UC 197.30 ± 39.40; Significant difference between groups (*p* < 0.05) following intervention. HDL-C (mg/dL) (12 months) pre-CR 35.60 ± 8.50; post-CR 37.50 ± 8.80; pre-UC 33.30 ± 7.80; post-UC 32.10 ± 7.40; Significant difference between groups (*p* < 0.05) following intervention. LDL-C (mg/dL) (12 months) pre-CR 134.00 ± 49.10; post-CR 112.10 ± 44.10; pre-UC 135.20 ± 45.30; post-UC 136.00 ± 41.50; Significant difference between groups (*p* < 0.05) following intervention. Triglyceride (mg/dL) (12 months) pre-CR 148.20 ± 34.20; post-CR 132.10 ± 28.80; pre-UC 151.10 ± 32.50; post-UC 149.80 ± 35.10; Significant difference between groups (*p* < 0.05) following intervention. Fasting Blood glucose (mg/dL) (12 months) pre-CR 131.70 ± 47.30; post-CR 106.80 ± 36.50; pre-UC 128.50 ± 54.50; post-UC 127.00 ± 38.30; Significant difference between groups (*p* < 0.05) following intervention. Body mass index (kg/m2) (12 months) pre-CR 30.80 ± 1.90; post-CR 28.20 ± 2.60; pre-UC 30.20 ± 1.70; post-UC 29.60 ± 2.10; Significant difference between groups (*p* < 0.05) following intervention. Tobacco Use (%) (12 months) pre-CR 20 (66.66%); post-CR 15 (50.00%); pre-UC 19 (63.33%); post-UC 14 (50.00%); Significant difference between groups (*p* = 0.007) following intervention and within CR group (*p* < 0.001). Quality of life (SF-36) (12 months) Physical Functioning pre-CR 64.30 ± 7.10; post-CR 83.50 ± 6.50; pre-UC 63.20 ± 6.90; post-UC 76.70 ± 10.60. Significant difference within CR group (p < 0.001) and UC group (p < 0.001) and there is significant difference between groups (*p* < 0.05) following the intervention. Role Physical pre-CR 35.00 ± 24.20; post-CR 62.50 ± 23.40; pre-UC 40.80 ± 23.20; post-UC 50.80 ± 20.20. Significant difference within CR group (p < 0.001) only and significant difference between groups (p < 0.05) following the intervention. Bodily Pain pre-CR 65.20 ± 0.70; post-CR 79.60 ± 18.40; pre-UC 62.70 ± 10.20; post-UC 67.90 ± 15.90. Significant difference within CR (*p* < 0.001) and significant difference between groups (p < 0.05) following the intervention. General health pre-CR 28.20 ± 5.00; post-CR 43.00 ± 7.90; pre-UC 27.30 ± 4.80; post-UC 38.50 ± 8.80. Significant difference within CR group (*p* < 0.001) and UC group (p < 0.001) and there is significant difference between groups (p < 0.05) following the intervention. Role emotional pre-CR 34.1 ± 23.70; post-CR 61.10 ± 21.60; pre-UC 41.80 ± 21.10; post-UC 49.90 ± 19.10. Significant difference within CR group (*p* < 0.001) only and significant difference between groups (p < 0.05) following the intervention. Energy/Fatigue pre-CR 51.70 ± 7.80; post-CR 66.00 ± 11.10; pre-UC 51.30 ± 7.90; post-UC 57.70 ± 11.70; Significant difference within groups-CR(*p* < 0.001), UC(p < 0.05) and significant difference between groups (p < 0.05) following the intervention. Emotional wellbeing pre-CR 61.3 ± 6.20; post-CR 69.50 ± 2.60; pre-UC 59.10 ± 6.10; post-UC 61.50 ± 7.50; Significant difference within groups-CR(p < 0.001), UC(p < 0.001) and significant difference between groups (p < 0.05) following the intervention. Social functioning pre-CR 50.90 ± 10.50; post-CR 67.50 ± 19.00; pre-UC 51.70 ± 10.90; post-UC 67.90 ± 15.90; Significant difference within CR group (p < 0.001) only and significant difference between groups (p < 0.05) following the intervention.
Haq 2019,^64^ Pakistan EMR	*N* = 206; mean age 53.6 years; 23.08% female; 0.0% HF and/or non-ACS patients included	PROBE with 2 parallel arms; 1 site.	UC- Y (standard of care by cardiologist includes brief counselling about patients' health condition, medication and follow-up advice); AC comparison-No	ITT: y All-cause mortality (%) (2 months) CR 3 (2.91%); UC 5 (4.85%); No significant difference between groups. Quality of life (MacNew QLMI) (2 months) pre-CR 3.60 ± 1.07; post-CR 5.6 ± 0.50; pre-UC 3.90 ± 0.50; post-UC 3.80 ± 0.50; Significant difference within CR (p < 0.001) and UC (*p* = 0.01) groups following intervention. There is significant difference between groups (p < 0.001). Self-rated Health (measured by a single question on physical health with Likert scale scores from ‘excellent’ to ‘poor’) (2 months) pre-CR 3.97 ± 0.90; post-CR 2.30 ± 0.80; pre-UC 3.90 ± 0.07; post-UC 4.06 ± 0.06; Significant difference within CR (p < 0.001) and UC (*p* = 0.04) groups following intervention. There is significant difference between groups (p < 0.001). Psychological Well-Being (General Health Questionnaire) (2 months) pre-CR 21.20 ± 5.50; post-CR 7.40 ± 4.20; pre-UC 18.71 ± 4.30; post-UC 20.90 ± 5.20; Significant difference within CR (p < 0.001) and UC (p < 0.001) groups following intervention. There is significant difference between groups (p < 0.001).
Jena 2020,^32^ India, SEA	*N* = 40; mean age not reported; 37.5% female; 100.0% HF and no non-ACS patients included	Randomised with 2 parallel arms; 2 site.	UC- Y (The standard of care for HF in India include patients are under regular follow-up of physicians and cardiologists as deemed medically appropriate); AC comparison-No	ITT: no Functional Capacity (VO_2_ max) (1 month)∫ post-CR 17.42 ± 3.86; post-UC 14.07 ± 6.18. Significant difference between CR and UC groups (P = 0.02) following the intervention. Anxiety (Hamilton scale) (1 month) ∫ post-CR 7.05 ± 3.67; post-UC 15.80 ± 3.54; Significant difference between CR and UC group (p < 0.00001) following intervention. HF symptoms (scores of a self-structured four-point rating scale) (1 month): post-CR 9.75 ± 1.51; post-UC 15.50 ± 3.96; Significant difference between groups (*p* < 0.00001). Pain (scores from a numeric pain rating scale) (1 month) post-CR 1.2 ± 0.40; post-UC 4.8 ± 1.45; Significant difference between groups (p < 0.00001). Oedema (scores of oedema grading scale) (1 month): post-CR 1.4 ± 1.44; post-UC 0.54 ± 0.99; Significant difference between groups (p = 0.01).
Lima 2020, ^46^^,^^65^ Brazil, AMR	*N* = 49; mean age 56.5 years; 14.3% female; 0.0% HF and/or non-ACS patients included	PROBE with 2 parallel arms; 1 site.	UC- No; AC comparison-Y (60 [5×/wk. for 12 wks = 24 supervised +36 unsupervised] aerobic exercise; moderate intensity; 40 mins; other components: 24 pt. education sessions)	ITT: no Hospitalization (%) (3 months) CR 4 (21.05%); AC 1 (4.76%); No significant difference between groups. Adverse events (%) (3 months) CR 11 (57.89%); AC 7 (33.33%); No significant difference between groups. Functional Capacity (ISWD, meters) (6 months) pre-CR 422.61 ± 57.40; post-CR 452.17 ± 119.80; pre-AC 417.22 ± 122.70; post-AC 466.11 ± 119.80. Significant difference within CR group (*p* = 0.006) following the intervention. Functional Capacity (DASI) (6 months) pre-CR 41.12 ± 11.20; post-CR 45.96 ± 12.40; pre-AC 37.43 ± 11.20; post-AC 42.19 ± 12.40. Significant difference between CR and AC groups (*P* < 0.001) following the intervention. SBP (mmHg) (6 months) pre-CR 113.67 ± 16.10; post-CR 118.71 ± 21.90; pre-AC 108.42 ± 16.10; post-AC 105.26 ± 21.90. No significant difference between and within groups following intervention. DBP (mmHg) (6 months) pre-CR 69.58 ± 8.70; post-CR69.96 ± 8.20; pre-AC 69.47 ± 8.70; post-AC 65.26 ± 8.20. No significant difference between and within groups following intervention. Total cholesterol (mg/dL) (6 months) pre-CR 155.37 ± 56.30; post-CR 145.84 ± 33.30; pre-AC 160.73 ± 56.30; post-AC 139.93 ± 30.40; No significant difference between and within groups following intervention. Fasting blood glucose (mg/dL) (6 months) pre-CR 119.00 ± 34.19; post-CR 105.31 ± 19.80; pre-AC 106.86 ± 34.10; post-AC 113.21 ± 19.80; No significant difference between and within groups following intervention. HbA1_c_ (%) (6 months) pre-CR 6.59 ± 1.20; post-CR 6.33 ± 1.00; pre-AC 6.25 ± 1.20; post-AC 6.33 ± 1.00. No significant difference between and within groups following intervention. Waist circumference (cm) (6 months) pre-CR 100.42 ± 9.70; post-CR 99.71 ± 10.80; pre-AC 97.95 ± 9.80; post-AC 98.76 ± 10.80; No significant difference between and within groups following intervention. Quality of life (SF-36) (6 months) PCS pre-CR 70.35 ± 20.10; post-CR 73.17 ± 20.50; pre-AC 62.67 ± 20.10; post-AC 68.29 ± 20.40; Significant difference within CR (*p* < 0.001) groups and no significant difference between groups following intervention. MCS pre-CR 76.65 ± 17.70; post-CR 80.39 ± 16.80; pre-AC 72.62 ± 17.70; post-AC 79.43 ± 16.80. Significant difference within CR (p < 0.001) groups and no significant difference between groups following intervention. Depressive symptoms (PHQ-9) (6 months) pre-CR 3.38 ± 3.50; post-CR 3.21 ± 0.21; pre-AC 2.81 ± 3.60; post-AC 2.14 ± 0.22; Significant difference within CR group (*p* = 0.01) following intervention. No significant difference between groups. Cardiovascular knowledge (CADEQ-SVs) (6 months) pre-CR 13.96 ± 2.10; post-CR 15.25 ± 1.80; pre-AC 13.76 ± 2.00; post-AC 15.86 ± 1.80; Significant difference between groups (*p* = 0.02) and no significant difference within groups following the intervention. Intervention cost (Per patient in Brazilian Real) (6 months) post-CR 552.73; post-UC 242.72.
Mehani 2018,^40^ Egypt, EMR	*N* = 45; mean age 48.7 years; 0.0% female; 0.0% HF and/or non-ACS patients included	PROBE with 2 parallel arms; 1 site.	UC- Y (advised to continue medications and ordinary physical activities); AC comparison-No	ITT: no Functional Capacity (VO_2Peak_ ml/Kg/min) (3 months) pre-CR 19.24 ± 0.79; post-CR 23.18 ± 1.25; pre-UC 19.47 ± 0.71; post-UC 19.66 ± 1.30; Significant difference within CR groups (*p* = 0.0001) only and significant difference between groups (p = 0.0001) following the intervention. Muscle strength (Kg) (3 months) Quadriceps force pre-CR 6.32 ± 0.67; post-CR 9.50 ± 0.93; pre-UC 6.45 ± 0.58; post-UC 6.53 ± 0.72; Significant difference within CR groups (p = 0.0001) only and significant difference between groups (p = 0.0001) following the intervention. Biceps Brachii force pre-CR 4.82 ± 0.67; post-CR 6.39 ± 0.83; pre-UC 4.97 ± 0.61; post-UC 5.07 ± 0.71; Significant difference within CR groups (p = 0.0001) only and significant difference between groups (p = 0.0001) following the intervention. Homocysteine (micromole/L) (3 months) pre-CR 20.48 ± 3.69; post-CR 14.82 ± 3.40; pre-UC 19.60 ± 2.98; post-UC 20.01 ± 3.07; Significant difference within CR groups (p = 0.0001) only and significant difference between groups (p = 0.0001) following the intervention. Apolipoprotein A1b (mg/L) (12 months) pre-CR 1.01 ± 0.13; post-CR 1.65 ± 0.29; pre-UC 0.95 ± 0.14; post-UC 0.85 ± 0.27; Significant difference within CR groups (p = 0.0001) only and significant difference between groups (p = 0.0001) following the intervention.
Mehani 2013,^39^ Egypt, EMR	N = 40; mean age 55.5 years; 0.0% female; 0.0% HF and/or non-ACS patients included	PROBE with 2 parallel arms; 1 site.	UC- Y (Patients received simple disease information and biweekly physician/cardiologist consultation); AC comparison- No	ITT: y Adverse events (%) (7 months) CR 3 (20.00%); UC 0 (0.00%); No significant difference between groups. Functional Capacity (VO_2Peak_ ml/Kg/min) (7 months) pre-CR 16.10 ± 3.65; post-CR 21.08 ± 5.47; pre-UC 17.17 ± 2.44; post-UC 17.48 ± 2.24. Significant difference within CR groups (*p* = 0.01) only and significant difference between groups (*p* = 0.02) following the intervention. Resting HR (bpm) (7 months) pre-CR 93.60 ± 7.43; post-CR 75.00 ± 8.01; pre-UC 87.47 ± 12.88; post-UC 87.33 ± 7.99; Significant difference within CR (p = 0.01) groups and significant difference between groups (*p* = 0.004) following intervention. Maximal HR (bpm) (7 months) pre-CR 141.00 ± 12.41; post-CR 126.80 ± 12.34; pre-UC 133.93 ± 20.32; post-UC 134.07 ± 14.25; Significant difference within CR groups (*p* = 0.006) and no significant difference between groups following intervention. EF (%) (7 months) pre-CR 33.09 ± 4.77; post-CR 48.93 ± 8.38; pre-UC 35.80 ± 6.87; post-UC 37.27 ± 7.82. Significant difference within CR groups (*p* = 0.001) and between groups (p = 0.001) following intervention. e/a ratio type (7 months) Normal diastolic pattern pre-CR 0 (0.00%); post-CR 8 (53.30%); pre-UC 0 (0.00%); post-UC 0 (0.00%); Significant difference within CR (p = 0.01) groups and between groups (*p* = 0.009) following intervention. Grade I diastolic dysfunction pre-CR 11 (73.4%); post-CR 1 (6.70%); pre-UC 7 (46.60%); post-UC 8 (53.30%); Significant difference within CR (p = 0.01) groups and between groups (p = 0.009) following intervention. Grade II diastolic dysfunction pre-CR 2 (13.30%); post-CR 1 (6.70); pre-UC 4 (22.70%); post-UC 3 (20.00%); Significant difference within CR (p = 0.01) groups and between groups (p = 0.009) following intervention. Grade III diastolic dysfunction pre-CR 2 (13.30%); post-CR 5 (33.33); pre-UC 4 (22.70%); post-UC 4 (22.70%); Significant difference within CR (p = 0.01) groups and between groups (p = 0.009) following intervention. QoL (KCCQ) (7 months) Clinical summary scores Median change scores in CR 129.28; Median change in UC 7.04; Significant difference between CR and UC groups (*P* = 0.0001). Functional summary scores Median change scores in CR 75.01; Median change in UC 10.85; Significant difference between CR and UC groups (*P* = 0.0004).
Moeini 2015,^66^ Iran, EMR	N = 40; mean age 60.15 years; 29.6% female; % of HF and /or non-ACS patients included- NR	PROBE with 2 parallel arms; 1 site.	UC- No; AC comparison-Y (16 [=2×/wk. for 8 wks]; aerobic exercise; low intensity; 15 mins; other components: dietary education and psychosocial counselling)	ITT: no SBP (mmHg) (8 weeks) pre-CR 128.21 ± 15.39; post-CR 116.42 ± 7.18; pre-AC 120.00 ± 20.51; post-AC 112.00 ± 12.60. Significant difference within CR group (*p* = 0.02) following intervention. DBP (mmHg) (8 weeks) pre-CR 82.50 ± 9.35; post-CR 81.78 ± 7.99; pre-AC 82.00 ± 13.11; post-AC 87.25 ± 12.48. No significant difference between and within groups following intervention.
Passaglia, 2020,^41^^,^^67^ Brazil, AMR	*N* = 180; mean age 58.0 years; 25.6% female; 0.0% HF and/or non-ACS patients included	PROBE with 2 parallel arms; 1 site.	UC- No; AC comparison-Y (supervised exercise for 3 consecutive months; exercise was not specified as per FITT; other components: NR); 1 site;	ITT: y All-cause Hospitalization (%) (6 months) CR 15 (19.48%); AC 24 (32.87%); No significant difference between groups. SBP (mmHg) (6 months) pre—CR 115.40 ± 16.90; post-CR 121.50 ± 19.50; pre-AC 112.10 ± 15.20; post-AC 120.50 ± 15.40; Significant difference within CR (p = 0.007) and AC (p = 0.003) groups and no difference between groups following intervention. DBP (mmHg) (6 months) pre-CR 70.80 ± 10.70; post-CR 73.80 ± 12.60; pre-AC 69.30 ± 10.30; post-AC 73.80 ± 10.30; Significant difference within CR (*p* = 0.007) and AC (*p* = 0.003) groups and no difference between groups following intervention. Total cholesterol (mg/dL) (6 months) pre-CR 173.50 ± 43.20; post-CR 155.70 ± 43.90; pre-AC 170.60 ± 40.60; post-AC 157.20 ± 36.9; Significant difference within CR (p = 0.007) and AC (p = 0.003) groups and no difference between groups following intervention. HDL-C (mg/dL) (6 months) pre-CR 41.80 ± 10.60; post-CR 40.00 ± 10.20; pre-AC 43.20 ± 12.40; post-AC 43.00 ± 11.40; Significant difference between groups (p = 0.003) and no significant difference within groups following intervention. LDL-C (mg/dL) (6 months) pre-CR 103.70 ± 41.40; post-CR 82.80 ± 36.80; pre-AC 97.70 ± 38.40; post-AC 81.60 ± 31.90; Significant difference within CR (*p* = 0.001) and AC (p = 0.001) groups and no difference between groups following intervention. Triglyceride (mg/dL) (6 months) pre-CR 143.80 ± 71.40; post-CR 162.80 ± 66.90; pre-AC 142.80 ± 73.70; post-AC 164.30 ± 75.80; Significant difference within CR (p = 0.001) and AC groups (p = 0.001) and between groups following intervention (*p* = 0.03). Body mass index (kg/m^2^) (6 months) pre-CR 28.20 ± 5.30; post-CR 28.60 ± 5.20; pre-AC 28.70 ± 4.60; post-AC 29.00 ± 4.60; Significant difference within CR group (*p* < 0.001) only and significant difference between CR and AC groups (p < 0.001) following intervention. HR (bpm) (6 months) pre-CR 122.30 ± 44.13; post-CR 119.50 ± 45.64; pre-AC 121.00 ± 49.61; post-AC 124.02 ± 46.49; Significant difference between pre- and post-CR (*P* = 0.04). Tobacco Use (6 months) pre-CR 610 (31.00%); post-CR 449 (22.99%); pre-AC 592 (29.80%); post-AC 445 (22.61%); No significant difference between and within groups. Physical activity (150 mins/wk.; IPAQ) (6 months) pre-CR 610 (31.00%); post-CR 449 (22.99%); pre-AC 592 (29.80%); post-AC 445 (22.61%); No significant difference between and within groups. Medication adherence (Treatment adherence measure) (6 months) pre-CR 610 (31.00%); post-CR 449 (22.99%); pre-AC 592 (29.80%); post-AC 445 (22.61%); No significant difference between and within groups. Risk factor control (achieving score 4 or 5 points) (6 months) pre-CR 610 (31.00%); post-CR 449 (22.99%); pre-AC 592 (29.80%); post-AC 445 (22.61%); No significant difference between and within groups. Health Literacy (SAHLPA-18) (6 months) pre-CR 610 (31.00%); post-CR 449 (22.99%); pre-AC 592 (29.80%); post-AC 445 (22.61%); No significant difference between and within groups.
Salvetti 2008,^45^ Brazil, AMR	N = 39; mean age 53.5 years; 25.6% female; 0.0% HF and/or non-ACS patients included	PROBE with 2 parallel arms; 1 site.	UC- Y (The standard of care in Brazil include physician's advice to improve physical activity and medication adherence under routine follow-up; AC comparison-No	ITT: y Functional Capacity (Peak VO_2_) (mL/Kg/min) (3 months) pre-CR 28.80 ± 6.40; post-CR 31.70 ± 8.10; pre-UC 28.60 ± 6.60; post-UC 26.80 ± 7.20. There is significant difference within CR group (*p* < 0.05) and UC group (p < 0.05) following the intervention. Resting SBP (mmHg) (3 months) pre-CR 133.00 ± 15.00; post-CR 125.00 ± 12.00; pre-UC 132.00 ± 15.00; post-UC 134.00 ± 16.00; Significant difference within CR (p < 0.05) group following intervention. Resting DBP (mmHg) (3 months) pre-CR 85.00 ± 7.00; post-CR 84.00 ± 6.00; pre-UC 89.00 ± 7.00; post-UC 87.00 ± 7.00; No significant difference within groups following intervention. Peak SBP (mmHg) (3 months) pre-CR 185.00 ± 17.00; post-CR 178.00 ± 15.00; pre-UC 185.00 ± 21.00; post-UC 184.00 ± 25.00; No significant difference within groups following intervention. Peak DBP (mmHg) (3 months) pre-CR 91.00 ± 9.00; post-CR 85.00 ± 5.00; pre-UC 89.00 ± 9.00; post-UC 90.00 ± 7.00; No significant difference within groups following intervention. Peak HR (bpm) (3 months) pre-CR 135.00 ± 22.00; post-CR 143.00 ± 20.00; pre-UC 138.00 ± 11.00; post-UC 134.00 ± 17.00; Significant difference within CR (*p* < 0.05) group following intervention. Quality of life (SF-36) (3 months) Physical Functioning pre-CR 85.00 ± 9.86; post-CR 97.32 ± 2.63; pre-UC 80.50 ± 14.04; post-UC 78.00 ± 23.81. Significant difference within CR and UC group (*p* < 0.001) following the intervention. Role Physical pre-CR 44.00 ± 32.25; post-CR 93.11 ± 16.76; pre-UC 62.50 ± 39.32; post-UC 61.20 ± 34.86. Significant difference within CR and UC group (p < 0.001) following the intervention. Bodily Pain pre-CR 71.21 ± 18.92; post-CR 97.68 ± 7.22; pre-UC 72.25 ± 23.47; post-UC 64.80 ± 17.22. Significant difference within CR and UC group (p < 0.001) following the intervention. General health pre-CR 65.84 ± 20.40; post-CR 82.63 ± 18.19; pre-UC 75.95 ± 18.13; post-UC 67.65 ± 14.27. Significant difference within CR and UC group (p < 0.001) following the intervention. Role emotional pre-CR 47.40 ± 42.06; post-CR 94.74 ± 12.49; pre-UC 53.33 ± 41.04; post-UC 60.00 ± 33.51. Significant difference within CR and UC group (p < 0.001) following the intervention. Vitality pre-CR 62.37 ± 13.68; post-CR 77.11 ± 10.71; pre-UC 67.00 ± 13.61; post-UC 57.65 ± 12.76. Significant difference within CR and UC group (p < 0.001) following the intervention. Mental health pre-CR 55.26 ± 17.27; post-CR 71.79 ± 16.03; pre-UC 55.00 ± 16.31; post-UC 64.30 ± 13.11. Significant difference within CR and UC group (p < 0.001) following the intervention. Social functioning pre-CR 78.29 ± 23.51; post-CR 98.03 ± 6.27; pre-UC 80.00 ± 23.08; post-UC 81.25 ± 20.48. Significant difference within CR and UC group (*p* < 0.001) following the intervention. HR reserve (%) (3 months) pre-CR 82.00 ± 13.00; post-CR 87.00 ± 12.00; pre-UC 85.00 ± 8.00; post-UC 82.00 ± 11.00; Significant difference within CR (*p* < 0.05) group following intervention. Rate pressure product (bpm.mm Hg) (3 months) pre-CR 25113.00 ± 5163.00; post-CR 25543.00 ± 4774.00; pre-UC 25624.00 ± 3920.00; post-UC 26240.00 ± 10,099; There is no significant difference within groups following intervention. Ventilatory threshold (mL/Kg/min) (3 months) pre-CR 21.80 ± 4.80; post-CR 22.80 ± 4.40; pre-UC 21.80 ± 4.70; post-UC 21.20 ± 4.10; There is no significant difference within groups following intervention. Peak O_2_ Pulse (mL/bpm) (3 months) pre-CR 15.30 ± 3.40; post-CR 15.70 ± 4.00; pre-UC 15.50 ± 3.90; post-UC 14.30 ± 3.80; Significant difference within UC group (p < 0.05) following intervention. Peak expiratory exchange ratio (3 months) pre-CR 1.15 ± 0.11; post-CR 1.19 ± 0.08; pre-UC 1.10 ± 0.08; post-UC 1.12 ± 0.11; There is no significant difference within groups following intervention. Work rate (kpm/min) (3 months) pre-CR 4780.00 ± 2021.00; post-CR 7103.00 ± 3057.00; pre-UC 5507.00 ± 2498.00; post-UC 5747.00 ± 3085.00; Significant difference within CR (p < 0.05) group following intervention. Treadmill exercise time (min) (3 months) pre-CR 11.50 ± 1.90; post-CR 13.60 ± 2.30; pre-UC 11.50 ± 2.30; post-UC 11.40 ± 2.70; Significant difference within CR (p < 0.05) and UC (p < 0.05) groups following intervention.
Suleimani 2018,^68^ Iran, EMR	*N* = 101; mean age 51.0 years; 30.6% female; 0.0% HF and/or non-ACS patients included	PROBE with 2 parallel arms; 1 site.	UC- Y (this includes usual care routines for MI at cardiac intensive care unit of hospitals); AC comparison-No	ITT: y All-cause Hospitalization (%) (2 months) CR 2 (4.00%); UC 0 (0.00%); No significant difference between groups. Adherence to treatment (assessed using specific questionnaire designed by Kamrani F et al.; higher scores better) (2 months) pre-CR 103.44; post-CR 133.73; pre-UC 105.08; post-UC 103.92; Significant difference within CR (*p* < 0.001) groups and between groups (p < 0.001) 2 months post-intervention. Dietary adherence (diet part of specific questionnaire designed by Kamrani F et al.) (2 months) pre-CR 79.60 ± 13.31; post-CR 100.28 ± 9.66; pre-UC 81.92 ± 14.72; post-UC 82.42 ± 14.90; Significant difference within CR (*p* < 0.001) group following intervention. Medication adherence (medication part of specific questionnaire designed by Kamrani F et al.) (2 months) pre-CR 4.67 ± 2.20; post-CR 6.45 ± 1.43; pre-UC 4.82 ± 2.60; post-UC 4.03 ± 2.22; Significant difference within CR and UC (*p* < 0.001) groups following intervention. Physical activity (exercise part of specific questionnaire designed by Kamrani F et al.) (2 months) pre-CR 16.58 ± 5.10; post-CR 26.00 ± 3.59; pre-UC 17.20 ± 4.34; post-UC 16.94 ± 4.84; Significant difference within CR (*p* < 0.001) group following intervention.
Uddin 2019,^69^ Bangladesh, SEA	*N* = 142; mean age 54.0 years; 7.0% female; 0.0% HF and/or non-ACS patients included	Quasi-randomised controlled trial with 2 parallel arms; 1 site.	UC- Y (The standard of care for cardiovascular patients in Bangladesh includes conventional hospital discharge care along with medication adjustment and routine follow-up advices by cardiologists); AC comparison-No	ITT: y Functional Capacity (VO_2 max_) (mL/Kg/min) (6 months) ∫ post-CR 35.70 ± 10.12; post-UC 29.13 ± 12.95; Significant difference between groups (*p* < 0.01) post-intervention. SBP (mmHg) (6 months) pre-CR 122.95 ± 13.02; post-CR 114.42 ± 6.71; pre-UC 126.5 ± 15.56; post-UC 116.87 ± 10.23; Significant difference within CR (p < 0.001) and UC (*p* = 0.001) groups and no difference between groups following intervention. DBP (mmHg) (6 months) pre-CR 77.45 ± 8.38; post-CR 74.01 ± 5.68; pre-UC 79.75 ± 7.64; post-UC 76.62 ± 6.34; Significant difference within CR (*p* = 0.009) and UC (*p* = 0.05) groups and no difference between groups following intervention. Total cholesterol (mg/dL) (6 months) pre-CR 179.98 ± 53.17; post-CR 112.29 ± 36.40; pre-UC 175.27 ± 55.96; post-UC 160.57 ± 53.35; Significant difference within CR (p < 0.001) group and significant difference between groups (p < 0.001) following intervention. HDL-C (mg/dL) (6 months) pre-CR 30.80 ± 6.50; post-CR 35.34 ± 4.79; pre-UC 29.40 ± 6.53; post-UC 30.80 ± 4.94; Significant difference within CR (p < 0.001) group and significant difference between groups (*p* = 0.01) following intervention. LDL-C (mg/dL) (6 months) pre-CR 131.44 ± 54.46; post-CR 87.14 ± 23.17; pre-UC 123.14 ± 56.38; post-UC 119.05 ± 40.31; Significant difference within CR (p < 0.001) group and significant difference between groups (p < 0.001) following intervention. Triglyceride (mg/dL) (6 months) pre-CR 180.42 ± 114.36; post-CR 108.85 ± 48.83; pre-UC 179.92 ± 75.94; post-UC 161.37 ± 64.72; Significant difference within CR (p < 0.001) group and significant difference between groups (*p* = 0.002) following intervention. Body mass index (kg/m^2^) (6 months) pre-CR 25.54 ± 2.53; post-CR 24.63 ± 2.26; pre-UC 24.77 ± 2.86; post-UC 24.47 ± 2.68; Significant difference within CR group (*p* = 0.03) only and significant difference between CR and UC groups (*p* = 0.008) following intervention. Depressive symptoms (PHQ-9) (3 months) pre-CR 12.90 ± 1.83; post-CR 4.60 ± 1.93; pre-UC 14.40 ± 2.46; post-UC 9.67 ± 1.83; Significant difference within CR group (*p* < 0.01) and UC group (p < 0.01) following intervention. There is significant difference between groups (p < 0.01). Quality of life (WHO-Bref) (12 months) Overall QoL pre-CR 2.96 ± 0.49; post-CR 4.03 ± 0.49; pre-UC 2.85 ± 0.43; post-UC 3.20 ± 0.82. Significant difference within CR group (p < 0.01) and UC (*p* < 0.02). There is significant difference between groups (p < 0.01) following the intervention. Overall perception of health pre-CR 2.59 ± 0.67; post-CR 4.06 ± 0.40; pre-UC 2.47 ± 0.55; post-UC 3.17 ± 0.38. Significant difference within CR group (p < 0.01) and UC (p < 0.01). There is significant difference between groups (p < 0.01) following the intervention. Physical domain pre-CR 20.81 ± 2.47; post-CR 26.90 ± 2.88; pre-UC 21.67 ± 1.76; post-UC 21.17 ± 3.35. Significant difference within CR group (p < 0.01). There is significant difference between groups (p < 0.01) following the intervention. Psychological domain pre-CR 17.67 ± 2.00; post-CR 23.42 ± 2.84; pre-UC 18.10 ± 1.70; post-UC 17.87 ± 3.19. Significant difference within CR group (p < 0.01) only and significant difference between groups (p < 0.01) following the intervention. Social relationship domain pre-CR 10.13 ± 1.55; post-CR 11.83 ± 1.25; pre-UC 12.05 ± 1.35; post-UC 10.75 ± 0.89. Significant difference within CR group (p < 0.01) and UC (p < 0.01). There is significant difference between groups (p < 0.01) following the intervention. Environmental domain pre-CR 23.57 ± 2.91; post-CR 28.80 ± 4.24; pre-UC 20.25 ± 5.48; post-UC 21.77 ± 5.31. Significant difference within CR (p < 0.01) and significant difference between groups (p = 0.03) following the intervention. HbA1_c_ (mmol/L) (6 months) pre-CR 7.00 ± 2.00; post-CR 6.28 ± 0.92; pre-UC 7.00 ± 1.50; post-UC 6.42 ± 1.03; Significant difference within CR (*p* < 0.001) and UC (*P* = 0.019). There is no significant difference between groups.
Venkatesh 2019,^70^ India, SEA	*N* = 40; mean age 58.5 years; 8.0% female; % HF and/or non-ACS patients included- NR	PROBE with 2 parallel arms; 1 site.	UC- No; AC comparison-Y (exercise counselling through telephonic guidance once a week for 12 weeks);	ITT: y All-cause mortality (%) (3 months) CR 1 (5.00%); AC 1 (5.00%); No significant difference between groups. Functional Capacity (6MWD, metres) (3 months) pre-CR 201.15 ± 73.20; post-CR 490.00 ± 50.62; pre-AC 212.91 ± 63.85; post-AC 416.00 ± 49.78. There is significant difference within CR group (*p* ≤ 0.001) following the intervention. There is significant difference between CR and AC groups (p ≤ 0.001) post-intervention. RRIV ANS dysfunction Resting 825.00 ± 121.80; Hyperventilation 772.72 ± 60.71; Post 2 min 791.38 ± 76.97; Post 5 min 810.90 ± 51.29; Post 10 min 802.50 ± 29.98; pre-6MWD 213.40 ± 59.68; post-6MWD 413.60 ± 62.42;
Yadav 2015,^71^ India, SEA	*N* = 80; mean age 55.8 years; % female-NR; % HF and/or non-ACS patients included-NR	PROBE with 2 parallel arms; 1 site.	UC- Y (consisted of conventional medical treatment); AC comparison-No	ITT: y SBP (mmHg) (3 months) pre-CR 142.80 ± 12.80; post-CR 126.60 ± 14.20; pre-UC 142.60 ± 8.80; post-UC 132.63 ± 6.40; There is significant difference within CR (*p* ≤ 0.05) group following intervention. DBP (mmHg) (3 months) pre-CR 84.40 ± 10.20; post-CR 80.00 ± 8.40; pre-UC 82.53 ± 4.60; post-UC 80.20 ± 5.40; There is significant difference within CR (p ≤ 0.05) group following intervention. MBP (mmHg) (3 months) pre-CR 104.46 ± 10.60; post-CR 96.29 ± 8.28; pre-UC 103.56 ± 12.25; post-UC 97.81 ± 11.57; No significant difference within groups following intervention. HR (bpm) (3 months) pre-CR 88.50 ± 9.22; post-CR 76.20 ± 8.51; pre-UC 87.80 ± 8.54; post-UC 83.60 ± 9.88; There is significant difference within CR (p ≤ 0.05) group following intervention. Pulmonary Function Tests SVC (L) (3 months) pre-CR 1.87 ± 0.09; post-CR 2.19 ± 0.10; pre-UC 1.45 ± 0.08; post-UC 1.43 ± 0.05; There is significant difference within CR group (*p* = 0.002) following intervention and also between groups (*p* < 0.001). FVC (L) (3 months) pre-CR 1.59 ± 0.08; post-CR 2.12 ± 0.12; pre-UC 1.59 ± 0.10; post-UC 1.57 ± 0.08; Significant difference within CR (p = 0.002) group following intervention and significant difference between groups (*p* = 0.001). FEV1 (L) (3 months) pre-CR 1.44 ± 0.09; post-CR 1.74 ± 0.09; pre-UC 1.23 ± 0.09; post-UC 1.26 ± 0.08; No significant difference within groups and significant difference between groups following intervention (*p* = 0.04). FEV1% (%) (3 months) pre-CR 85.44 ± 1.90; post-CR 82.82 ± 2.38; pre-UC 80.19 ± 2.33; post-UC 77.35 ± 2.60; No significant difference within and between groups following intervention. PEFR (L/s) (3 months) pre-CR 3.32 ± 0.27; post-CR 4.08 ± 0.25; pre-UC 2.81 ± 0.24; post-UC 2.73 ± 0.19; There is significant difference within CR group (*p* = 0.05) following intervention and significant difference between groups (p < 0.001). MVV (L/min) (3 months) pre-CR 46.71 ± 2.83; post-CR 59.67 ± 3.29; pre-UC44.76 ± 3.49; post-UC 43.96 ± 3.42; There is significant difference between (*p* = 0.014) and within CR (*p* = 0.02) group following intervention. DLCO (ml/min/mm of Hg) (3 months) pre-CR 13.80 ± 0.66; post-CR17.50 ± 0.88; pre-UC 12.91 ± 1.08; post-UC 13.05 ± 1.16; There is significant difference within CR group (p = 0.01) and between groups (*p* = 0.03) following intervention.
Zhang 2017,^34^ China, WP	*N* = 126; mean age 63.7 years; 29.5% female; 0.0% HF and/or non-ACS patients included	PROBE with 2 parallel arms; 2 sites.	UC- Y (The usual care in China involves routine management of chronic diseases by community physicians and nurses; AC comparison-No	ITT: y Functional Capacity (6MWD in meter) (6 months) pre-CR 501.20 ± 73.40; post-CR 558.62 ± 155.08; pre-UC 511.80 ± 80.40; post-UC 502.00 ± 200.59. Significant difference within CR group (95% CI of change 41.60, 73.20) following the intervention. There is significant difference between CR and UC groups (*p* < 0.01). Body mass index (kg/m^2^) (6 months) pre-CR 26.20 ± 4.20; post-CR 25.80 ± 13.09; pre-UC 25.90 ± 3.70; post-UC 25.70 ± 4.60; Significant difference within CR group (95% CI of change −3.8, 3.0) and no significant difference between CR and UC groups. Quality of life (SF-12 v2) (6 months) PCS pre-CR 38.70 ± 8.50; post-CR 47.40 ± 10.18; pre-UC 40.80 ± 8.20; post-UC 37.40 ± 9.70; Significant difference within CR group (95% CI of change 6.05, 11.34) following intervention. There is significant difference between CR and UC groups (p < 0.01). MCS pre-CR 40.60 ± 9.70; post-CR 52.10 ± 13.40; pre-UC 44.50 ± 9.10; post-UC 46.40 ± 9.90. Significant difference within CR (95% CI of change 8.02, 14.98) groups and significant difference between groups (p < 0.01) following intervention. Depressive symptoms (HADS) (6 months) pre-CR 3.38 ± 3.50; post-CR 3.21 ± 0.21; pre-UC 2.81 ± 3.60; post-UC 2.14 ± 0.22; Significant difference within CR group (95% CI of change −2.04, −1.04) following intervention. There is significant difference between CR and UC groups (p = 0.03). Anxiety (HADS) (6 months) pre-CR 7.43 ± 3.22; post-CR 8.97 ± 1.92; pre-UC 7.07 ± 3.16; post-UC 7.61 ± 3.09; Significant difference within CR group (95% CI of change −1.34, −0.46) following intervention. There is significant difference between CR and UC groups (p < 0.01). Tobacco Use (%) (6 months) pre-CR 39 (68.40%); post-CR 32 (56.14%); pre-UC 45 (65.21%); post-UC 38 (55.07%); Significant difference within CR group (95% CI of change −18.5%, −5.9%) following intervention. No significant difference between groups. Medication adherence (proportion with 80% of days covered) (6 months) pre-CR 28 (49.12%); post-CR 41 (71.92%); pre-UC 35 (50.72%); post-UC 47 (68.11%); Significant difference within CR group (95% CI of change −37.3%, −14.1%) following intervention. No significant difference between groups.

ǂWorld Health Organization region classification for countries.

*outcome could not be pooled in meta-analysis because scores were computed differently.

∫Only post-intervention data are available.

¶ SD not reported in the study.

§ Data reported as median and IQ.

‡Medication adherence was derived by summing the individual items from 8-item questionnaire in Moriscky medication adherence scale-8 (values from 0 to 8) and then categorized into 2 groups: high adherence (score = 0) and low adherence (score ≥ 1).

ǁNote: sample size was somewhat inconsistent between publications.

6MWD, 6-Minute walk distance; AC, active comparison; ACS, Acute coronary syndrome; ANS, Autonomic nervous system; CABG, coronary artery bypass grafting; CAD, coronary artery disease; CADE-Q II, Coronary Artery Disease Education Questionnaire II; CADE-SV, Coronary Artery Disease Education Questionnaire- Short version; CR, cardiac rehabilitation; CR/SP needs total, Cardiac rehabilitation/secondary prevention needs total; CCR, comprehensive CR; CRNAT, cardiac rehabilitation needs assessment tool; CHD, Coronary heart disease; CVD, cardiovascular diseases; DASI, Duke Activity Status Index Score; DBP, Diastolic blood pressure; DLCO, Diffusion factor of the lung for carbon monoxide, DRT, Deep Relaxation Technique; e/a ratio, early to late diastolic trans-mitral flow velocity ratio; EF, Ejection fraction; FBS, fasting blood sugar; EQ-5D-5L, 5-level EuroQol-5D version; FEV1, Forced expiratory volume in 1st second; FFQ, 14-item Food Frequency Questionnaire for cardiovascular prevention; FITT, Frequency, intensity, time, type; FVC, Forced vital capacity; GAD-7, Generalized anxiety disorder assessment scale; HADs, Hospital Anxiety and Depression Scale; HbA1_C_, Hemoglobin A1_C_; HDL-C, High density lipoprotein cholesterol; HF, heart failure; HIIT, high intensity interval training; HR, Heart rate; Hs-CRP, High-sensitivity C-reactive protein; IMPACS, Impact of text Messages in a middle-income country to Promote secondary prevention after ACS;IPAQ-SF, International Physical Activity Questionnaire Short Form; ISWD, Incremental Shuttle Walk Distance; ITT, intention-to-treat; IQ, Interquartile range; KCCQ, Kansas City cardiomyopathy questionnaire; LDL-C, Low density lipoprotein cholesterol; LF/HF ratio, low and high frequency power component ratio for heart rate variability; MBP, Mean blood pressure; MCS, Mental component summary; MacNew QLMI, MacNew Quality of Life after Myocardial Infarction; METs, Metabolic equivalent of tasks; MI, Myocardial Infarction; MVV, Maximum voluntary ventilation; NA, Negative Affect of PANAS scale; NR, not reported; NSP, Nadi Shuddhi Pranayama; NT-proBNP, N-terminal fragment of Brain Natriuretic Peptide; Non-veg., non-vegetarian; NYHA, New York Heart association; PANAS, Positive And Negative Affect Scale; PA, Positive Affect of PANAS scale; PCI, Percutaneous coronary intervention; PCS, Physical component summary; PEFR, Peak expiratory flow rate; PHQ-9, Patient Health Questionnaire-9; PMR, Progressive muscular relaxation; PROBE, prospective, randomised, open, blinded end-point; pNN50, percentage of number of pairs of adjacent RR intervals differing by >50 ms; PSS, Perceived Stress Scale; PVO_2_-DASI, Peak oxygen consumption estimated from the Duke Activity Status Index questionnaire; PVO_2_-VSAQ, Peak oxygen consumption estimated from the Veteran's Specific Activity Questionnaire; PVCs, Premature ventricular contractions; QoL, Quality of Life; QRT, Quick Relaxation Technique; RMSSD, root mean square of successive RR interval differences; RR, respiratory rate; RRIV, R-R interval variation; SAHLPA-18, Short Assessment of Health Literacy for Portuguese Speaking Adults; SBP, Systolic blood pressure; SD, Standard deviation; SDNN, standard deviation of normal-to-normal RR intervals; SDSD, standard deviation of successive RR interval differences; SF-12v2, 12-Item short form QoL questionnaire version 2; SF-36, 36-Item short form QoL questionnaire; SMART-CR/SP, Smart phone-based CR and secondary prevention; STAI, Spielberger's State-Trait Anxiety Inventory; SVC, Slow vital capacity; UC, usual care; Veg., vegetarian; VLDL-C, Very Low density lipoprotein cholesterol; VO_2 Peak_, Maximum oxygen consumption; WBCs, White blood cell count; WHOQOL-Bref, World Health Organization Quality of Life- Bref; Y, yes; pg/ml, picogram per millilitre; bpm, beats per minute; kpm/min, kilopond meters per minute; L, Litre

A published protocol for an on-going trial was also identified.^30^ We were involved in another CR trial in Iran which is now in press.^31^ These should be included in a future update of this review.

### Trial Characteristics

Out of 135 LMICs, trials were performed in 8 (5.92%) countries, namely: India (*n* = 6 trials), Iran (n = 6), Brazil (*n* = 4), Egypt (n = 4), China (*n* = 2), Pakistan (n = 2), Nigeria (*n* = 1), and Bangladesh (n = 1; Table 1). Thus, there were trials undertaken in 5 out of the 6 WHO regions: Eastern Mediterranean (*n* = 12 trials), South-East Asia (*n* = 7), Americas (n = 4), Western Pacific (n = 2) and Africa (n = 1) (none in Europe).

As also shown in Table 1, included trials were undertaken from 2008. Three of the 26 trials were multi-centre.^32^, ^33^, ^34^ Follow-up duration ranged from 4 weeks to 5 years, with a median of 12 weeks.

Regarding trial design, no included trials were cluster randomised; all had parallel arms only (we did not extract the 12 month data from the Brazil trial as there was crossover, but the 6 month outcome data).^35^^,^^36^ Sixteen trials (61.53%) had UC controls, eight (30.76%) had an AC arm, and two (7.69%) had both (3-armed trials).^37^^,^^38^ AC involved for example exercise-only CR, or CR with education and/or psychological counselling, offered supervised or unsupervised.

### Characteristics of Participants

Trials included a recruited total of 6380 patients, with sample sizes ranging between 30 and 3959 patients (median = 72; Table 1). Six (23.1%) trials enrolled patients with HF, for a total of 306 (4.8%) patients. The average age of the participants in the trials included ranged between 48 and 63 years (median = 53.6). In total, 1069 (16.9%) trial participants were female, and three trials included no females.^26^, ^27^, ^28^^,^^39^^,^^40^ No trial reported on the ethnocultural background of participants.

#### Characteristics of CR programs

As shown in Table 2, six (23.0%) trials started with phase I CR. Four (15.3%) trials offered exercise only; the other components offered in the other 22 trials are shown in the Table. Three (11.5%) trials offered yoga. Four (15.3%) offered resistance exercise along with aerobic. Available aerobic exercise prescription details for each trial are shown in the Table.

**Table 2:** Characteristics of CR.

Study Author/Trialα, Year, Country	Session dose (frequency of human contacts [remote or face-to-face]/week x weeks); mins/session	Setting; Phases; Technology; Deliverers	Exercise Intervention (FITT)	Other components; theory
Abolahrari-Shirazi 2018,^38^ Iran	21 sessions (three times/week for 7 weeks [face-to-face & remote both]); 15–45 min/session	Hospital; II; Technology-No; physiotherapist, nurse	Participants performed ET and CT based on the group allocated with a total of 45 min at 40%–70% peak VO_2_ predicted with a supervised graded exercise test on a treadmill with the Bruce protocol; Resistance exercise also gradually increased in intensity from 40% one RM to 60% 1RM and included four exercise regimens: knee extension, knee flexion, elbow flexion, and shoulder abduction; The 45 min exercise sessions were composed of 5 min of warm-up, 20 min of aerobic exercises, 15 min of resistance training and 5 min for cool down; treadmill, cycle and arm ergometer, weight machine, dumbbells;	Other components: not specified; Theory: no.
Abdelhalem 2018,^43^ Egypt	24 sessions (two times/week for 12 weeks [face-to-face]); 45 min/session	Hospital; II; Technology-No; Deliverers- not specified	Supervised exercise consisted of 5 min of warm-up exercises followed by 30–35 min of continuous exercise [Alternating brief (2–5 min) higher intensity which aiming to reach 85–95% of participants' initial heart rate reserve and similar time of moderate-intensity workloads throughout an exercise session], and end by 5 min of cool down.; treadmill;	Other components of CR: Participants were provided education about heart disease and importance of risk factor modification in addition to advices regarding home-based activities. Theory: no.
Ajiboye 2015,^55^ Nigeria	36 sessions (three times/week for 12 weeks [face-to-face]); 60 min/session	Hospital; II; Technology-No; Researcher and a research assistant	Participants received an individualized exercise prescription based on their tolerance and was maintained at 60–70% of their peak heart rate; Each exercise session consisted of 10 min warm-up phase, 20-min aerobic phase, 20 min of strength/resistance training, and 10- min cool-down phase; bicycle ergometer, dumbbells, hand dynamometer, sand bags of known weight;	In the CR arm, patients were offered 3 education sessions, prior to starting the study, at the end of 6th week and at the end of 12th week. Sessions included general health talk on prevention of complications, lifestyle modification, and healthy living. Theory: no
Aslanabadi 2008,^56^ Iran	23 sessions (over 2 years [15 face-to-face & 8 remote sessions]); 60 min/session	Hospital and home; II; Technology-Yes (8 phone calls over two years); Program manager, exercise leader	Participants in intervention group were prescribed exercise for 60 min consisting of a warm-up followed by specifically designed heart targeting aerobic exercise. The exercise consultation was based on patients' risk factor profile; Resource requirement- not specified;	In intervention arm, The Participants received an individually based stage of the change-oriented lifestyle counselling. The counselling was established featuring five using areas of a routine cardiac rehabilitation program namely stress management techniques, weight reduction, reduction of alcohol consumption and dietary modification guide. Theory: no.
Babu 2011,^44^ India	12 sessions (average 4 sessions during phase I[face-to-face] and then once weekly for 8 weeks [Remote] in phase II); duration of each session not specified	In Hospital during phase I; Home program during phase II; Technology-Yes (8 phone calls); Physicians, physiotherapist	Supervised session frequency once daily with average 4 sessions and patients were directed to exercise in their home during phase II; Participants received an individualized exercise prescription using the modified Borg's RPE between 3 and 4/10 during in hospital phase and gradually increase intensity to 4–6/10 RPE with increasing the duration and frequency of exercise at home; walking, upper and lower limb exercises;	In the CR arm, not specified by other components though, but it was mentioned that patients and their relatives were taught to identify signs to stop performing the exercise, for example, heavy exertion (RPE >7) and chest pain (>5 on the visual analogue scale). Theory: no.
Chanrdrasekaran (Yoga-CaRe Trial) 2019,^33^^,^^47^^,^^57^ India	13 supervised sessions (spread over 12 weeks initially twice weekly then once weekly [face-to-face & remote both]; 75 min/session.	In Hospital during phase I; Home program during phase II; Technology-Yes (Telephone follow up for those missing in person session); multidisciplinary team including yoga specialist;	Participants in Intervention group were taught two basic components of yoga (breathing exercises, then meditation & relaxation practices) on 1st formal outpatient session individually 15 min each for 30 mins. Then for the rest of the formal sessions, in addition to above, participants were taught health rejuvenating exercises (9 mins), different yoga poses (standing, sitting and lying poses for about 25 mins).	In Intervention arm, participants attended exercise-cum-education sessions during formal outpatient classes to encourage maintenance of dietary and lifestyle changes and self-practice of Yoga at home. These included a combination of exercises related to general physical fitness, stress, and relaxation (e.g., meditation, breathing exercises) and also some exercises that were known to be cardioprotective in yogic texts through an instruction booklet and DVD in local language; Theory: no.
Chaves/Ghisi/Britto 2019,^35^, ^36^, ^37^^,^^58^ Brazil	36 sessions (in decreasing frequency from three times to once/week for 24 weeks [face-to-face]); 60–90 min/session	Hospital; II; Technology-No; Physicians, physiotherapist and dietitian	Supervised session frequency varied (see dose column) and patients were directed to exercise in their communities on the days they were not on site; Participants received an individualized exercise prescription based on a graded exercise stress test, and were instructed to exercise between 50% and 80% of heart rate reserve; The 1 h exercise sessions were composed of 10 min of warm-up, 30 min of aerobic exercises, 15 min of resistance training and 5 min for cool down; treadmill, bike and walking	In the comprehensive CR arm, patients were additionally offered 24 education sessions, supported by a workbook (https://www. healtheuniversity. ca/en/cardiaccollege). Sessions covered diet, exercise, mental health and risk factor management. These were delivered in a group setting, each for 30 min, just prior to or after an exercise session. Theory: yes- adult education principles and Health Action Process Approach.
Dehdari 2009,^59^ Iran	39 sessions (Twelve supervised PMR sessions; three education and three times weekly for 8 weeks CR exercise sessions [face-to-face and remote both]); 40 min/session	In Hospital; II; Technology-no; Physiotherapist	Participants performed PMR exercise three times a day on average along with CR exercise training three times per week. A relaxation audio CD was provided to guide on how to do PMR at home.	In both arms, participants received educational sessions three times in the total study period with focus on lifestyle modification. Theory: no.
Dorje 2019,^29^^,^^60^ China	34 sessions (thirty-two online health education modules through WeChat [remote] and two baseline assessment sessions [face-to-face]); session duration-not specified	In patients' home; II; Technology-Yes (software used through Smart phone [4 education articles/week through WeChat software in cell phones for 8 weeks then, 2 articles/week for 16 weeks]); Cardiac Rehabilitation/secondary prevention coach	Participants received an individualized walking prescription based on their baseline 6MWT, with both the time and intensity of walking increased gradually over the first eight weeks. Participants were encouraged to perform other forms of physical activity, such as swimming, Tai Chi, group dancing and table tennis.	In intervention arm, participants received 32 educational modules with each consisted of 25–30 slides of cartoon images and introduced a key knowledge theme through dialogue between patient and provider. Theory: no.
Eraballi 2018(x2) Raghuram 2014,^26^, ^27^, ^28^ India	Average 58–62 sessions (among them ten face-to-face and forty-eight remote sessions over phone for 12 months); 45 min/session	in hospital and patient's home; Both I and II; Technology-Yes (weekly phone calls for 12 months); Pharmacist, nutritionist, physiotherapist and Yoga therapist	Participants were trained to practice Yoga (DRT, QRT and NSP for 20 min) four times/day during pre and post-operative period in hospital. They also practice yoga using a pre-recorded audio tape.	Participants were taught Yoga modules through pre-recorded DVD with Yoga practices with instructions along with diet and counselling on lifestyle modification. They were provided an e-book ‘yoga for hypertension and heart diseases’; Theory: no
Farheen/Khalid 2019,^61^^,^^62^ Pakistan	18 sessions (three times/week for 6 weeks [face-to-face]); 35–40 min/session	in hospital; II; Technology-No; A multidisciplinary team including cardiologist, cardiopulmonary rehabilitation specialist and PhD physiotherapist	All patients performed three sets of aerobic exercises (two sets of 6 min cycling and 1 set of treadmill walking for 6 min) at intensity of at 65–85% of THR; Participants in CR group also performed resistance training at an intensity of 30–50% of 1 repetition maximum (1 set of 10–12 reps). Intensity was gradually increased weekly along with weights and frequencies based on patient's THR. Cycling, weight-lifting, walking;	Other CR components- not specified; Theory: no
Hasanpour 2020,^42^ Iran	72 sessions (three times/week for 24 weeks [face-to-face]); 40 min/session	Hospital; II; Technology-No; Nursing and medical teams, cardiologist	Supervised exercise session consisted of 5–10 min of warm up, 25–30 min of walking and 5 min of cool down; gradually increasing the intensity and duration of walking keeping 70% of HR reserve; exercise was stopped if signs of discomfort or endanger to health based on Rhoten fatigue scale.	Other CR components- not specified; Theory-no
Hassan 2016,^63^ Egypt	72 sessions (three times/week for 24 weeks [face-to-face]); 40–50 min/session	Hospital; II; Technology-No; physiotherapist;	Participants in CR group performed aerobic exercises on bicycle ergometer for 50 mins. Individually prescribed exercise based on Borg's RPE in which patients were encouraged to maintain a rating between 11(fairly light) and 14 (hard); The session comprised of 5–10 min of warm-up, 30 min of aerobic exercises, and 5–10 min for cool down; bicycle ergometer;	In intervention arm, patients were provided educational program on secondary prevention and risk factor control according to AHA guidelines 2011. Theory: no.
Haq 2019,^64^ Pakistan	12 sessions (6 during Phase I and 6 during Phase II over 8 weeks [face-to-face only]); 30 min/session	Hospital; I & II; Technology-No; multidisciplinary team comprising one cardiac consultant, two trained nurses, 1 physiotherapist, and 1 dietitian	Initial in hospital sessions were focused on early mobilization starting from 1 to 3 min' walk with gradual increasing in time and intensity. Structured exercise programs were offered soon as the patients were in phase II. Exercise program consisted of a supervised 30 min aerobic and strength exercise training sessions. No other details were provided as per FITT.	In intervention arm, patients were offered two dietary counselling session of 15–30 min per week for two weeks and two cardiologist consultation sessions on psychosocial and risk factor management during phase I; education sessions of 15–30 min on the same day with exercise session during phase II. Theory: no.
Jena 2020,^32^ India	30 sessions (once/day for 30 days [face-to-face & remote both]); 30 min/session	Hospital; I; Technology-Yes (phone calls- those who discharged before 30 days of training were followed up through phone or home visit); Deliverers- not specified;	Participants in CR group performed aerobic exercise such as stretching, walking and bicycling from low to high intensity in hospital; No other details of exercise sessions provided;	Other CR components- not specified; Theory: no.
Lima 2020,^46^^,^^65^ Brazil	60 sessions (Five times/week for 12 weeks [Two face-to-face(during 1st and 2nd week of intervention) & rest at-home]); 60 min/session	Both Hospital and at-home; II; Technology-yes (weekly phone calls); physiotherapist;	Supervised exercise session was performed using treadmill, bicycle or walk. Each session consisted of five to ten minutes of warm-up, 40 min of aerobic activity and five to ten minutes of cooling. The exercise prescription was individual and based on the exercise test. Participants were instructed to perform aerobic exercise at 60%, 70% and 80% of the reserve HR in the first, second and third months, respectively. Patients were provided a HR monitor and a Pedometer to monitor number of prescribed exercise and compile in training logbook.	Other CR components- both arms received six 40 min education session about following topics: diet, exercise, mental health and control of risk factors. Theory: no.
Mehani 2018,^40^ Egypt	36 sessions (three times/week for 12 weeks [face-to-face]); 60–70 min/session for resistance and 45 min/session for aerobic exercise	Hospital; II; Technology-No; physiotherapist	Participants in intervention group performed both aerobic (about 9 bouts) and resistance (8 bouts) training with active rest for 45 s in between. The exercise training followed a sequence like: aerobic exercise training on treadmill –active rest – resisted exercise for one muscle group with known weight – active rest – aerobic exercise on bicycle ergometry – active rest –and then resisted exercise for another muscle group with known weight – active rest and so on till completing the eight muscle groups according to the one repetition maximum for each for resistance exercise. Exercise was prescribed individually according to the THR for each patient and gradually increased in intensity as follows: first and second week: 65% of THR, third and fourth week: 70% of THR, fifth and sixth week: 70% of THR, seventh and eighth week: 75% of THR, ninth and tenth week: 75% of THR and eleventh and twelfth week: 80% of THR. Resistance exercise is graduated every three weeks as follows: one set with 10 repetitions for each muscle group, then one set with 15 repetitions for each muscle group, followed by two sets with 10 repetitions for the same muscle groups and finally two sets with 15 repetitions in the last three weeks of the study. Each day the training session started with 5 min warming up with treadmill walking and ended by 5 min cooling down in the same manner. The intensity of the resistance exercise training was graded from 50% of the one repetition maximum test at the beginning of the program to about 70% of the one repetition maximum test at the end of the training program. Treadmill, cycle ergometer, weight bags;	Other CR components- not specified; Theory-no.
Mehani 2013,^39^ Egypt	84 sessions (three times/week for 28 weeks [face-to-face]); 45–60 min/session	Hospital; II; Technology-No; Multidisciplinary team- physician, physiotherapist, dietitian	Participants in intervention group performed supervised exercise including 5–10 min warm-up phase by pedaling on bicycle ergometer with 60 rpm, slow walking on treadmill with 1.2 m/h or stretching exercises with breathing. They also performed circuit aerobic interval training which were made progressively more difficult by performing the exercise in more challenging ways. The treadmill speed, inclination or bicycle resistance was set at the highest comfortable setting that was safe for the patient according to his target or training heart rate, This phase also started in short bouts about 8 min for 24 min, gradually prolonged up till continuous 45 min at the end of the 7th months. Finally, cool down phase for 10 min; For treadmill training, the speed was increased till reaching 4–5 m/h at the end of the 7th month. For cycle ergometer training, the repetitions/min was increased till reaching 80 repetitions per minute (rpm) at the end of the 7th month. The training heart rate increased gradually according to each patient's response during exercise training session, starting with 55% of heart rate reserve, till reaching 80% at the end of 7th month. Treadmill, cycle ergometer, Transthoracic Doppler Echocardiography (Hewlett–Packard Sonos, USA), Cardiopulmonary exercise testing (CPET) by Oxycon pro (Jaeger – Germany);	Other CR components- education sessions on disease information aimed to reinforce Patients' knowledge about chronic heart failure signs and symptoms, ensure compliance with medications, identify recurrent symptoms amenable to treatment, and advice on how to live with heart failure. Dietary counselling also provided to recognize and self management of fluid overload. Theory: no.
Moeini 2015,^66^ Iran	16 sessions (two times/week for 8 weeks [face-to-face]); 45–60 min/session	Hospital; II; Technology-No; providers- supervised by cardiologist	Participants in intervention group performed resistance exercise for 20–25 min in addition to aerobic exercise. Each session started with warm up and ended with cool down through stretching for 10–15 min. For resistance exercise, the target weight was determined based on the heaviest weight each subject could lift for 12–15 times in the expected range of motion for the elbow, shoulder, and knee joints. The number of repetitions in the range of motion was initially 10, and then gradually increased to 15, then after reaching 15 repetitions, the weight was increased by 3–5% and number of sets also increased accordingly. For example, in sessions 3–7, participants performed one set of side lateral, front, and overhead raise, overhead triceps extensions, alternating biceps curls and shoulder press, and weight squad with 11 repetitions, then gradually for 15 repetitions. In the 8th session, both the number of the sets and the weight were increased (by 3–5% of the previous weight) in such a way that two sets of the above-mentioned exercises were performed with 10 repetitions, with a rest period of 1 min between the sets. In sessions 9–13, two sets of the above resistance exercises were performed with 11 and 15 repetitions in each session, respectively, and a 1 min rest period between the sets. In session 14, the number of the sets and the weight were increased again. In sessions 15–16, three sets with repetitions of 11–12 times were performed. The correct way of exercise and prevention of Valsalva manoeuvre were explained to the subjects; treadmill, cycle ergometer, weight machine, dumbbells;	Other CR components- proper nutrition education and psychiatry counselling sessions. Theory: no.
Passaglia 2020,^41^^,^^67^ Brazil	4 sessions over 6 months (we consider sessions to be in-person contacts or phone calls. 4 modules, 96 text messages [face-to-face & remote both]); session duration-not specified	At patient's home; II; Technology-Yes (SMS text 4 times per week for 6 months); Two medical students, 1 nutritionist and an endocrinologist;	In addition to standard usual care, the intervention group received SMS texts 4 times/week for 180 days at pre-established time which include a variety of topics, such as standard follow up care reminders and general self-management and healthy habits texts to inform and engage patients in care. All the participants had the opportunity to participate in cardiovascular rehabilitation program which include supervised physical exercise for three consecutive months. The exercise was not specified as per FITT.	In intervention arm, a total of 185 messages delivered on advice, motivation and information about medication adherence, increase of regular physical activity, adoption of healthy dietary habits and smoking cessation (if appropriate). Theory: no.
Salvetti 2008,^45^ Brazil	44 sessions (2 face to face then 6 phone calls each/2weekly + 36 unsupervised exercise 3 times/week for 12 weeks [face-to-face & remote both]); 50–60 min/session	Delivery in medical setting; II; Technology-yes (Phone calls biweekly for 12 weeks); physiotherapist, physician	Two supervised exercise sessions included a 10-min warm-up consisting of walking and stretching exercises, 40 min of aerobic exercise training consisting of walking and a 10-min cool-down period. Participants were instructed out-of-class training which include standard stretching exercises, walking three times per week for 30 min on non-consecutive days for three months, at the assessed target heart rate (60–80% based on cardiopulmonary exercise test and monitored by personal heart monitor); Treadmill;	In intervention arm, participants were provided education regarding exercise and coronary risk factors at the final 15 min of two supervised sessions. Theory: no.
Suleimani 2018,^68^ Iran	20 sessions (with varied frequency- 4 face to face and 16 phone calls for 8 weeks [face-to-face & remote both]); session duration- NR	Hospital; II; Technology-Yes (16 phone calls); Researcher, registered nurse	Participants in intervention group provided person centered nursing with advice on physical activity which is not specified as per FITT.	In intervention arm, participants received education session about knowledge on heart attacks, cardiovascular risk factors, nutrition, and dietary regimens, familiarity with cardiac medications and their method of usage, exercise, and physical activity. Theory: no.
Uddin 2019,^69^ Bangladesh	13 sessions (first session face to face then monthly follow up by phone calls for 12 months [face-to-face & remote both]); 45 min long for the 1st session	Hospital and at home; II; Technology-Yes (12 phone calls each per month); physician, physiotherapist;	Participants in intervention group were advised to exercise at least 30 min/day, including a 5-min warm-up and a 5-min cool-down, and need to be completed 4 days/week. Exercise was prescribed at an intensity of 11 to 13 on the Borg scale of rating of perceived exertion. Patients could choose activities involving use of large-muscle groups;	On the first in class session, participants in intervention arm were educated about psychosocial and self-care techniques by physiotherapist. Theory: no.
Venkatesh 2019,^70^ India	36 supervised sessions (three times/week for 12 weeks [face-to-face & remote both]); 40–50 min/session	Hospital and at home; II; Technology-Yes (phone calls to clarify any doubt of exercise prescription in CR group and weekly phone calls for 12 weeks for unsupervised AC group); Research team	Participants in study group performed supervised exercise consisted of 10 min warm up by breathing exercises, active movements of limbs and large muscle group, then exercise session of about 20 min and cool down for 10 mins; Patients started the exercise at low intensity initially and then gradually increased in intensity and duration; they were monitored before and after exercise for their heart rate and saturation using pulse oximetry and pulse rate and respiratory rate. If the exercise response is satisfactory, they were instructed to continue the same exercise in the home for 1 week. They were advised to continue walking sessions twice a day at home lasted for 20–30 min at an intensity tolerated by the patient without any symptoms. The training session included the strength and endurance training in alternative days, and walking session. The strength training was given based on one repetition maximum and the progression was done based on patients' exercise tolerance.	In both arms, participants received education session about secondary prevention measures based on their cardiac health. Theory: no.
Yadav 2015,^71^ India	60 sessions (yoga poses daily, 6 times/week for 10 weeks [1st session face-to-face & rest are remote]); 60 min/session	Hospital and at home; II; Technology-Yes (twice weekly phone calls); Yoga instructor, Dietitian	Participants in intervention arm were taught a standard yoga regimen on the 1st session with different yogasanas and pranayamas and then instructed to practice daily for 60 mins. The yoga regimen included deep breathing techniques for 35 mins, quick relaxation technique for 5 mins, deep relaxation technique for 5 min then pranayamas for 25 mins.	In intervention arm, the 1st session was ended by holistic teaching for 10 min and then dietary counselling by dietitian about healthy choice of food (i,e. diet rich in protein like pulses, green vegetables, juicy fruits, and very less fat. Theory: no.
Zhang 2017,^34^ China	12 sessions (varied frequency over 3 months [4 face-to-face & 8 remote sessions]); 60 min/session	In patient's home; II; Technology-Yes (regular telephone follow-up); Multidisciplinary team – physician, nurse, PT, psychiatrist, dietitians	Participants in study group performed exercise training including 10–20 min warm-up, 20–40 min aerobic exercise according to their preferred training modality in their home environment, then 10 min cool down and 20 min relaxation; The exercise prescribed at an intensity of 11–13 (fairly light to somewhat hard on the Borg scale). Participants were advised to walking at home or outside of the local surroundings, and they were able to choose other modes (e.g. using facilities in community leisure centers).	In intervention arm, participants were provided psychosocial and self-care techniques by physiotherapist. Theory: no.

FITT: frequency, intensity, time, type.

α, Chronological order; 6MWD, 6-Minute walk distance; AC, active comparison; AHA, American Heart Association; CD, Compact disc; CR, Cardiac rehabilitation; CT, combined endurance and resistance training; DRT, Deep Relaxation Technique; DVD, Digital versatile disc; ET, endurance training; HR, Heart rate; NR, Not reported; NSP, Nadi Shuddhi Pranayama; PMR, Progressive muscle relaxation; PT, Physical Therapy; QRT, Quick Relaxation Technique; RM, Repetition maximum; RPM, Repetitions per minute; RPE, Rating of perceived exertion; SMS, Short message service; THR, Target Heart rate;

Abbrev: min = minutes.

With regard to the CR program setting (Table 2), 14 (53.8%) trials were hospital-based, three (11.5%) were home-based, eight (30.7%) were hybrid (both hospital and home-based), and one (3.8%) was in a medical setting outside a hospital. In 14 (53.8%) trials, patients were contacted in CR using phone, of which two (7.6%) used smartphone-based software (i.e., WeChat) or text messages to provide CR.^29^^,^^41^

As also shown in the table, the average number of CR sessions offered ranged between four to 84 sessions (median = 32 sessions), at a frequency between two and six times/week, for six weeks to 24 months (median = 12 weeks); each session lasted between 30 and 120 min (median = 50 min). Types of healthcare providers delivering CR are also shown in Table 2.

### Risk of Bias and Certainty of Evidence

**Fig. 2:**
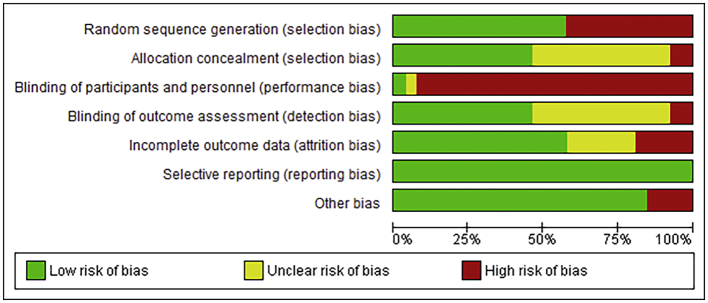
Risk of bias across all included trials.

Risk of bias for each included trial is shown in online Supplemental Fig. 1, and overall in Fig. 2. In 15 (57.6%) trials, analyses were performed on the basis of intention-to-treat. In no trials were the participants or providers blinded to allocation, as this would not be methodologically possible given the nature of CR; 12 trials reported using blinded outcome assessors. Overall, there was judged to be moderate risk of bias across trials. Certainty of evidence for each outcome is shown in Table 3a, Table 3b (by comparison).

**Table 3a:**
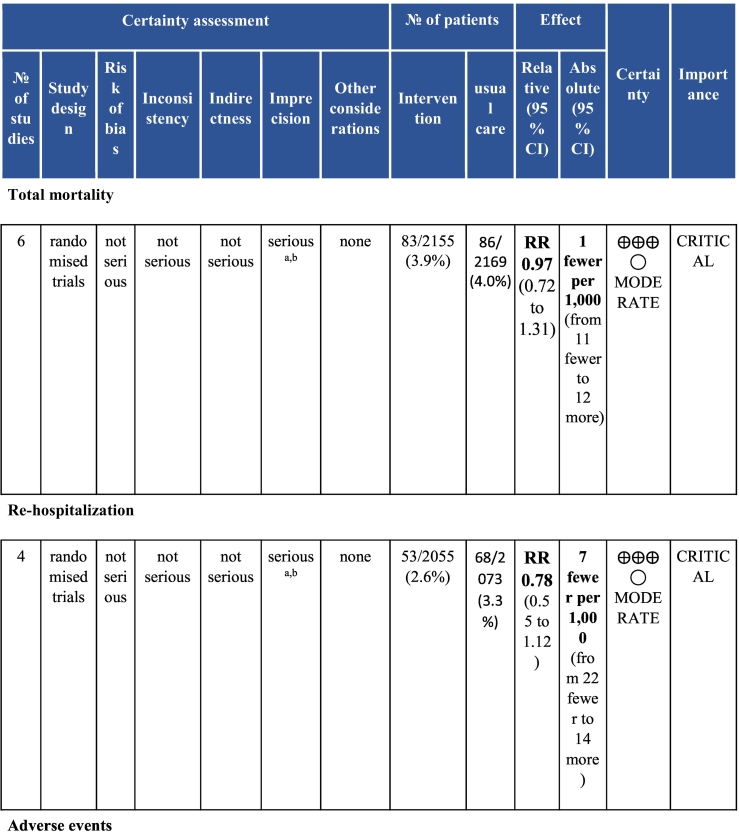

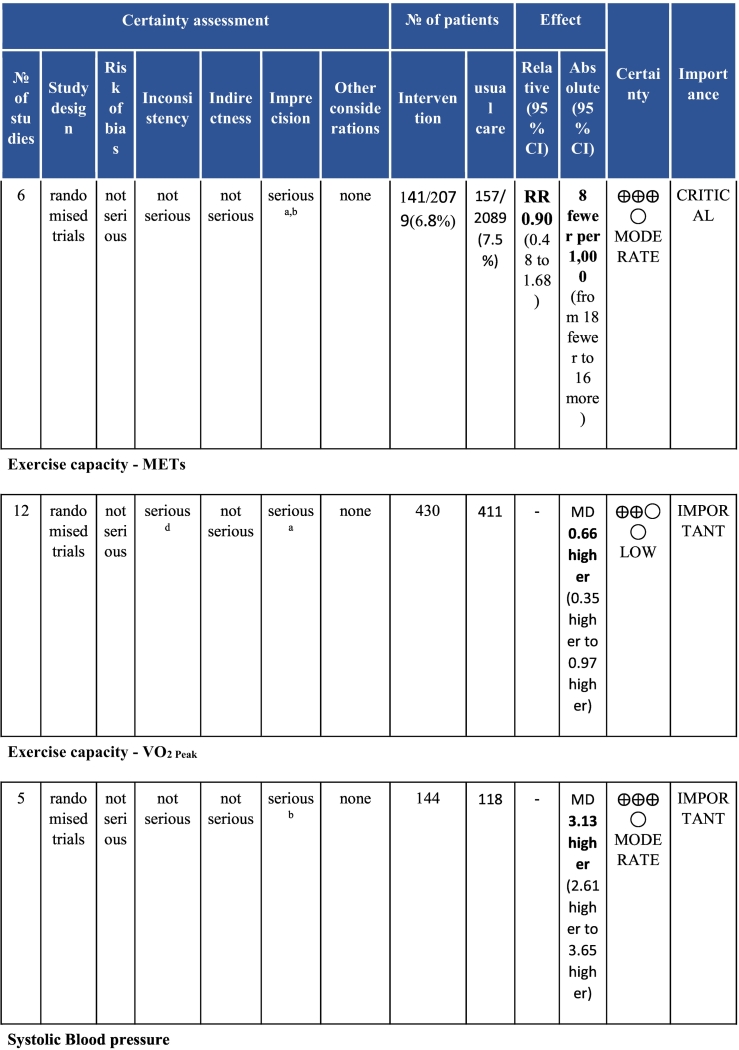

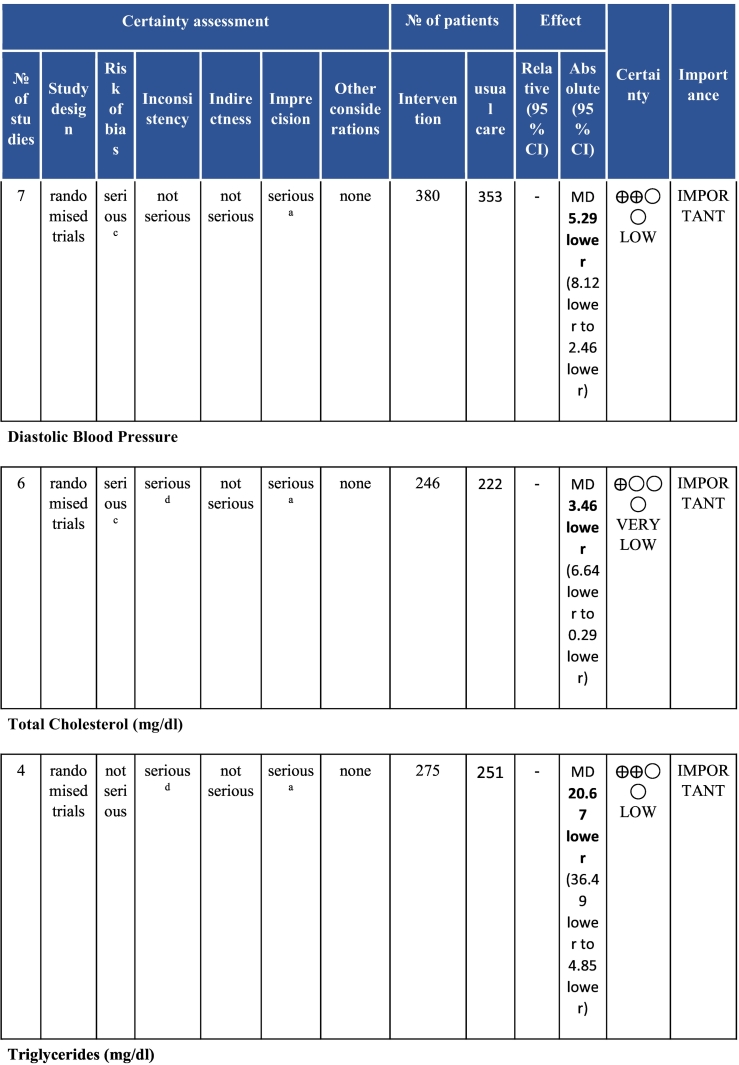

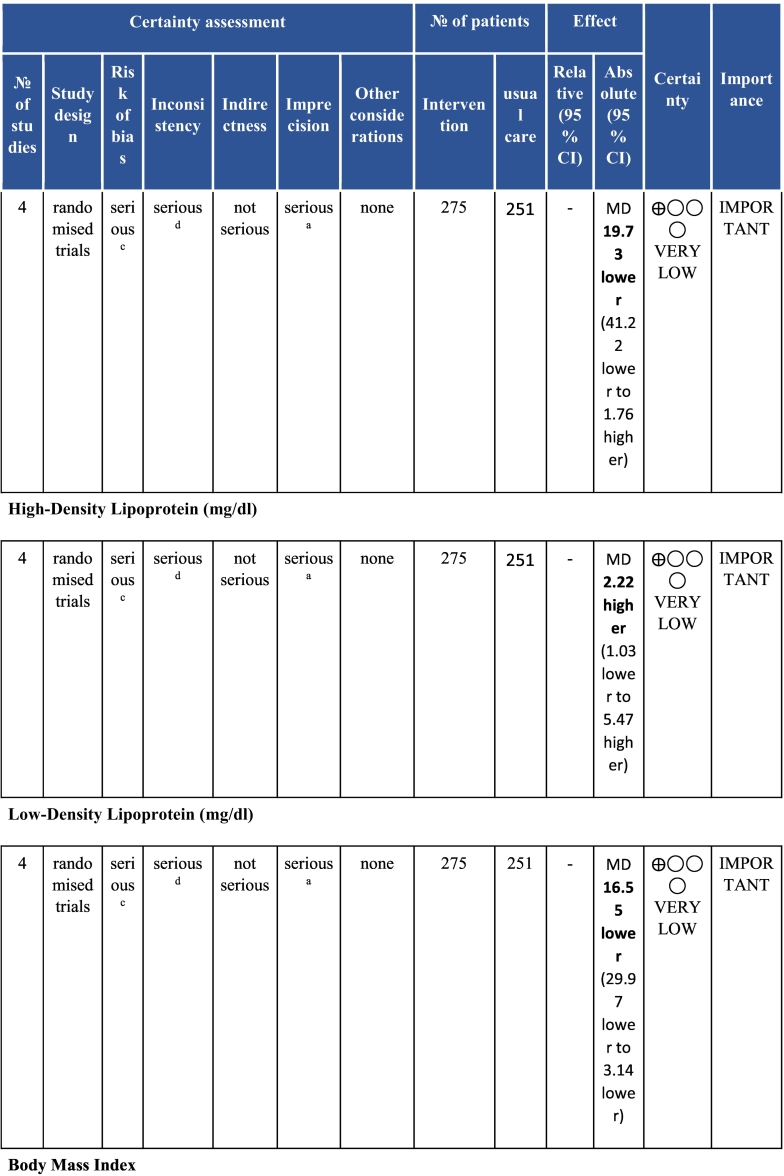

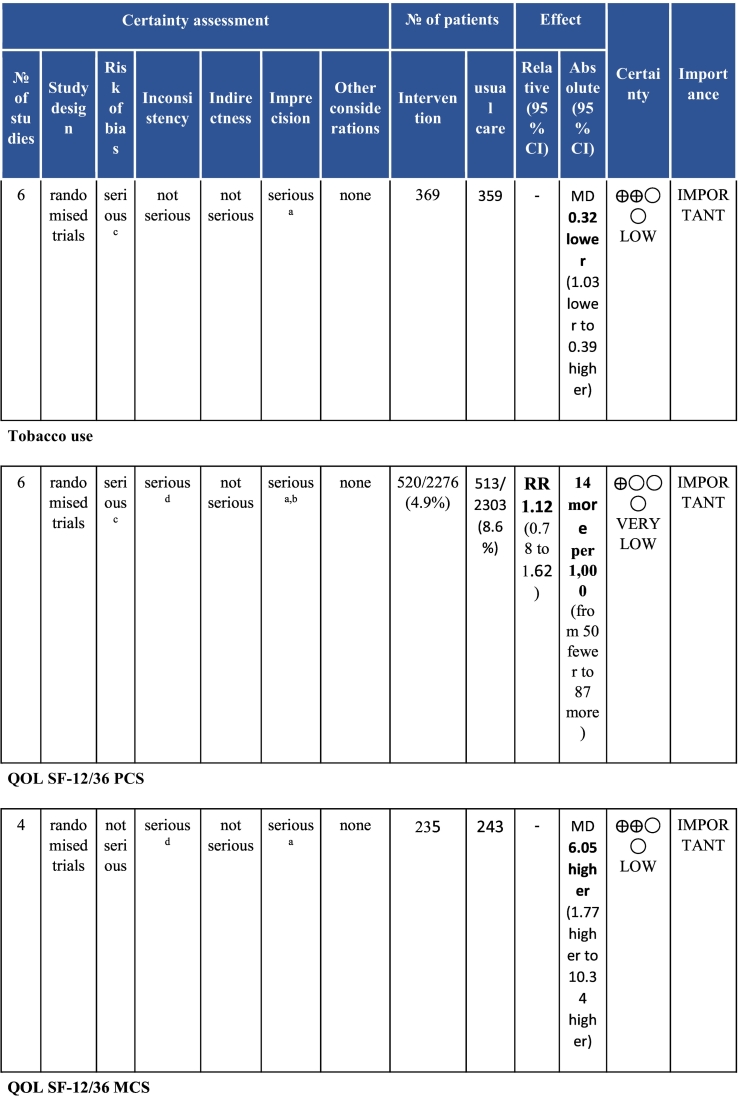

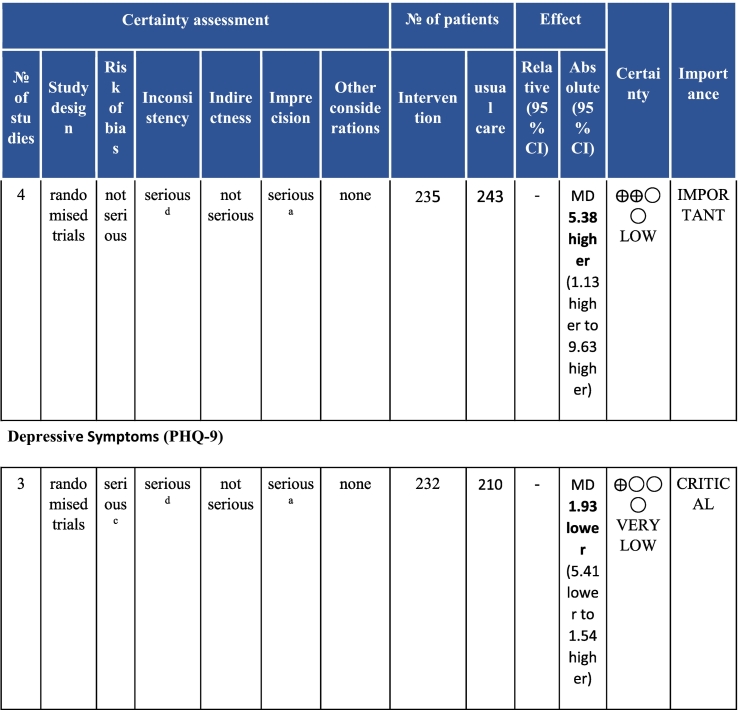
Summary of findings and certatpinty assessment: Intervention compared to Usual care.

CI: Confidence interval; RR: Risk ratio; MD: Mean difference; QOL SF-12/36 PCS, MCS: short-form quality of life survey from Rand Corporation, physical and mental component summary scores; METs: metabolic equivalent of tasks; PHQ: patient health questionnaire (https://www.phqscreeners.com/). Explanations: a. CI overlaps no effect and the upper and/or lower confidence limit crosses the minimal important difference (an effect size of 0.5 in either direction is used instead of calculating the effect size for each outcome measure). b. Total population size or number of events is less than 400. c. Inadequate allocation concealment in trials with >20% weight. d. P value for heterogeneity (chi square) is <0.05, I square is substantial >50%. High certainty means we are confident that the true effect lies close to that of the estimate of the effect. Moderate certainty means we are moderately confident in the effect estimate; the true effect is likely to be close to the estimate of the effect, but there is a possibility that it is substantially different. Low certainty means our confidence in the effect estimate is limited; the true effect might be substantially different from the estimate of the effect. Very low certainty means we have very little confidence in the effect estimate; the true effect is likely to be substantially different from the estimate of effect.^23^

**Table 3b:**
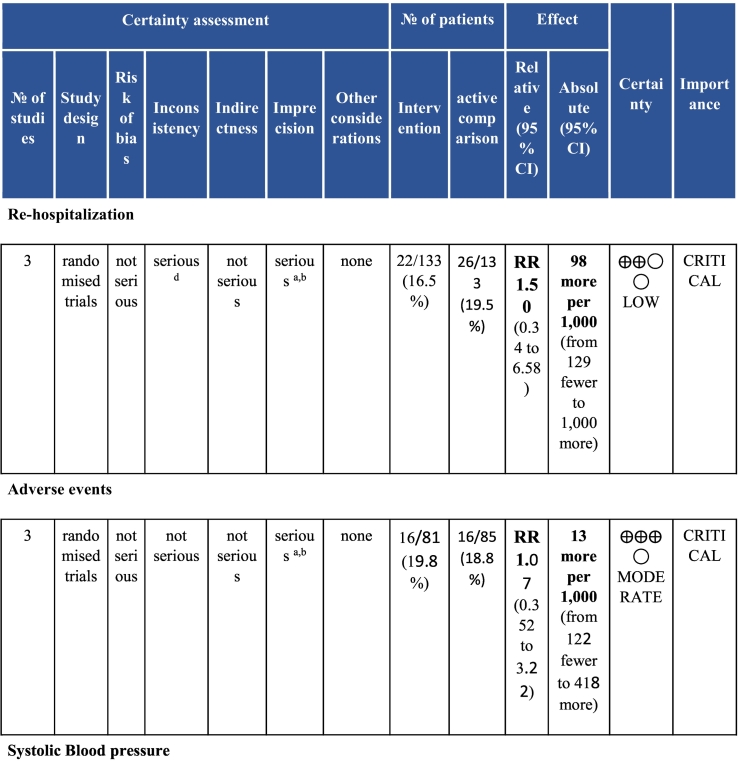

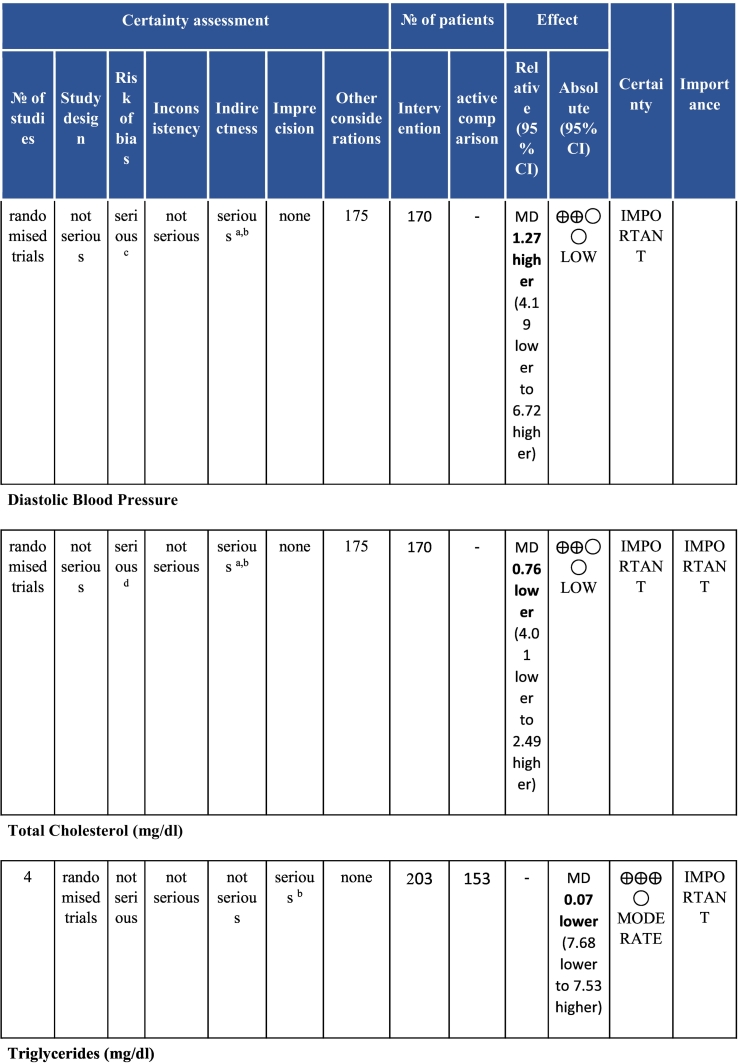

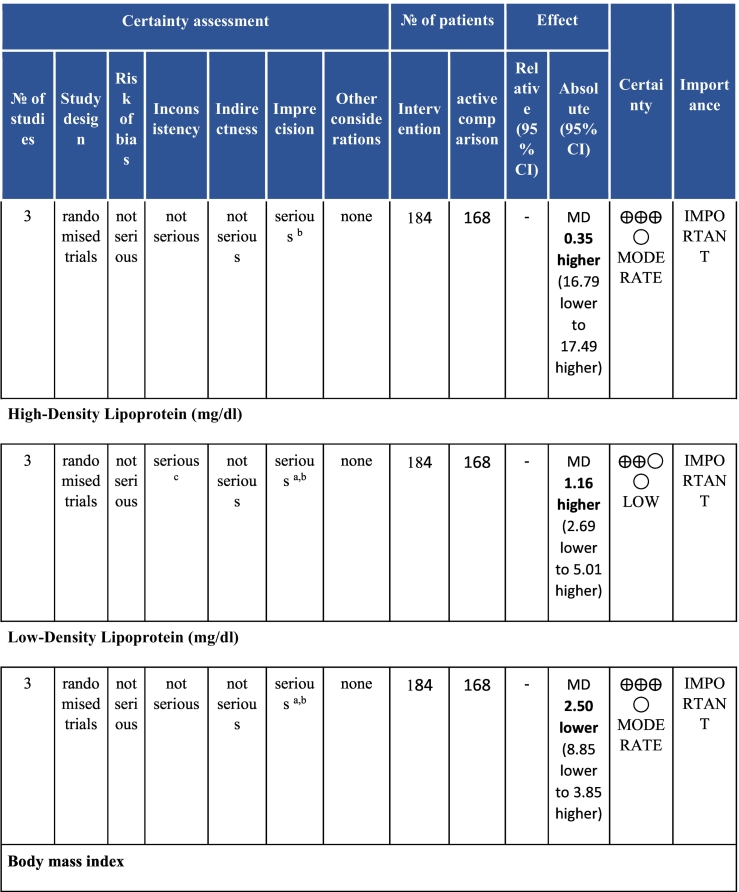

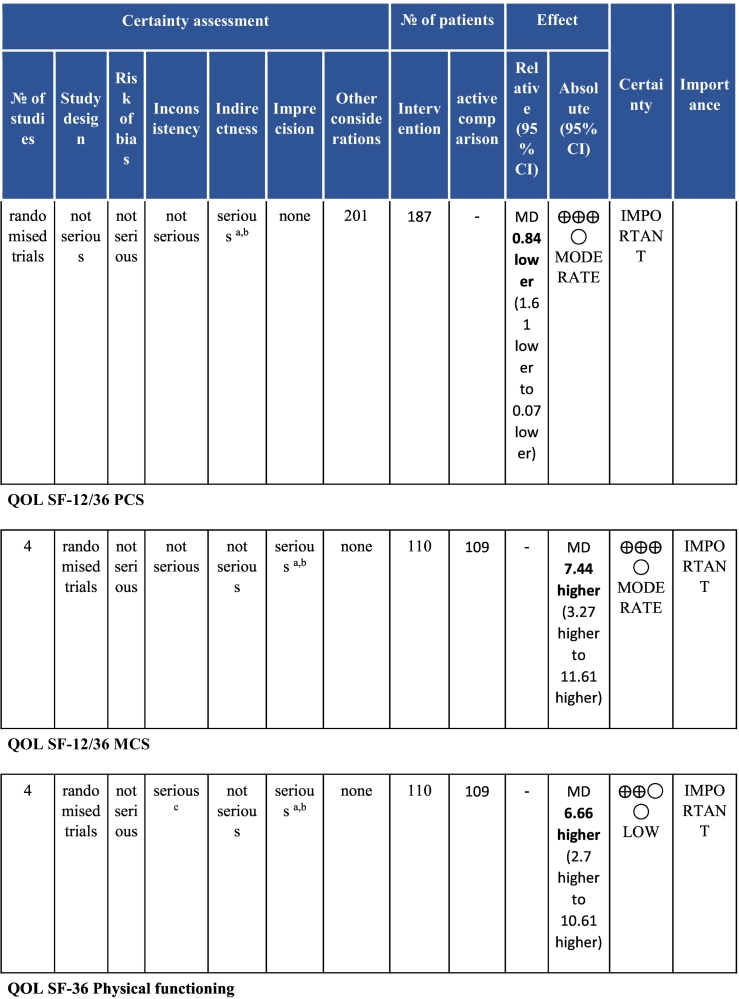

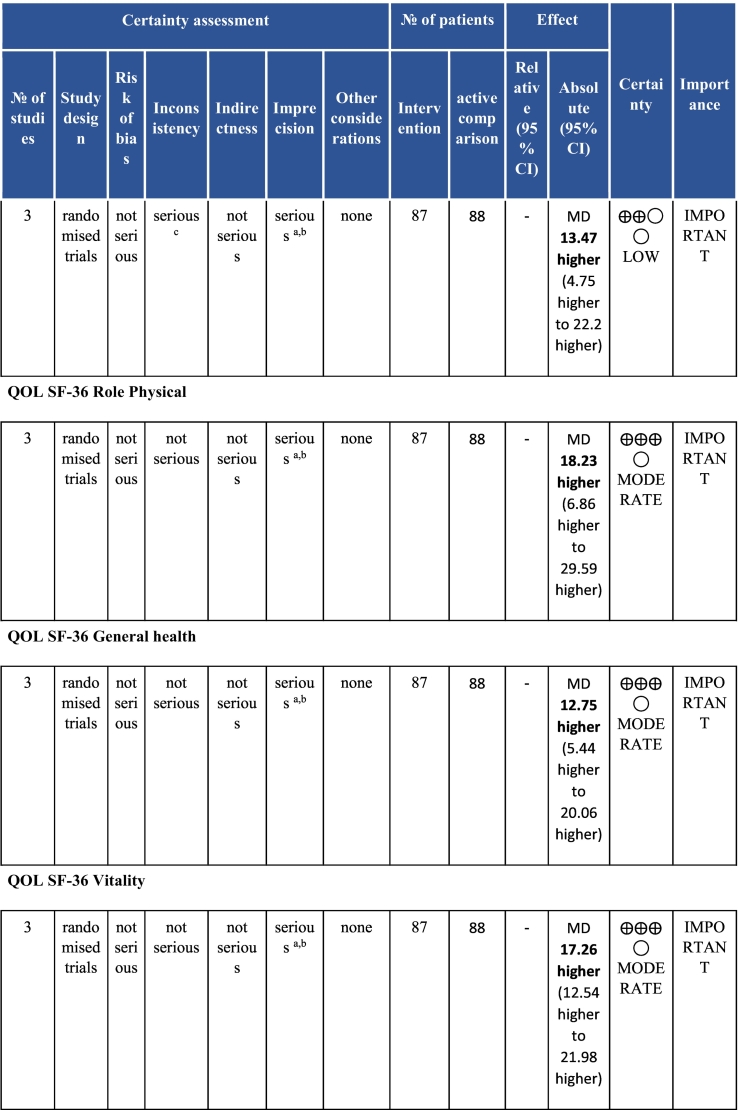

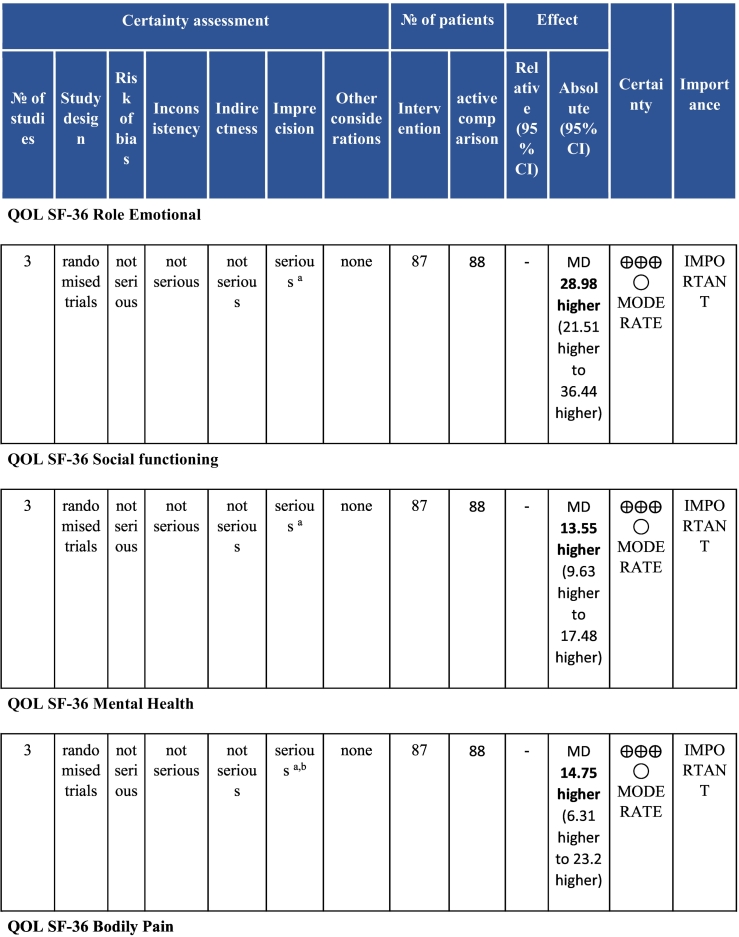

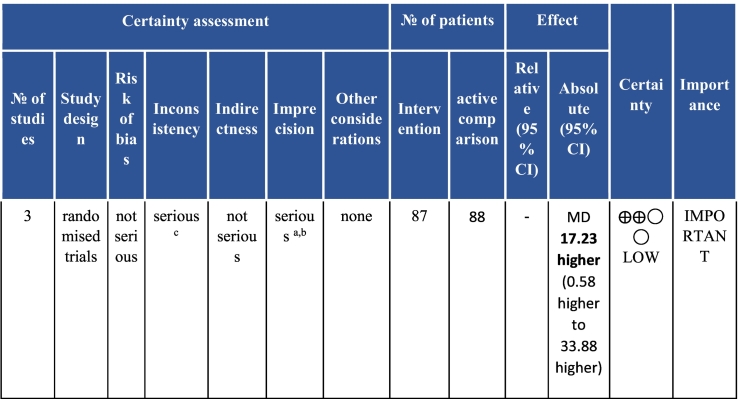
Summary of findings and certainty assessment: Intervention compared to active comparison.

CI: Confidence interval; MD: Mean difference; RR: Risk ratio; QoL SF-12/36 PCS, MCS: short-form quality of life survey from Rand Corporation, physical and mental component summary scores. Explanations: a. Total population size or number of events is less than 400. b. CI overlaps no effect and the upper and/or lower confidence limit crosses the minimal important difference (an effect size of 0.5 in either direction is used instead of calculating the effect size for each outcome measure). c. P value for heterogeneity (chi square) is <0.05, I square is substantial >50%. d. I square is substantial >50%.

### Meta-analysis Results

Twenty-five (96.1%) trials were included in the meta-analyses. Outcomes were reported by subgroup (NYHA [New York Heart Association] class) in one trial^42^; we could not secure the overall data from the authors and therefore did not include that trial. Due to unusual scores of all QoL domains of the SF-36 reported in Abdel-Halim et al.^43^ and that we failed to hear from the corresponding author, that outcome was not included for that trial in the meta-analysis. Table 1 qualitatively summarizes the findings of these trials for all outcomes. A summary of findings is shown in Table 3a, Table 3b (by comparison), and forest plots are shown in Fig. 3, Fig. 4, Fig. 5, Fig. 6, and online Supplemental Figs. 2–25.

**Fig. 3:**
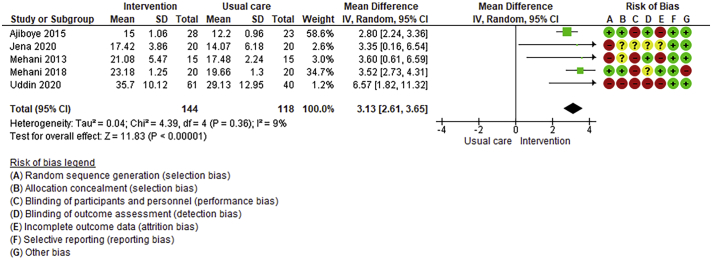
Forest plot summarizing effect of CR versus UC on Functional capacity- VO_2 Peak_. Legend: CR, Cardiac rehabilitation; UC, Usual care.

**Fig. 4:**
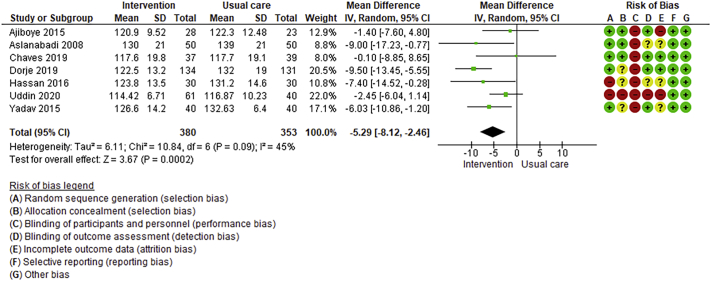
Forest plot summarizing effect of CR versus UC on systolic blood pressure. Legend: CR, Cardiac rehabilitation; UC, Usual care.

**Fig. 5:**
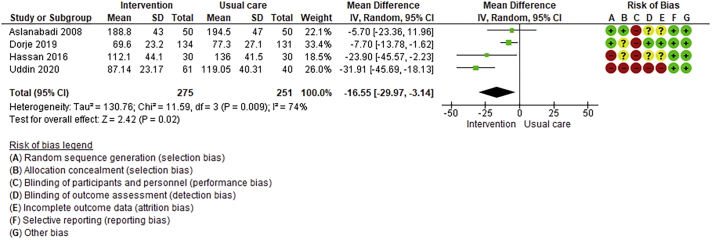
Forest plot summarizing effect of CR versus UC on LDL-cholesterol. Legend: CR, Cardiac rehabilitation; UC, Usual care; LDL, low-density lipoprotein.

**Fig. 6:**
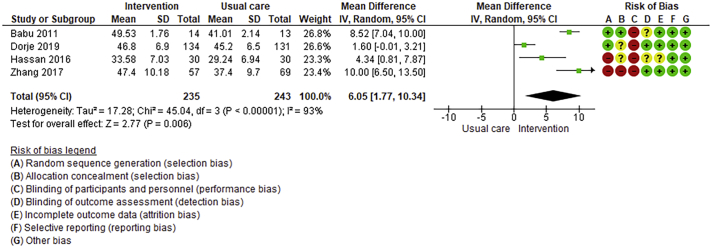
Forest plot summarizing effect of CR versus UC on QoL Physical Component Summary Scores (SF-12/36). Legend: CR, Cardiac rehabilitation; UC, Usual care; QoL, Quality of life.

### CR vs UC

As shown in the forest plots and Table 3a, for comparison to UC, meta-analyses were performed for the following outcomes: mortality, re-hospitalizations, adverse events, functional capacity (i.e., METs, VO_2_ peak), CVD risk factors (e.g., body mass index/BMI, blood pressure/BP, lipids, tobacco use), QoL (PCS and MCS scores), and depressive symptoms. Six trials reported a total of 169 all-cause deaths, with no evidence of a difference between CR and UC (Supplementary Fig. 6). With regard to morbidity, there were no significant effects of CR on re-hospitalizations or adverse events (Supplementary Figs. 7,8).

With regard to functional capacity specifically, compared with UC, the effects of CR in increasing VO_2_peak were meaningful (5 trials; participants = 262; MD = 3.13 ml/kg/min, 95% CI = 2.61 to 3.65; I^2^ = 9%; moderate-quality evidence; Fig. 3). Similarly, the effects of CR in increasing METs were meaningful (12 trials; participants = 841; MD = 0.66, 95% CI = 0.35 to 0.97; I^2^ = 68%; low-quality evidence; Supplemental Fig. 2).

There were no significant effects of CR on the risk factors of BMI, triglycerides or high-density lipoprotein, tobacco use, or depressive symptoms when compared to UC (Supplementary Figs. 9–13). CR did meaningfully improve the following risk factors: systolic BP (SBP; Fig. 4), diastolic BP (DBP; Supplemental Fig. 3), total cholesterol (Supplemental Fig. 4), low-density lipoprotein (LDL; Fig. 5, functional capacity (METs in Supplemental Fig. 2, and VO_2peak_ in Fig. 3). Specifically, with regard to risk factor control, compared with UC, the effects of CR were meaningful in reducing SBP (7 trials; participants = 733; mean difference [MD] = −5.29 mmHg, 95% confidence interval [CI] = 8.12 to -2.46; I^2^ = 45%; low-quality evidence; Fig. 4), DBP (6 trials; participants = 468; MD = -3.46 mmHg, 95% CI = -6.64 to -0.29; I^2^ = 75%; very low-quality evidence; Supplemental Fig. 3), as well as total cholesterol (4 trials; participants = 526; MD = -20.67 mg/dl, 95% CI = -36.49 to -4.85; I^2^ = 79%; low-quality evidence; Supplemental Fig. 4) and LDL cholesterol (4 trials; participants = 526; MD = -16.55 mg/dl, 95% CI = -29.97 to -3.14; I^2^ = 74%; very low-quality evidence; Fig. 5).

Finally, five trials reported QoL using a range of outcomes that included PCS, MCS of SF-12 and 8 domains of SF-36. For the SF-12/36 data which could be pooled, compared with UC, the effects of CR in increasing PCS score (4 trials; participants = 478; MD = 6.05, 95% CI = 1.77 to 10.34; I^2^ = 93%; low-quality evidence; Fig. 6), and MCS (4 trials; participants = 478; MD = 5.38, 95% CI = 1.13 to 9.63; I^2^ = 84%; low-quality evidence; Supplementary Fig. 5) were also meaningful.

### CR vs AC

As shown in the forest plots and Table 3b, for trials with AC arms, meta-analyses were performed for the following outcomes: re-hospitalizations, adverse events, CVD risk factors (i.e., BMI, BP, lipids), and QoL (8 domains of SF-36, as well as PCS and MCS scores). No significant effect of CR was found for morbidity (Supplementary Figs. 14,15), blood pressure (Supplementary Figs. 17 and 18) or lipids (Supplementary Figs. 19–22). There were meaningful effects of CR for the following outcomes: body mass index (Supplementary Fig. 16) and all QoL indicators (Supplementary Figs. 23a,b and 24a-h). For example, compared to AC, the effects of CR in decreasing BMI were significant, but likely not clinically-meaningful (3 trials; participants = 388; MD = -0.84 kg/m^2^, 95% CI = -1.61 to 0.07; I^2^ = 0%; moderate-quality evidence).

### Meta-Regression

There were only sufficient trials for the outcome of METs with UC comparison to perform meta-regression. As shown in Supplemental Fig. 26, none of the predefined study-level covariates were statistically significant (setting *p* = 0.37; program duration *p* = 0.27; total sessions/dose *p* = 0.74; multi-centre *p* = 0.79; study quality *p* = 0.76), suggesting that the benefits of CR on exercise capacity is not limited to these factors or types of programs.

### Publication Bias

There were only sufficient trials to test funnel plot symmetry for the outcome of METs for trials with UC controls. As shown in Supplemental Fig. 25, there was no evidence of funnel plot asymmetry (Egger's test *p* = 0.92).

### Qualitative Results

Other outcomes were tested that could not be pooled in meta-analysis (Table 1). CVD mortality was tested only in Babu et al.'s trial,^44^ with no significant differences between CR (*n* = 0) and UC (*n* = 1) arms. Regarding other morbidity indicators, one trial showed incidence of percutaneous coronary intervention was significantly lower with CR than UC and AC,^37^ and it was also lower in another trial with comprehensive CR compared to UC and with exercise-based CR (AC) compared to UC (no effect for bypass surgery).^35^ Non-fatal myocardial infarction and stroke were not significantly different between the yoga arm and UC in one trial.^33^ In another, myocardial infarction was significantly lower with exercise-only CR (AC) compared to UC.^35^ A summary of results from other outcomes tested are shown in the supplemental qualitative results.

### Costs and Cost-Effectiveness

Finally, two trials reported on costs.^45^^,^^46^ The trial by Salvetti et al. which found significant beneficial impacts of home-based CR on multiple outcomes, reported a low total average cost per patient in Brazil (equivalent to USD$502.71) when compared to CR costs reported globally^13^; the program comprised an average of four physician visits, four electrocardiograms (ECG) among some other diagnostic tests, two cardiopulmonary exercise tests, two exercise sessions without ECG monitoring, and telephone calls.^45^

Lima et al. reported a total cost per participant to deliver home-based CR of R$242.72, in contrast to traditional CR at R$552.73; the clinically-effective 12-week home-based program comprised 4 face-to-face sessions, and frequent telephone follow-ups promoting home walking 5 times per week.^46^ Planned economic analyses of the YogaCare^47^ trial, evaluating health expenditures and cost-effectiveness, are currently in preparation (personal communication with authors).

## Discussion

This systematic review with meta-analysis investigating effectiveness of CR in LMICs identified a total of 26 randomised controlled trials undertaken in 8 LMICs including 6380 patients with ACS or HF. There was significantly greater functional capacity and QoL, along with significant decreases in BP, lipids and BMI following CR participation compared to control. However, given the risk of bias in the trials, these improvements have low to moderate level of certainty. Furthermore, given that the majority of trials were of small sample size and short duration, inadequate number of events were reported to assess the impact of CR on mortality and non-fatal outcomes, including hospital admissions. Overall however, it can be concluded that CR has beneficial effects on several important outcomes, and that effects and effect sizes achieved in LMICs are comparable with those achieved in high-income countries.^6^

The nature of the CR programs in these trials in LMICs are consistent with that reported in the International Council of Cardiovascular Prevention and Rehabilitation (ICCPR)’s global audit.^14^ Whilst yoga has not been tested in a trial in any non-LMIC to our knowledge, it is offered in some programs.^48^ Although potentially of additional benefit, resistance exercise training was not common in the included trials. Most programs were comprehensive, although we know programs in LMICs are less so than in high-resource settings.^14^ This may be why no effect on tobacco use or depressive symptoms was observed. Dose was robust, at a median of 32 sessions, compared to 24 globally.^49^ Physiotherapists, physicians, and nurses figured prominently on CR teams. In terms of setting, most were hybrid, and likely use of mobile technology will grow.

Results of the meta-analysis suggest a clinically-meaningful impact of CR on functional capacity,^50^ with >0.5 MET increase with CR. Such an increase in cardiorespiratory fitness has been shown to be associated with reductions in mortality.^51^^,^^6^ LDL reductions reached clinical significance, but blood pressure did not. QoL differences would be considered to have a meaningful impact on the lives of patients. Qualitative results suggested CR in LMICs may also have positive effects for morbidity (percutanous coronary intervention, myocardial infarction in non-yoga trials), CV biomarkers, cardiopulmonary function (including ejection fraction), muscle strength, heart-health behavior, and psychosocial well-being.

This review points to areas where future research is needed. Included trials were only from 8/138 LMICs, with none from Europe; We estimate CR is available in 55 (39.9%) LMICs.^14^ Although results herein are consistent with effects achieved in high-income countries, and meta-regression suggested consistent beneficial effects on functional capacity at least across various program and trial characteristics, clearly more evidence is needed for many important outcomes (see below), and it would be informative to have more representative data. In particular, there are a number of programs in Colombia, Argentina, Mexico, Georgia, Turkey and South Africa, where trial data would be informative.

Future trials of CR in LMICs need to focus on the outcomes of all-cause and CVD mortality, morbidity (e.g., HF, stroke) as well as revascularization (especially surgical), symptoms (angina, dyspnea), medication adherence and costs.^12^ Quality of evidence for risk factors in trials with UC comparisons were particularly low, and so high-quality studies in that area are warranted. ICCPR recently undertook a Delphi process to develop a standard outcome measure set for their new international registry.^52^ It is hoped this publicly-available and internationally-agreed resource will promote the more consistent collection of outcomes to enable future assessment of CR impact in LMICs.

Finally, while the cost findings in the two included trials showed evidence of cost-effectiveness,^45^^,^^46^ and were consistent with other cost-effectiveness studies on CR in LMICs,^12^ they were limited to home-based CR. Collection of additional data is important to inform policy-makers which CR models are most clinically and cost-effective.

### Limitations

We believe this to be most comprehensive systematic review to date of the randomised controlled evidence assessing the impact of CR in LMIC settings. However, we recognize that our review has a number of potential limitations. First, methodological quality of included trials was limited, resulting in low-quality evidence for several outcomes, as outlined above. Second, given only a small proportion of eligible patients access to CR, results would not be generalizable to all CVD patients; it is likely more socio-economically advantaged, healthier patients are accessing CR as we see in high-income countries.^53^ Moreover, as per previous CR reviews,^6^ most participants were male, and hence generalizability to women warrants further investigation.

Third, we planned to undertake subgroup analyses, however there were only sufficient trials for the METs outcome. This is disappointing, as we are as yet not able to make firm policy recommendations around models of CR that might be particularly effective for example (e.g., yoga, mobile phone-based CR); indeed, results from the recent Yogacares trial^47^ point to the possibility that not including a formal aerobic exercise component may result in less impact on CVD events. Relatedly, because of the limited number of trials for the various outcomes, presence of publication bias could not be ruled out.

In conclusion, this systematic review and meta-analysis revealed there is low or moderate certainty evidence that participation in CR results in improved functional capacity, risk factor control, and QoL, among other benefits, for patients with ACS and HF in LMICs. Our findings support calls regarding the urgent need to augment CR capacity in LMICs, by developing and delivering affordable, accessible programs. Need is greatest in India, China, Russia, Pakistan, Brazil and Ukraine^54^; if we can increase CR access in these and the many other LMICs with CVD at epidemic levels, we can improve the outcomes of ACS and HF patients, whilst reducing the burden on economies, society, and health systems.

## Funding source

This study is a part of the ACROSS project (Affordable Cardiac Rehabilitation: An Outreach inter-disciplinary Strategic Study) funded by NIHR Research and Innovation for Global Health and Transformation (RIGHT) Call 3 Development Grant. This work was supported by the 10.13039/501100000853University of Glasgow project 311264-01. The study sponsor(s) or funder(s) played no role in the conception of this study, analysis or interpretation of data, in the writing of the manuscript, nor in the decision to submit the article for publication. Moreover, we confirm the independence of researchers from the funder and that all authors, external and internal, had full access to all of the data (including statistical reports and tables) in the study. All authors take the responsibility for data integrity and the accuracy of the data analysis.

## Conflicts of interest

None.

All authors have completed the ICMJE uniform disclosure form.

## Appendix A Supplementary data

Appendix 1 Medline Search Strategy Image 1

Supplementary Qualitative Results Image 2

Supplementary Figures Image 3
