# Supplementary material for: Effects of Cardiac Rehabilitation in Low- and Middle-Income Countries: A systematic Review and Meta-Analysis of Randomised Controlled Trials
Source: Prog Cardiovasc Dis. 2022 Jan-Feb;70:119–74. doi: 10.1016/j.pcad.2021.07.004 (PMC9187522; doi:10.1016/j.pcad.2021.07.004)
Supplement: Supplementary file 1 — Appendix 1 Medline Search Strategy [file mmc1.docx]

**Supplemental Results**

Cardiovascular biomarkers were also tested, with one trial showing significantly lower homocysteine and significantly higher apolipoprotein A1b^1^ with CR compared to UC. Another trial reported there was no change in high-sensitivity C-reactive protein and N-terminal fragment of Brain Natriuretic Peptide with CR compared to UC.^2^ With regard to blood oxygen saturation, it was significantly improved with CR compared to UC in another trial.^3^

Cardiac function was also tested in several trials. Left ventricular ejection fraction (EF) was significantly greater with CR compared to UC in one trial;^4^ another two trials reported no significant improvement in EF with CR compared to AC, suggesting it may only be impacted with exercise-based CR of some form.^5^ ^6^ There was significant improvement in diastolic function (as measured by e/a ratio) with CR in another trial of cardiomyopathy patients compared to UC.^4^ Pulmonary function tests were found significantly improved with CR but did not with UC.^7^

Heart rate variability was also assessed. Indicators were significantly improved with CR compared to UC in one trial.^8^ R-R interval variation and autonomic dysfunction was also reported to be significantly improved with CR compared to AC in one trial.^9^

With regard to cardiac symptoms, one trial reported patients with HF had significantly reduced symptoms, including pain and oedema with CR compared to UC.^10^ As for fatigue, there was no significant difference among CR participants compared to their UC counterparts in one trial.^11^ Another trial showed no effect of CR in reducing angina compared to AC or UC.^12^

Regarding other risk factor parameters, blood glucose was found to be significantly decreased with CR compared to UC in two trials,^13^ ^14^ but another trial reported non-significant change in fasting blood glucose compared to UC.^15^ Three trials reported non-significant change in fasting blood glucose compared to AC.^15^ ^6^ ^16^ HbA1_c_ was reported in two trials, with no more significant change with CR compared to UC.^16 17^

With regard to waist circumference, no significant change was observed in CR compared to UC^16^ and compared to both UC and AC.^15^ However, waist-hip ratio was found to be significantly reduced in one trial^13^ but not in another trial in CR, compared to UC.^18^

With regard to heart-healthy behaviours, diet was improved significantly with CR compared to UC and AC in one trial.^15^ With regard to consuming different diet types, there was no significant difference observed in choosing vegetarian and low-fat diets in CR compared to UC as reported in one trial.^13^ Another trial reported no difference in dietary adherence with CR compared to UC.^19^

With regard to physical activity indicators such as exercising regularly and monitoring intensity, these were reported to be significantly improved with CR compared to UC in one trial,^13^ but exercise was not better with CR compared to UC in another.^19^ Moreover, pedometer step counts were not significantly better with CR in two trials, one compared to AC,^20^ and another compared to both UC and AC.^15^ Quadricep and bicep muscle strength was significantly greater with CR than UC in one trial;^1^ no other trials reported on strength.

No significant effect on medication adherence was reported with CR in any trials, when compared either UC ^8^ ^21 18^ or AC.^20^ One trial reported significant improvement in “adherence to treatment” with CR compared to UC.^19^

Some other psychosocial outcomes were also tested. Of the 5 trials testing anxiety, two showed it was significantly reduced post-CR compared to UC,^10^ and compared to AC,^22^ while another trial reported increased anxiety with CR compared to UC.^21^ The other two trials revealed no effects: one with CR compared to UC,^23^ and the other compared to AC at 1-year as well as 5-year follow-up^24^ ^25 6^.

With regard to perceived stress, it was significantly reduced with CR compared to AC in one trial at the 5-year follow-up, but not 1-year.^25, 6^ There was significant improvement in positive, but no impact on negative affect, with CR compared to AC.^25, 6^ A significant improvement in psychosocial well-being with CR was reported in one trial compared to UC using a validated scale.^26^ Finally, there were also significant improvements in return to life roles with yoga compared to UC.^8^

With regard to knowledge, two trials reported significantly greater cardiovascular knowledge achieved with CR compared to UC^23^ and compared to both UC and AC arms.^15^ On the contrary, another study found no significant change in cardiovascular knowledge with CR compared to AC.^27^

**References**

1. Mehani SHM. Novel molecular biomarkers’ response to a cardiac rehabilitation programme in patients with ischaemic heart diseases. *Eur J Physiother*. 2018;20(4):235-243.

2. Abolahrari-Shirazi S, Kojuri J, Bagheri Z, Rojhani-Shirazi Z. Efficacy of combined endurance-resistance training versus endurance training in patients with heart failure after percutaneous coronary intervention: A randomized controlled trial. *J Res Med Sci*. 2018;23(2).

3. Ajiboye OA, Anigbogu CN, Ajuluchukwu JN, Jaja SI. Exercise training improves functional walking capacity and activity level of Nigerians with chronic biventricular heart failure. *Hong Kong Physiother J*. 2015;33(1):42-49.

4. Mehani SHM. Correlation between changes in diastolic dysfunction and health-related quality of life after cardiac rehabilitation program in dilated cardiomyopathy. *J Adv Res*. 2013;4:189-200.

5. Abdelhalem AM, Shabana AM, Onsy AM, Gaafar AE. High intensity interval training exercise as a novel protocol for cardiac rehabilitation program in ischemic Egyptian patients with mild left ventricular dysfunction. *Egypt Hear J*. 2018;70(4):287-294.

6. Raghuram N, Parachuri VR, Swarnagowri M V., et al. Yoga based cardiac rehabilitation after coronary artery bypass surgery: One-year results on LVEF, lipid profile and psychological states - A randomized controlled study. *Indian Heart J*. 2014;66(5):490-502.

7. Yadav A, Singh S, Singh K, Pai P. Effect of yoga regimen on lung functions including diffusion capacity in coronary artery disease patients: A randomized controlled study. *Int J Yoga*. 2015;8(1):62.

8. Prabhakaran D, Chandrasekaran AM, Mohan B, et al. Yoga-Based Cardiac Rehabilitation After Acute Myocardial Infarction: A Randomized Trial. *J Am Coll Cardiol*. 2020;75(13):1551-1561.

9. Venkatesh N, Kumar T, Yogeswari R, Sridevi S. Effects of Exercise on the Sympathovagal Regulation of Heart and Functional Capacity in Patients Following Coronary Artery Bypass Grafting. *Indian J Public Heal Res Dev*. 2019;10(11):509-511.

10. Jena S, Das S, Pradhan R. A comparative study between effects of aerobic exercises and conventional treatment on selected outcomes of heart failure clients. *Int J Res Pharm Sci*. 2020;11(1):342-346.

11. Hasanpour-Dehkordi A, Yadollahi M, Tali S, Gheshlagh R. Effect of exercise training on dimensions of quality of life and fatigue in people with congestive heart failure class II and III: A randomized controlled trial. *Indian J Med Spec*. 2020;11(1):15-20.

12. Chaves GSS, Ghisi GLM, Britto RR, Grace SL. Maintenance of gains, morbidity, and mortality at 1 year following cardiac rehabilitation in a middle-income country: A wait-list control crossover trial. *J Am Heart Assoc*. 2019;8(4):e011228.

13. Naser A, Shahamfar J, Kumar G V, et al. Cardiac risk factor changes through an intensive multifactorial life style modification program in CHD patients: Results from a two year follow up. *J Biol Sci*. 2008;8(2):248-257.

14. Hassan A, Nahas N. Efficacy of cardiac rehabilitation after percutaneous coronary intervention. *Int J Pharm Res*. 2016;9:134-141.

15. Chaves G, Ghisi GLM, Grace SL, et al. Effects of comprehensive cardiac rehabilitation on functional capacity in a middle-income country: a randomised controlled trial. *Heart*. 2019;105(5):406-413.

16. Limaa AP, Nascimento IO, Martinsa TH, et al. Analysis of adherence, effectiveness and cost of cardiac rehabilitation at home in a middle-income country: Randomized clinical trial. *PhD Diss*. 2020.

17. Uddin J, Joshi VL, Moniruzzaman M, et al. Effect of Home-Based Cardiac Rehabilitation in a Lower-Middle Income Country: Results from a Controlled Trial. *J Cardiopulm Rehabil Prev*. 2020;40(1):29-34.

18. Dorje T, Zhao G, Scheer A, et al. SMARTphone and social media-based Cardiac Rehabilitation and Secondary Prevention (SMART-CR/SP) for patients with coronary heart disease in China: A randomised controlled trial protocol. *BMJ Open*. 2018;8:e021908.

19. Soleimani F, Anbohi SZ, Esmaeili R, Pourhoseingholi MA, Borhani F. Person-centered Nursing to Improve Treatment Regimen Adherence in Patients with Myocardial Infarction. *J Clin Diagnostic Res*. 2018;12(1).

20. Passaglia L, Nascimento BR, Brant LC, Ribeiro AL. Impact of Text Messages in a Middle-income Country to Promote Secondary Prevention After Acute Coronary Syndrome (IMPACS): A Randomized Trial. *J Am Coll Cardiol*. 2020;75(11):2003.

21. Zhang L, Zhang L, Wang J, Ding F, Zhang S. Community health service center-based cardiac rehabilitation in patients with coronary heart disease: a prospective study. *BMC Health Serv Res*. 2017;17(1):128.

22. Dehdari T, Heidarnia A, Ramezankhani A, Sadeghian S, Ghofranipour F. Effects of progressive muscular relaxation training on quality of life in anxious patients after coronary artery bypass graft surgery. *Indian J Med Res*. 2009;129(5):603-608.

23. Dorje T, Zhao G, Tso K, et al. Smartphone and social media-based cardiac rehabilitation and secondary prevention in China (SMART-CR/SP): a parallel-group, single-blind, randomised controlled trial. *Lancet Digit Heal*. 2019;1(7):e363-e374.

24. Eraballi A, Raghuram N, Ramarao NH, Pradhan B, Rao PV. Yoga Based Lifestyle Program in Improving Quality of Life after Coronary Artery Bypass Graft Surgery: A Randomised Controlled Trial. *J Clin Diagnostic Res*. 2018;12:5-9.

25. Amaravathi E, Ramarao NH, Raghuram N, Pradhan B. Yoga-Based Postoperative Cardiac Rehabilitation Program for Improving Quality of Life and Stress Levels: Fifth-Year Follow-up through a Randomized Controlled Trial. *Int J Yoga*. 2020;11(1):44-52.

26. Ul-Haq Z, Khan D, Hisam A, et al. Effectiveness of Cardiac Rehabilitation on Health-related Quality of Life in Patients with Myocardial Infarction in Pakistan. *J Coll Physicians Surg - Pakistan*. 2019;29(9):803-809.

27. Passaglia LG, Brant LCC, Nascimento BR, Ribeiro ALP. Impact of text messages in a middle-income country to promote secondary prevention after acute coronary syndrome (IMPACS): A randomized trial. *Medicine (Baltimore)*. 2019;98(22):e15681.
