# Supplementary material for: Effects of Cardiac Rehabilitation in Low- and Middle-Income Countries: A systematic Review and Meta-Analysis of Randomised Controlled Trials
Source: Prog Cardiovasc Dis. 2022 Jan-Feb;70:119–74. doi: 10.1016/j.pcad.2021.07.004 (PMC9187522; doi:10.1016/j.pcad.2021.07.004)
Supplement: Supplementary file 2 — Supplementary Qualitative Results [file mmc2.docx]

**Appendix-1 Medline Search Strategy**

Search Strategy: Ovid MEDLINE(R) ALL <1946 to May 12, 2020>

--------------------------------------------------------------------------------

1 [Population: Heart Disease Patients]

2 exp Heart Diseases/

3 exp Myocardial Revascularization/

4 ((heart or cardiac or coronary) adj3 (disease* or disorder* or patient*)).tw,kw.

5 acute coronary syndrome.tw,kw.

6 ((heart or cardiac) adj3 (arrest* or infarct* or failure)).tw,kw.

7 (myocardi* adj3 (infarct* or ischemi* or ischaemi* or revascularization or revascularisation)).tw,kw.

8 (coronary adj3 (bypass or angioplast* or percutaneous or acute)).tw,kw.

9 (angina* or ischaemi* or ischemi* or carditis* or atherosclero*).tw,kw.

10 (implant* adj3 (ventricular or VAD?)).tw,kw.

11 (ventricular adj3 (revascularization or revascularisation)).tw,kw.

12 (rehab* adj3 (cardiac or cardiovascular)).tw,kw.

13 or/2-12

14 [Intervention: Cardiac Rehabilitation]

15 Cardiac Rehabilitation/

16 exp Exercise/

17 exp Exercise Movement Techniques/

18 exp Exercise Therapy/

19 (exercis* or kinesiotherap* or kinesitherap*).tw,kw.

20 (training adj4 (resistanc* or weight* or strength* or interval* or intermittent* or cardio* or aerobic)).tw,kw.

21 (rehab* adj3 (cardiac or cardiovascular)).tw,kw.

22 or/15-21

23 13 and 22

24 Aborigin*.af.

25 Afghan*.af.

26 african continental ancestry group/

27 american native continental ancestry group/

28 Armenia*.af.

29 Bangladesh*.af.

30 (Basutoland or Basuto*).af

31 Benin*.af.

32 Bhutan*.af.

33 Bolivia*.af.

34 "Burkina Faso".af.

35 (Burma or Burmes*).af.

36 Burundi*.af.

37 Cabo Verde*.af.

38 Cambodia*.af.

39 Cameroon*.af.

40 Cape Verde*.af.

41 "Central African Republic".af.

42 Central America*.af.

43 Ceylon.af.

44 Chad*.af.

45 Comoro Islands.af.

46 (Comoros or Comorian*).af.

47 Congo*.af.

48 "Cote d'Ivoire".af.

49 Djibouti*.af.

50 Egypt*.af.

51 El Salvador*.af.

52 Eritrea*.af.

53 Ethiopia*.af.

54 "French Somaliland".af.

55 Gambia*.af.

56 Gaza*.af.

57 Georgia*.af.

58 Ghana*.af.

59 "Gold Coast".af.

60 Guatemala*.af.

61 Guinea*.af.

62 Guniea-Bisau*.af.

63 Haiti*.af.

64 (Honduras or Honduran*).af.

65 "Ifni".af.

66 India*.af.

67 indigenous.af.

68 Indonesia*.af.

69 Ivory Coast.af.

70 Kenya*.af.

71 Kirghizia*.af.

72 (Kirghiz or Kyrgyz).af.

73 Kirgizstan*.af.

74 Kiribati.af.

75 ((Democratic People's Republic adj2 Korea) or North Korea*).af.

76 Kosovo.af.

77 Kyrgyzstan.af.

78 "Kyrgyz Republic".af.

79 "Lao PDR".af.

80 (Laos or Laotian*).af.

81 Latin America*.af.

82 (Lesotho or Mosotho*).af.

83 Liberia*.af.

84 (Madagascar or Malagasy).af.

85 Malawi*.af.

86 Mali*.af.

87 Mauritania*.af.

88 Mayotte.af.

89 Melanesia*.af.

90 Micronesia*.af.

91 Moldova.af.

92 Moldovia*.af.

93 (Morocco or Moroccan*).af.

94 (Mozambique or Mozambican*).af.

95 Myanmar*.af.

96 Nepal*.af.

97 New Caledonia*.af.

98 Nicaragua*.af.

99 Niger*.af.

100 Nigeria*.af.

101 Nyasaland.af.

102 oceanic ancestry group/

103 Pakistan*.af.

104 Papua New Guinea.af.

105 (Philipines or Filipinas or Filipino*).af.

106 Philippines.af.

107 Phillipines.af.

108 Phillippines.af.

109 "Ruanda-Urundi".af.

110 Rwanda*.af.

111 Samoa*.af.

112 Sao Tome*.af

113 "Samoan Islands".af.

114 Senegal*.af.

115 "Sierra Leone".af.

116 Sri Lanka*.af.

117 "Solomon Islands".af.

118 Somalia*.af.

119 Sudan*.af.

120 (Swaziland or Swazi*).af.

121 Syria*.af.

122 Tadjikistan*.af.

123 Tadzhik*.af.

124 Tadzhikistan*.af.

125 Tajikistan*.af.

126 Tanzania*.af.

127 Timor-Leste.af.

128 Togo*.af.

129 "Togolese Republic".af.

130 Uganda*.af.

131 Ukrain*.af.

132 "Upper Volta".af.

133 Urundi.af.

134 Uzbek*.af.

135 Uzbekistan.af.

136 (Vanuatu or Ni-Vanuatu).af.

137 "Viet Nam".af.

138 Vietnam*.af.

139 "West Bank".af.

140 Yemen*.af.

141 Zaire.af.

142 Zambia*.af.

143 Zimbabwe*.af.

144 or/24-143

145 Developing Countries/

146 Medically Underserved Area/

147 ((countr* or nation* or population*) adj3 (poor* or developing or less-develop* or less develop* or least-develop* or least develop* or third world or under-develop* or under develop*)).tw,kw.

148 (income* adj3 (low* or middle*)).tw,kw.

149 (underserved or deprived or third world*).tw,kw.

150 (resource* adj3 (low* or constrain* or poor* or limit* or minimal* or lack* or scarc*)).tw,kw.

151 or/145-150

152 144 or 151

153 23 and 152

154 [Study Types: RCTs]

155 exp clinical trial/

156 random*.mp.

157 (trial* adj5 (random* or control*)).mp.

158 (clinical trial, all or clinical trial or controlled clinical trial or pragmatic clinical trial or randomized controlled trial or randomised controlled trial or "research support, non us gov't").pt.

159 or/155-158

160 153 and 159

* Results were then limited to 2018 – May 12, 2020 after they were compiled in EndNote.

***************************
