# Supplementary material for: Effects of Cardiac Rehabilitation in Low- and Middle-Income Countries: A systematic Review and Meta-Analysis of Randomised Controlled Trials
Source: Prog Cardiovasc Dis. 2022 Jan-Feb;70:119–74. doi: 10.1016/j.pcad.2021.07.004 (PMC9187522; doi:10.1016/j.pcad.2021.07.004)
Supplement: Supplementary file 3 — Supplementary Figures [file mmc3.docx]

Supplementary Figure 1: Risk of bias summary for each included trial


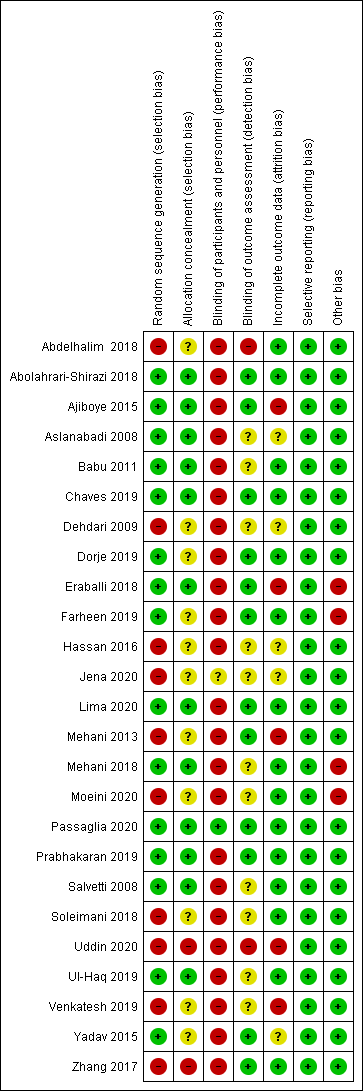


Supplementary Figure 2: Forest plot summarizing effect of CR versus UC on Functional capacity METs


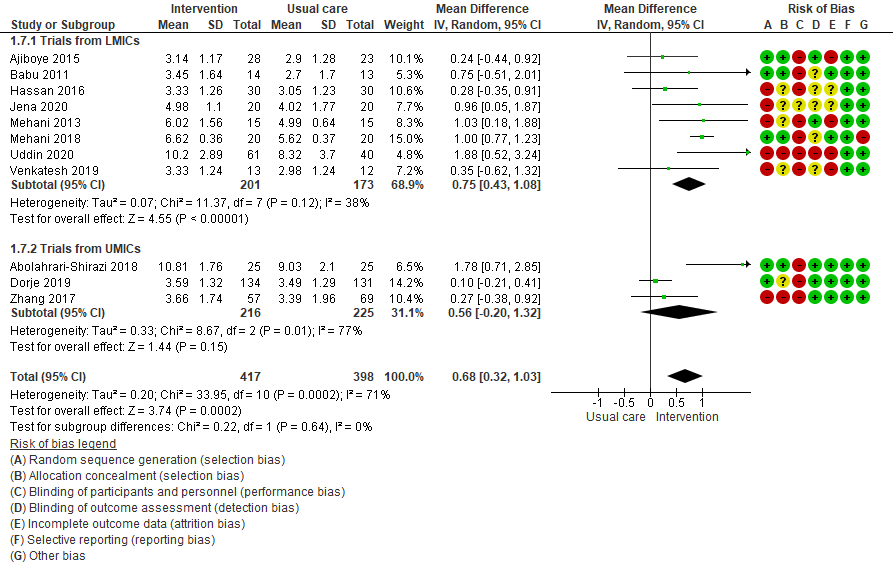


CR, cardiac rehabilitation; UC, usual care; METs, Metabolic equivalent of tasks; LMICs, Lower-middle income countries; UMICs, Upper-middle income countries

Notes: Compared with usual care, the effects of CR were meaningful in increasing functional capacity by METs (11 trials; participants=815; MD=0.68, 95% CI=0.32-1.03; low-quality evidence). Heterogeneity was high (I^2^= 71%).

Supplementary Figure 3: Forest plot summarizing effect of CR versus UC on diastolic blood pressure


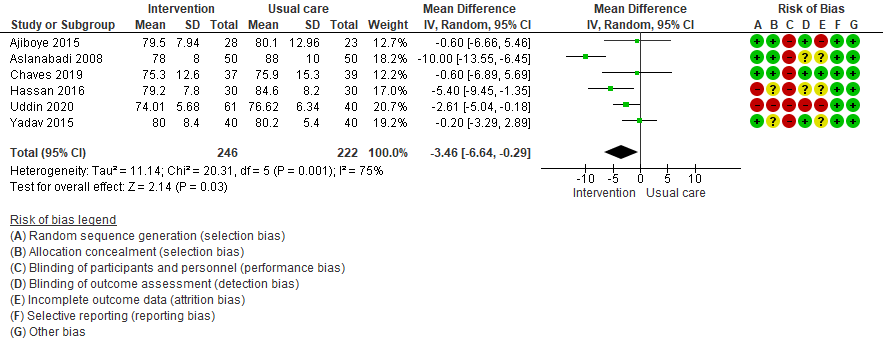


CR, cardiac rehabilitation; UC, usual care

Notes: Compared with usual care, the effects of CR were meaningful in reducing diastolic blood pressure (6 trials; participants=468; mean difference [MD]=-3.46 mmHg, 95% confidence interval [CI]=-6.64- -0.29; very low-quality evidence). Heterogeneity was high (I^2^= 75%).

Supplementary Figure 4: Forest plot summarizing effect of CR versus UC on Total Cholesterol


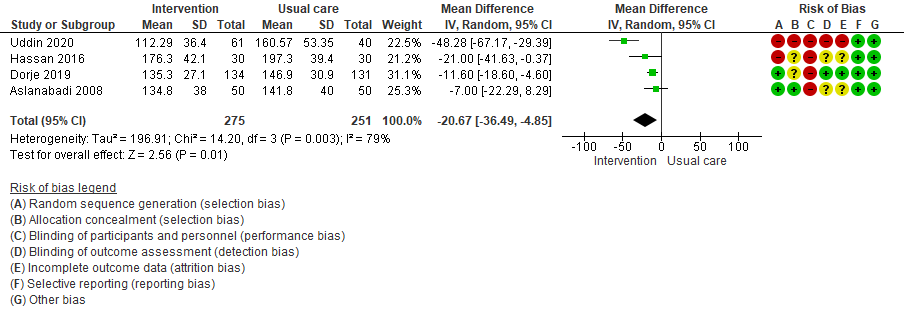


CR, cardiac rehabilitation; UC, usual care

Notes: Compared with usual care, the effects of CR were meaningful in reducing total cholesterol (4 trials; participants=526; MD=-20.67 mg/dl, 95% CI=-36.49- -4.85; very low-quality evidence). Heterogeneity was high (I^2^= 79%).

Supplementary Figure 5: Forest plot summarizing effect of CR versus UC on mental component of QoL SF-12/36 MCS


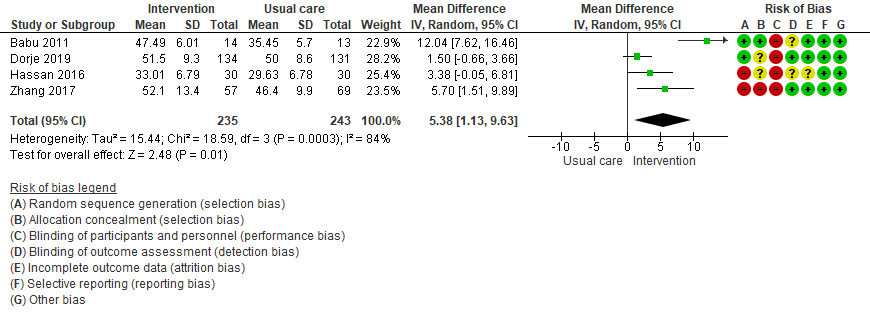


CR, cardiac rehabilitation; UC, usual care; QoL, Quality of life; SF-12/36, Short form questionnaire 12 or 36; MCS, mental component summary

Notes: Compared with usual care, the effects of CR were meaningful in increasing mental component of QoL (4 trials; participants=478; MD=5.38, 95% CI=1.13-9.63; low-quality evidence). Heterogeneity was high (I^2^= 84%).

Supplementary Figure 6: Forest plot summarizing effect of CR versus UC on total mortality


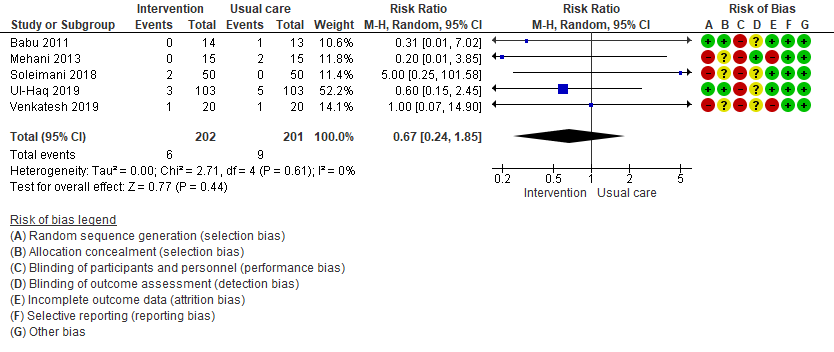


CR, cardiac rehabilitation; UC, usual care

Notes: Compared with UC, the effects of CR in reducing total mortality was not meaningful (5 trials; participants=403; Relative risk [RR]=0.67, 95% confidence interval [CI] 0.24 to 1.85; moderate-quality evidence). Heterogeneity was low (I^2^= 0%).

Supplementary Figure 7: Forest plot summarizing effect of CR versus UC on re-hospitalization


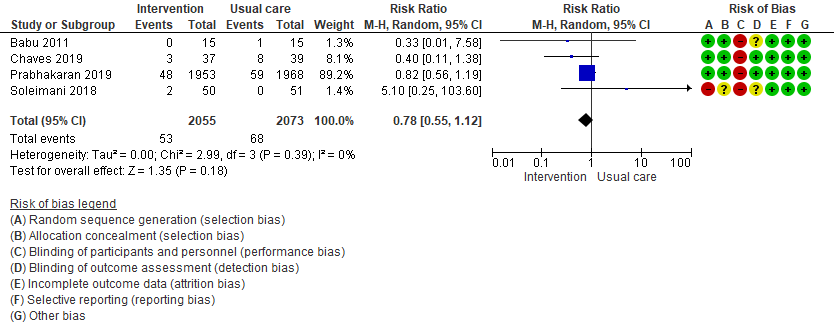


CR, cardiac rehabilitation; UC, usual care

Notes: Compared with UC, the effects of CR in reducing re-hospitalization was not meaningful (4 trials; participants=4,128; RR=0.78, 95% CI 0.55 to 1.12; moderate-quality evidence). Heterogeneity was low (I^2^= 0%).

Supplementary Figure 8: Forest plot summarizing effect of CR versus UC on Adverse events


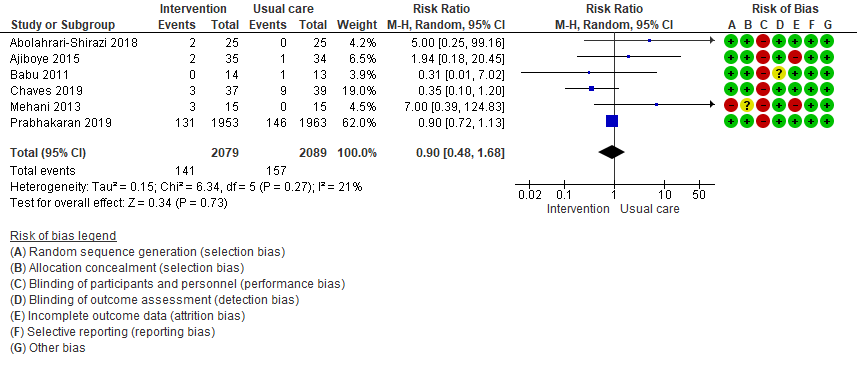


CR, cardiac rehabilitation; UC, usual care

Notes: Compared with UC, the effects of CR in reducing adverse events were not meaningful (6 trials; participants=4,128; RR=0.90, 95% CI 0.48 to 1.68; moderate-quality evidence). Heterogeneity was low (I^2^= 21%).

Supplementary Figure 9: Forest plot summarizing effect of CR versus UC on BMI


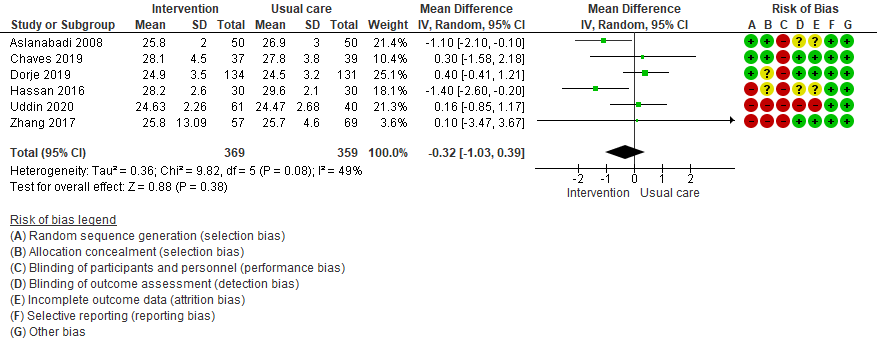


CR, cardiac rehabilitation; UC, usual care; BMI, Body mass index

Notes: Compared with usual care, the effects of CR were not meaningful in decreasing BMI (6 trials; participants=728; MD=-0.32, 95% CI=-1.03-0.39; low-quality evidence). Heterogeneity was low (I^2^= 49%).

Supplementary Figure 10: Forest plot summarizing effect of CR versus UC on Triglycerides


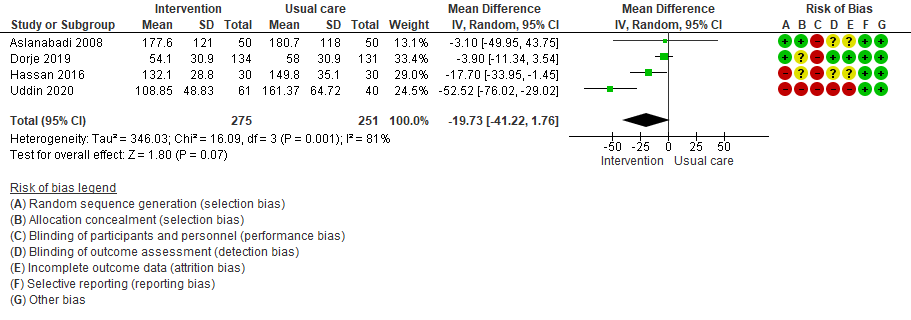


CR, cardiac rehabilitation; UC, usual care

Notes: Compared with usual care, the effects of CR were not meaningful in decreasing triglycerides (4 trials; participants=526; MD=-19.73, 95% CI=-41.22-1.76; low-quality evidence). Heterogeneity was high (I^2^= 81%).

Supplementary Figure 11: Forest plot summarizing effect of CR versus UC on HDL-cholesterol


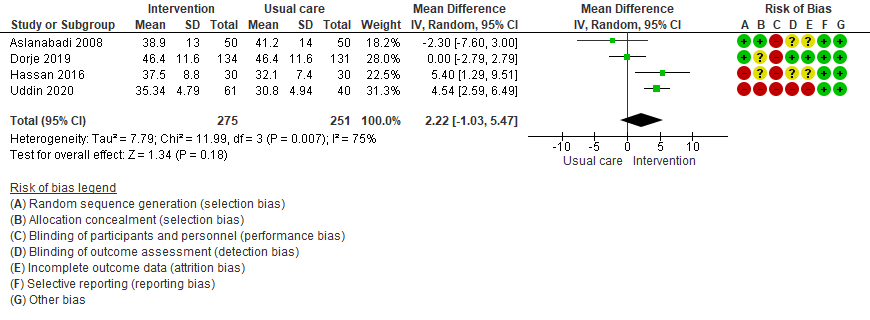


CR, cardiac rehabilitation; UC, usual care; HDL-cholesterol, High density lipoprotein cholesterol

Notes: Compared with usual care, the effects of CR were not meaningful in increasing HDL-cholesterol (4 trials; participants=526; MD=2.22, 95% CI=-1.03-5.47; low-quality evidence). Heterogeneity was high (I^2^= 75%).

Supplementary Figure 12: Forest plot summarizing effect of CR versus UC on Tobacco use UC


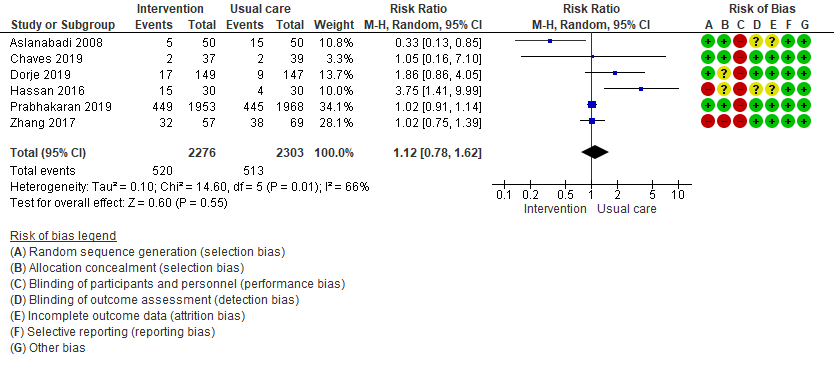


CR, cardiac rehabilitation; UC, usual care

Notes: Compared with UC, the effects of CR in reducing tobacco use were not meaningful (6 trials; participants=4,579; RR=1.12, 95% CI 0.78- 1.62; moderate-quality evidence). Heterogeneity was high (I^2^= 66%).

Supplementary Figure 13: Forest plot summarizing effect of CR versus UC on Depressive symptoms: PHQ-9


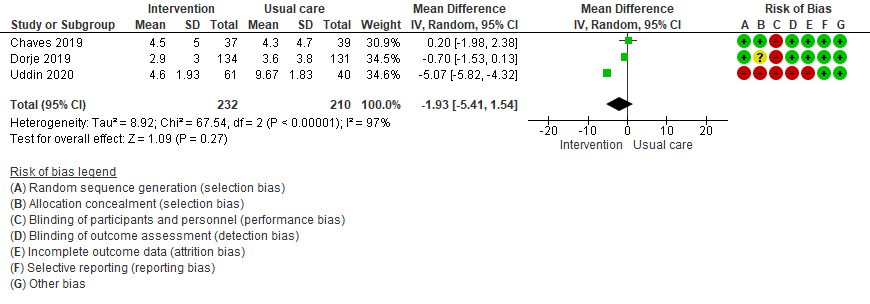


CR, cardiac rehabilitation; UC, usual care; PHQ-9, Patient Health Questionnaire-9

Notes: Compared with usual care, the effects of CR were not meaningful in decreasing depressive symptoms measured by PHQ-9 (3 trials; participants=442; MD=-1.93, 95% CI=-5.41-1.54; low-quality evidence). Heterogeneity was high (I^2^= 97%).

Supplementary Figure 14: Forest plot summarizing effect of CR versus AC on Rehospitalization


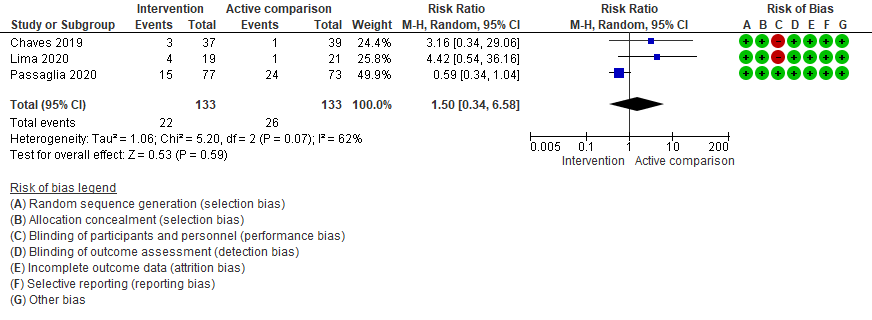


CR, cardiac rehabilitation; AC, Active comparison

Notes: Compared with AC, the effects of CR in reducing re-hospitalization were not meaningful (3 trials; participants=266; RR=1.50, 95% CI 0.34- 6.58; moderate-quality evidence). Heterogeneity was high (I^2^= 62%).

Supplementary Figure 15: Forest plot summarizing effect of CR versus AC on Adverse events


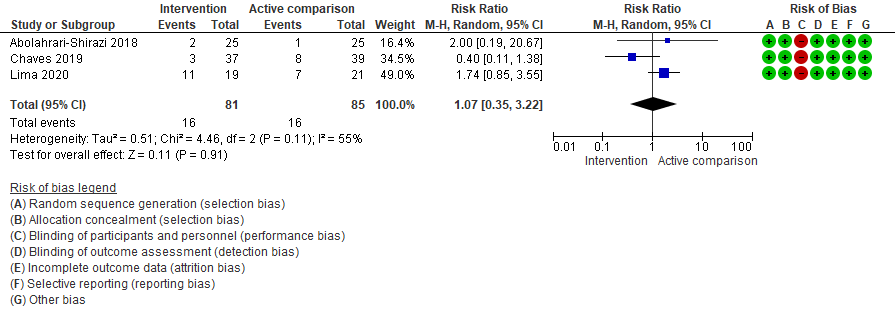


CR, cardiac rehabilitation; AC, Active comparison

Notes: Compared with AC, the effects of CR in reducing adverse events were not meaningful (3 trials; participants=166; RR=1.07, 95% CI 0.35- 3.22; moderate-quality evidence). Heterogeneity was high (I^2^= 55%).

Supplementary Figure 16: Forest plot summarizing effect of CR versus AC on BMI


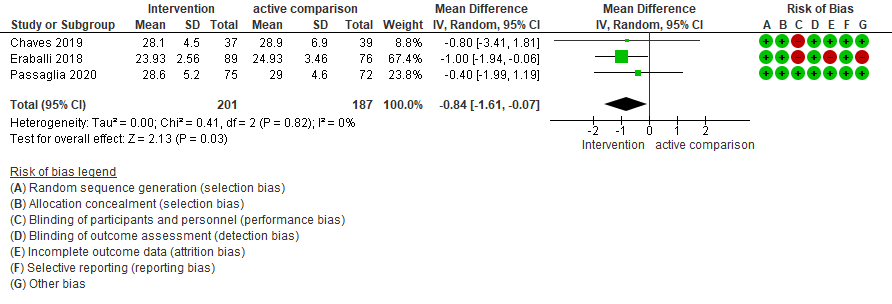


CR, cardiac rehabilitation; AC, Active comparison; BMI, Body mass index

Notes: Compared with AC, the effects of CR in reducing body mass index was meaningful (trials 6; participants = 388; MD -0.84, 95% CI -1.61 - -0.07; I^2^= 0%; moderate-quality evidence). Heterogeneity was low.

Supplementary Figure 17: Forest plot summarizing effect of CR versus AC on systolic blood pressure


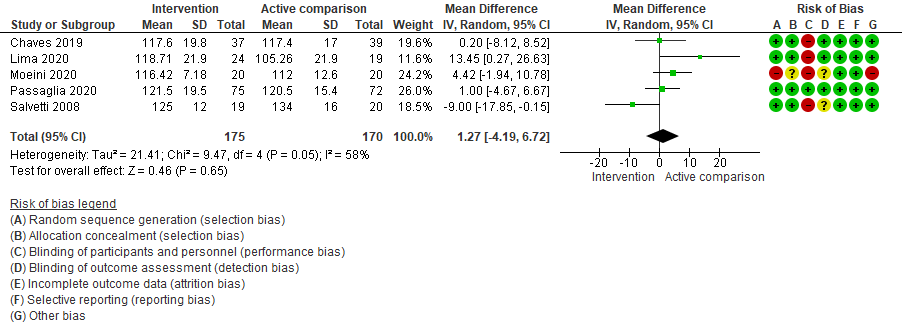


CR, cardiac rehabilitation; AC, Active comparison

Notes: Compared with AC, the effects of CR in reducing systolic blood pressure was not meaningful (trials 5; participants = 345; MD 1.27, 95% CI -4.19 – 6.72; low-quality evidence). Heterogeneity was high (I^2^= 58%).

Supplementary Figure 18: Forest plot summarizing effect of CR versus AC on diastolic blood pressure


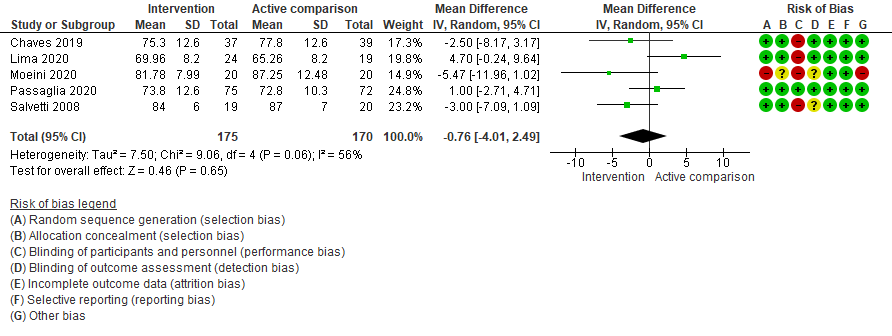


CR, cardiac rehabilitation; AC, Active comparison

Notes: Compared with AC, the effects of CR in reducing diastolic blood pressure was not meaningful (trials 5; participants = 345; MD -0.76, 95% CI -4.01 – 2.49; low-quality evidence). Heterogeneity was high (I^2^= 56%).

Supplementary Figure 19: Forest plot summarizing effect of CR versus AC on total Cholesterol


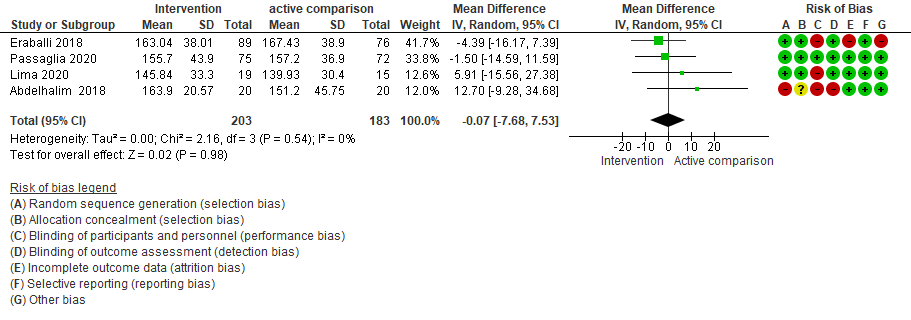


CR, cardiac rehabilitation; AC, Active comparison

Notes: Compared with AC, the effects of CR in reducing total cholesterol was not meaningful (trials 4; participants = 386; MD -0.07, 95% CI -7.68 – 7.53; low-quality evidence). Heterogeneity was low (I^2^= 0%).

Supplementary Figure 20: Forest plot summarizing effect of CR versus AC on LDL-Cholesterol


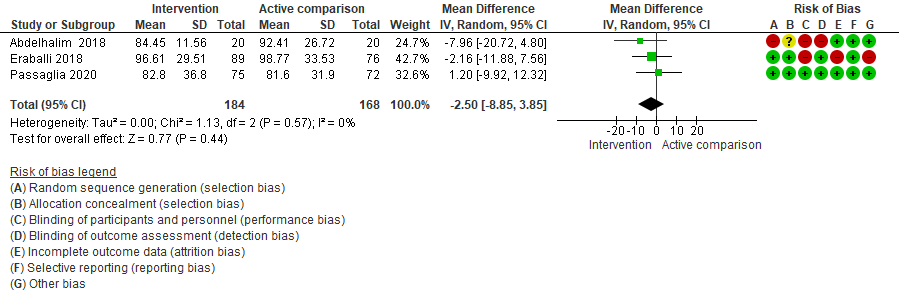


CR, cardiac rehabilitation; AC, Active comparison; LDL-cholesterol, Low density lipoprotein-cholesterol

Notes: Compared with AC, the effects of CR in reducing LDL-cholesterol was not meaningful (trials 3; participants = 352; MD -2.50, 95% CI -8.85 – 3.85; low-quality evidence). Heterogeneity was low (I^2^= 0%).

Supplementary Figure 21: Forest plot summarizing effect of CR versus AC on HDL-cholesterol


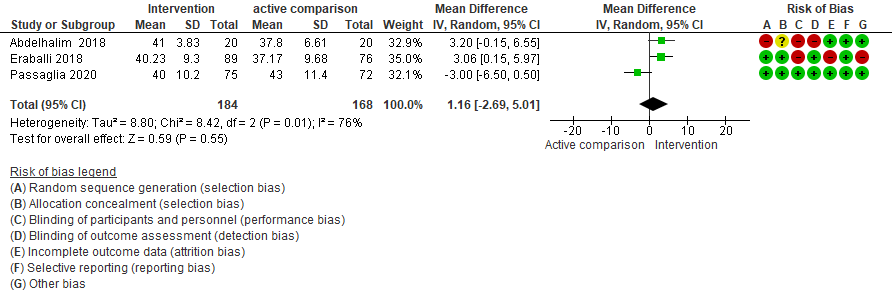


CR, cardiac rehabilitation; AC, Active comparison; HDL-cholesterol, High density lipoprotein-cholesterol

Notes: Compared with AC, the effects of CR in increasing HDL-cholesterol was not meaningful (trials 3; participants = 352; MD 1.16, 95% CI -2.69 – 5.01; low-quality evidence). Heterogeneity was high (I^2^= 76%).

Supplementary Figure 22: Forest plot summarizing effect of CR versus AC on Triglycerides


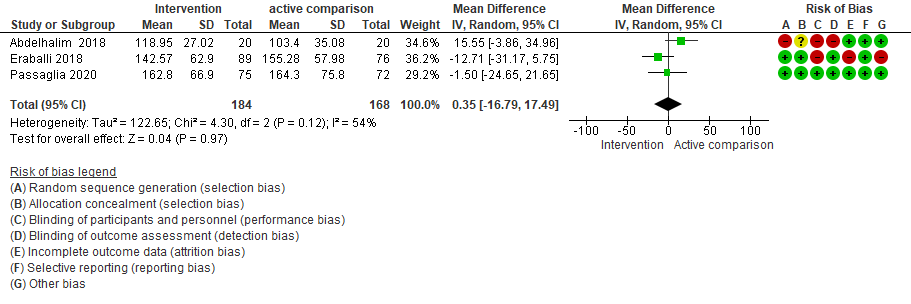


CR, cardiac rehabilitation; AC, Active comparison

Notes: Compared with AC, the effects of CR in reducing triglycerides was not meaningful (trials 3; participants = 352; MD 0.35, 95% CI -16.79 – 17.49; low-quality evidence). Heterogeneity was high (I^2^= 54%).

Supplementary Figure 23a: Forest plot summarizing effect of CR versus AC on PCS of QoL by SF-12/36


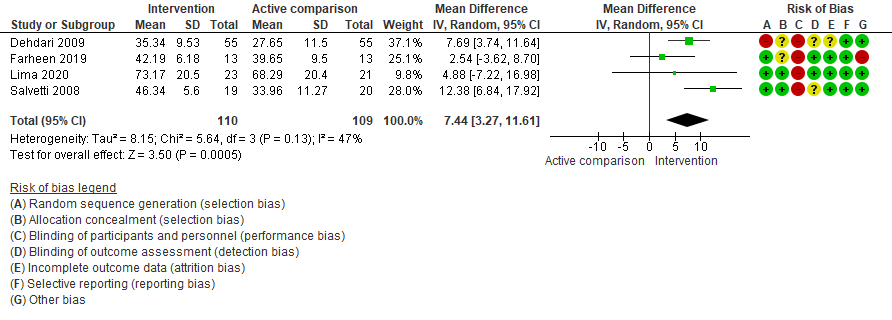
CR, cardiac rehabilitation; AC, Active comparison; PCS, Physical component summary; SF-12/36, Short form questionnaire 12/36; QoL, quality of life

Notes: Compared with AC, the effects of CR in increasing physical component of QoL by SF-12/36 was meaningful (trials 4; participants = 219; MD 7.44, 95% CI 3.27 – 11.61; low-quality evidence). Heterogeneity was low (I^2^= 47%).

Supplementary Figure 23b: Forest plot summarizing effect of CR versus AC on QoL SF-12/36 MCS


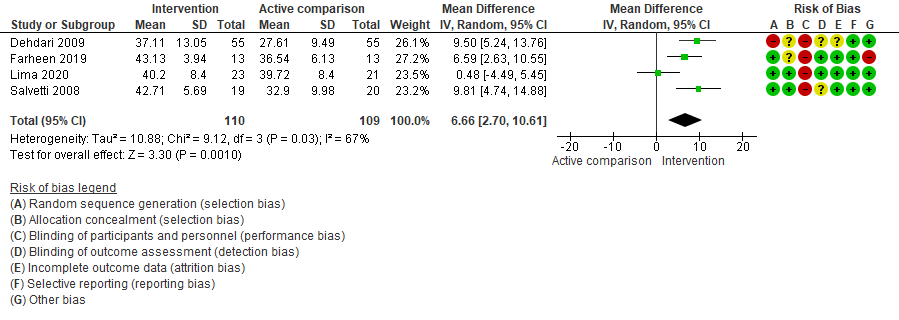


CR, cardiac rehabilitation; AC, Active comparison; MCS, Mental component summary; SF-12/36, Short form questionnaire 12/36; QoL, quality of life

Notes: Compared with AC, the effects of CR in increasing mental component of QoL by SF-12/36 was meaningful (trials 4; participants = 219; MD 6.66, 95% CI 2.70 – 10.61; low-quality evidence). Heterogeneity was high (I^2^= 67%).

Supplementary Figure 24a: Forest plot summarizing effect of CR versus AC on physical functioning domain of QoL SF-36


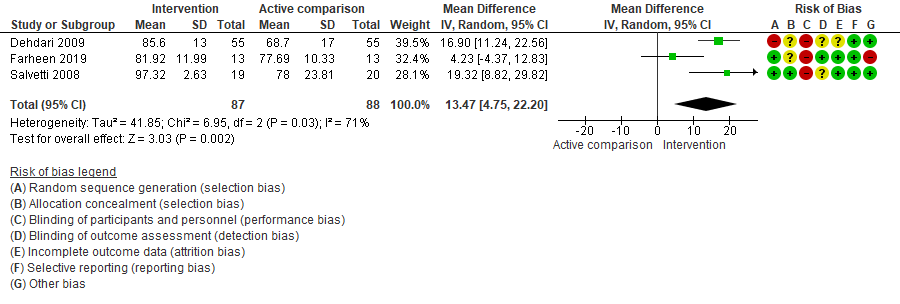


CR, cardiac rehabilitation; AC, Active comparison; SF-36, Short form questionnaire 36; QoL, quality of life

Notes: Compared with AC, the effects of CR in increasing physical functioning domain of QoL by SF-36 was meaningful (trials 3; participants = 175; MD 13.47, 95% CI 4.75 – 22.20; low-quality evidence). Heterogeneity was high (I^2^= 71%).

Supplementary Figure 24b: Forest plot summarizing effect of CR versus AC on role physical domain of QoL SF-36


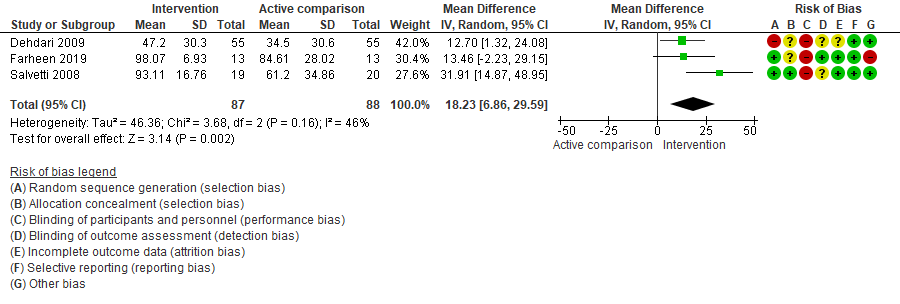


CR, cardiac rehabilitation; AC, Active comparison; SF-36, Short form questionnaire 36; QoL, quality of life

Notes: Compared with AC, the effects of CR in increasing role physical domain of QoL by SF-36 was meaningful (trials 3; participants = 175; MD 18.23, 95% CI 6.86 – 29.59; low-quality evidence). Heterogeneity was low (I^2^= 46%).

Supplementary Figure 24c: Forest plot summarizing effect of CR versus AC on bodily pain domain of QoL by SF-36


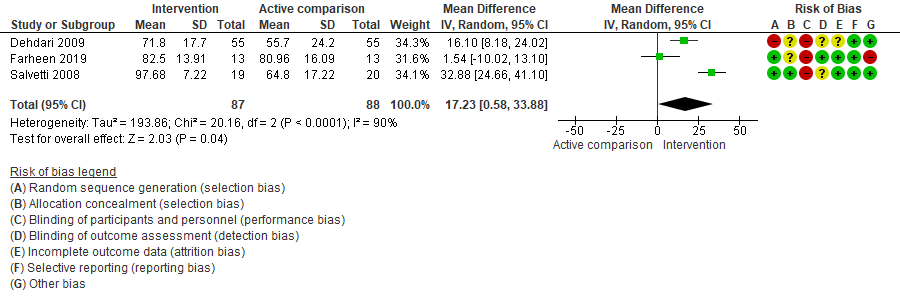


CR, cardiac rehabilitation; AC, Active comparison; SF-36, Short form questionnaire 36; QoL, quality of life

Notes: Compared with AC, the effects of CR in improving bodily pain domain of QoL by SF-36 was meaningful (trials 3; participants = 175; MD 17.23, 95% CI 0.58 – 33.88; low-quality evidence). Heterogeneity was high (I^2^= 90%).

Supplementary Figure 24d: Forest plot summarizing effect of CR versus AC on general health domain of QoL by SF-36


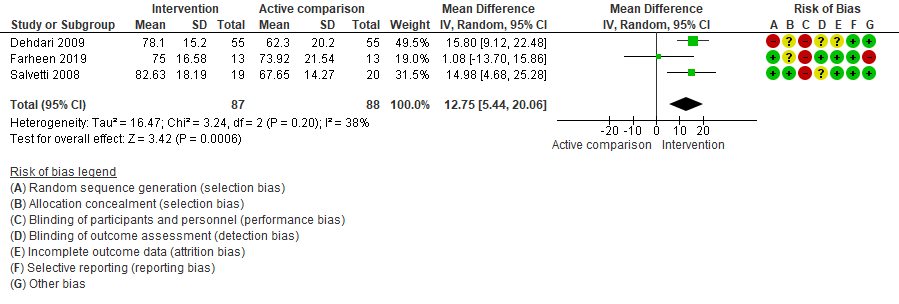


CR, cardiac rehabilitation; AC, Active comparison; SF-36, Short form questionnaire 36; QoL, quality of life

Notes: Compared with AC, the effects of CR in increasing general health domain of QoL by SF-36 was meaningful (trials 3; participants = 175; MD 12.75, 95% CI 5.44 – 20.06; low-quality evidence). Heterogeneity was low (I^2^= 38%).

Supplementary Figure 24e: Forest plot summarizing effect of CR versus AC on vitality domain of QoL by SF-36


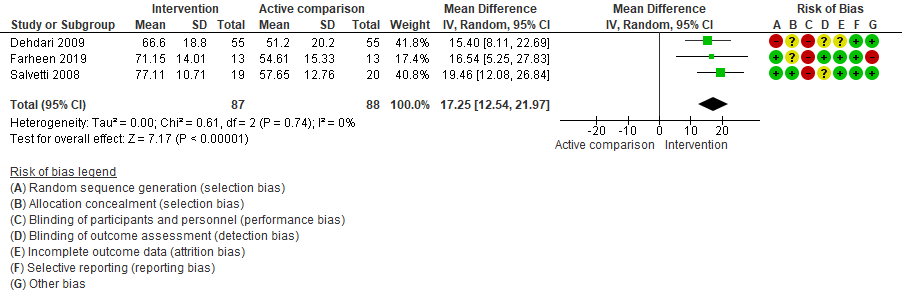


CR, cardiac rehabilitation; AC, Active comparison; SF-36, Short form questionnaire 36; QoL, quality of life

Notes: Compared with AC, the effects of CR in increasing vitality domain of QoL by SF-36 was meaningful (trials 3; participants = 175; MD 17.25, 95% CI 12.54 – 21.97; low-quality evidence). Heterogeneity was low (I^2^= 0%).

Supplementary Figure 24f: Forest plot summarizing effect of CR versus AC on role emotional domain of QoL by SF-36


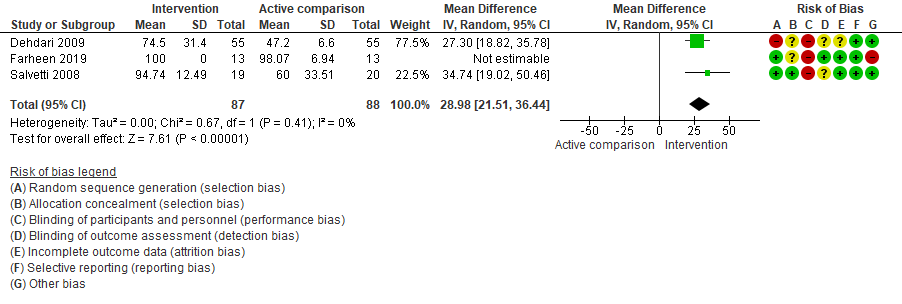


CR, cardiac rehabilitation; AC, Active comparison; SF-36, Short form questionnaire 36; QoL, quality of life

Notes: Compared with AC, the effects of CR in increasing role emotional domain of QoL by SF-36 was meaningful (trials 3; participants = 175; MD 28.98, 95% CI 21.51 – 36.44; low-quality evidence). Heterogeneity was low (I^2^= 0%).

Supplementary Figure 24g: Forest plot summarizing effect of CR versus AC on social functioning domain of QoL by SF-36


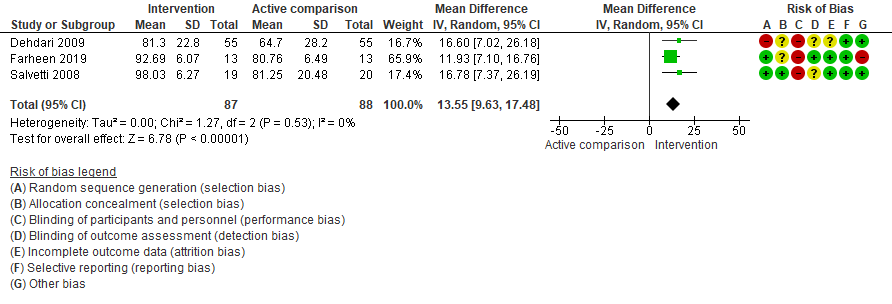


CR, cardiac rehabilitation; AC, Active comparison; SF-36, Short form questionnaire 36; QoL, quality of life

Notes: Compared with AC, the effects of CR in increasing social functioning domain of QoL by SF-36 was meaningful (trials 3; participants = 175; MD 13.55, 95% CI 9.63 – 17.48; low-quality evidence). Heterogeneity was low (I^2^= 0%).

Supplementary Figure 24h: Forest plot summarizing effect of CR versus AC on mental health domain of QoL by SF-36


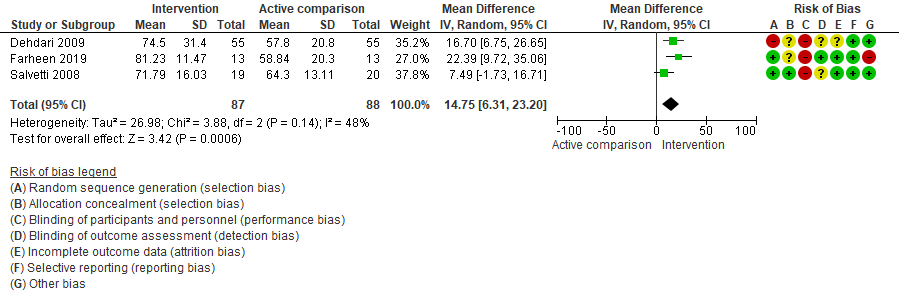


CR, cardiac rehabilitation; AC, Active comparison; SF-36, Short form questionnaire 36; QoL, quality of life

Notes: Compared with AC, the effects of CR in increasing mental health domain of QoL by SF-36 was meaningful (trials 3; participants = 175; MD 14.75, 95% CI 6.31 – 23.20; low-quality evidence). Heterogeneity was low (I^2^= 48%).

Supplemental Figure 25: Funnel plot of CR versus UC control for the outcome METs

CR, cardiac rehabilitation; UC, usual care; METs, Metabolic equivalent of tasks;

Supplemental Figure 26: Meta-regression analyses of CR versus UC control for the outcome METs
